# Prebiotic proanthocyanidins inhibit bile reflux–induced esophageal adenocarcinoma through reshaping the gut microbiome and esophageal metabolome

**DOI:** 10.1172/jci.insight.168112

**Published:** 2024-02-08

**Authors:** Katherine M. Weh, Connor L. Howard, Yun Zhang, Bridget A. Tripp, Jennifer L. Clarke, Amy B. Howell, Joel H. Rubenstein, Julian A. Abrams, Maria Westerhoff, Laura A. Kresty

**Affiliations:** 1 Department of Surgery, Section of Thoracic Surgery, and; 2 Rogel Comprehensive Cancer Center, University of Michigan, Ann Arbor, Michigan, USA.; 3 Department of Electrical and Computer Engineering, and; 4 Department of Statistics, Department of Food Science Technology, Quantitative Life Sciences Initiative, University of Nebraska-Lincoln, Lincoln, Nebraska, USA.; 5 Marucci Center for Blueberry and Cranberry Research, Rutgers University, Chatsworth, New Jersey, USA.; 6 Division of Gastroenterology, Department of Internal Medicine, University of Michigan, Ann Arbor, Michigan, USA.; 7 LTC Charles S. Kettles Veterans Affairs Medical Center, Ann Arbor, Michigan, USA.; 8 Department of Medicine, Columbia University Irving Medical Center, New York, New York, USA.; 9 Department of Pathology, University of Michigan, Ann Arbor, Michigan, USA.

**Keywords:** Gastroenterology, Oncology, Cancer, NF-kappaB, Transport

## Abstract

The gut and local esophageal microbiome progressively shift from healthy commensal bacteria to inflammation-linked pathogenic bacteria in patients with gastroesophageal reflux disease, Barrett’s esophagus, and esophageal adenocarcinoma (EAC). However, mechanisms by which microbial communities and metabolites contribute to reflux-driven EAC remain incompletely understood and challenging to target. Herein, we utilized a rat reflux-induced EAC model to investigate targeting the gut microbiome–esophageal metabolome axis with cranberry proanthocyanidins (C-PAC) to inhibit EAC progression. Sprague-Dawley rats, with or without reflux induction, received water or C-PAC ad libitum (700 μg/rat/day) for 25 or 40 weeks. C-PAC exerted prebiotic activity abrogating reflux-induced dysbiosis and mitigating bile acid metabolism and transport, culminating in significant inhibition of EAC through TLR/NF-κB/TP53 signaling cascades. At the species level, C-PAC mitigated reflux-induced pathogenic bacteria (*Streptococcus parasanguinis*, *Escherichia coli*, and *Proteus mirabilis*). C-PAC specifically reversed reflux-induced bacterial, inflammatory, and immune-implicated proteins and genes, including *Ccl4*, *Cd14*, *Crp*, *Cxcl1*, *Il6*, *Il1b*, *Lbp*, *Lcn2*, *Myd88*, *Nfkb1*, *Tlr2*, and *Tlr4*, aligning with changes in human EAC progression, as confirmed through public databases. C-PAC is a safe, promising dietary constituent that may be utilized alone or potentially as an adjuvant to current therapies to prevent EAC progression through ameliorating reflux-induced dysbiosis, inflammation, and cellular damage.

## Abstract 2

Cranberry proanthocyanidins are promising dietary constituents for mitigating reflux-driven proinflammatory microbiota and their metabolites, through TLR/NF-&kappa;B/TP53 signaling cascades, culminating in esophageal adenocarcinoma inhibition.

## Graphical abstract

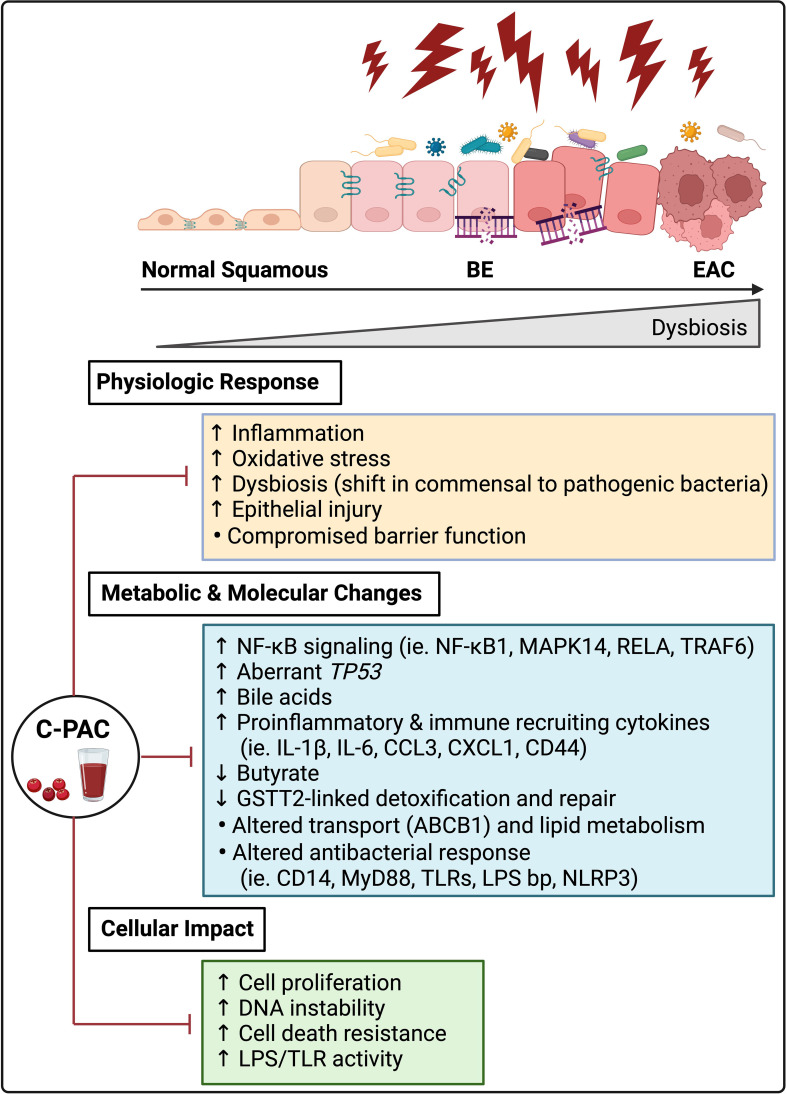

## Introduction

Esophageal adenocarcinoma (EAC) represents a growing health problem characterized by markedly increased incidence in the last 50 years, substantial morbidity, and high mortality (1). The only known precursor lesion to EAC is Barrett’s esophagus (BE), a metaplastic protective adaptation to chronic reflux of injurious bile and acidic gastric contents, known as gastroesophageal reflux disease (GERD) (1). Acid-reducing proton pump inhibitors (PPIs) are the mainstay of treatment for GERD, but the complete response rate is only approximately 50% (2, 3). In turn, widespread use of PPIs has not translated to meaningful declines in EAC or improved survival, and overall results across studies are inconsistent (4–6). The long-term safety of PPIs has also been questioned, with potential adverse events including reduced gut microbiome health and increased enteric infections (*Clostridium difficile*), deficiencies of micronutrients, and renal insufficiency (7–10). Microbial communities progressively shift from healthy commensal bacteria to inflammation-linked pathogenic bacteria (*Staphylococcus*, *Streptococcus*, *Enterococcus*, and *Escherichia*) in GERD, BE, and EAC (11–17). In the context of microbial dysbiosis, pathogenic bacteria are documented to produce DNA-damaging toxins, including lipopolysaccharide (LPS) and colibactin as well as proinflammatory metabolites with cancer-promoting effects (18–20). However, mechanisms by which changes in the gut microbiome–esophageal metabolome axis contribute to EAC progression remain poorly defined and in turn difficult to successfully target. Beyond dysbiosis, reflux components are well documented to induce DNA damage in esophageal cells and to stimulate cytokine-mediated inflammation, further contributing toward epithelial injury, immune cell migration, genetic instability, and cancer progression (18, 21). Repeated exposure to refluxate also leads to diminished defense mechanisms, as evidenced by esophageal barrier dysfunction and reduced levels of esophageal DNA repair enzymes, suggesting compromised repair capacity in reflux-exposed esophageal epithelium (18, 22, 23). Reflux-induced mucosal damage coupled with esophageal barrier dysfunction allows for increased interaction between pathogenic bacteria and epithelium, further promoting esophageal injury and risk for EAC progression. Thus, our goal is to identify a safe efficacious agent, for use alone or as an adjuvant, to inhibit reflux-induced EAC by targeting the major drivers and sequelae associated with GERD and BE. Prognosis following a diagnosis of EAC remains poor as evidenced by less than 20% 5-year survival (1), supporting the urgent need for improved preventive strategies.

Cranberry is a fruit rich in polyphenols, including proanthocyanidins (C-PAC) that exert potent antiinflammatory and antibacterial activities (24–28) in human trials (26–33) and anticancer effects in multiple preclinical models, including EAC xenografts (34–36). Historically, C-PAC was considered to have poor bioavailability and remained underexplored for anticancer effects, particularly in vivo. Recently, interest in C-PAC was renewed with increased understanding of absorption, digestion, and the salient role gut microbes play in generating biologically active metabolites (37, 38). Herein, we evaluated the cancer-inhibitory mechanisms of C-PAC by utilizing a translationally relevant rat model of reflux-induced EAC. Key findings show that C-PAC exerts prebiotic activity by stimulating the growth of beneficial bacteria and abrogating reflux-induced dysbiosis, as evidenced by increasing antiinflammatory and butyrate-producing bacteria (*Lactobacillus* and *Allobaculum*), while decreasing proinflammatory LPS-producing Gram-negative bacteria (*Escherichia coli*)*,* and mitigating reflux-induced bile acid (BA) metabolism and transport, culminating in inhibition of high-grade dysplasia (HGD) and EAC through inhibition of Toll-like receptor (TLR)/NF-κB/TP53 signaling. Importantly, the concentration of C-PAC required to exert biological activity is readily achievable as part of the normal diet.

## Results

### Dose-range-finding study and C-PAC safety.

Rats consuming C-PAC in the drinking water at 250, 500, and 700 μg/rat/day for 6 weeks experienced no adverse effects in terms of body weight, food, or water consumption compared to water controls (no significant differences based on repeated-measures ANOVA, Supplemental Figure 1A; supplemental material available online with this article; https://doi.org/10.1172/jci.insight.168112DS1). All organ pathology was normal and comprehensive serology measures (Supplemental Figure 1B) did not differ between rats consuming 700 μg/rat/day C-PAC and those receiving water alone.

### C-PAC inhibits progression of reflux-induced EAC.

**Figure 1:**
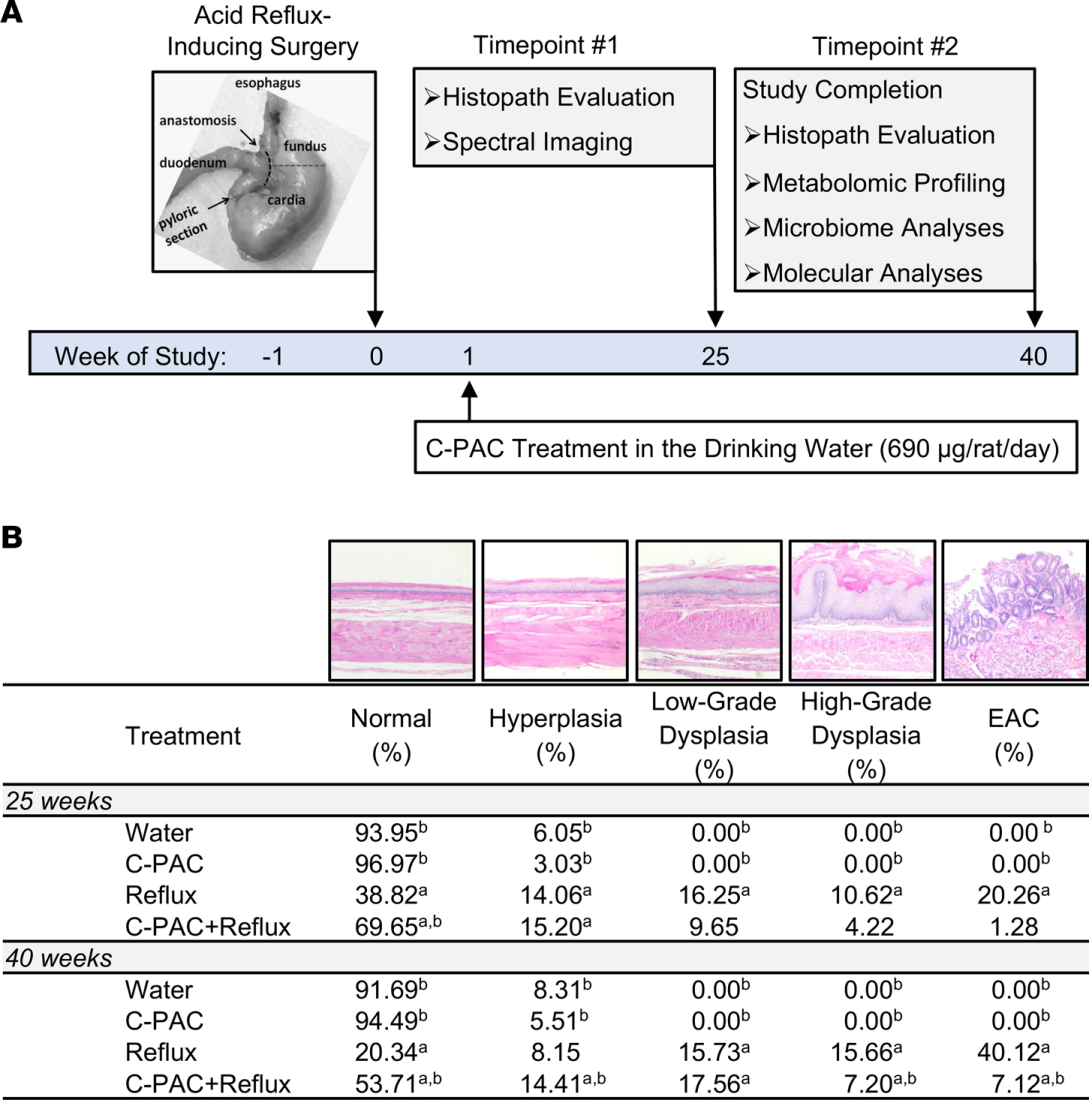
C-PAC inhibits cancer progression in a rat model of reflux-induced EAC. (**A**) At baseline (week 0), esophagogastroduodenal anastomosis was performed to induce acid reflux in designated treatment groups. One week later, animals were provided either water or C-PAC (690 μg/rat/day) ad libitum. At 25 weeks, animals were sacrificed to perform spectral imaging and characterize esophageal histopathology. At study completion (40 weeks), histopathological evaluations, esophageal metabolomics, gut microbiome profiling, and esophageal molecular analyses were conducted. (**B**) Histopathological evaluation of esophagi (*n* = 5–8 animals per treatment) was performed on weeks 25 and 40 by quantifying the percentage of normal, hyperplasia, low-grade dysplasia, high-grade dysplasia, and EAC across treatment groups. ^a^*P* ≤ 0.05 versus water treated. ^b^*P* ≤ 0.05 versus reflux treated. Data are presented as mean values per pathology and were analyzed by 2-sided Student’s *t* test. C-PAC, cranberry proanthocyanidins; EAC, esophageal adenocarcinoma.

Based on dose-range-finding study results, consideration of the published literature (32, 33), and behaviorally achievable consumption levels, C-PAC was evaluated at a concentration targeting 700 μg/rat/day in the long-term bioassay, resulting in actual average delivery of 690 μg/rat/day. As depicted in Figure 1A, following reflux-inducing surgery, animals received water or C-PAC ad libitum in the drinking water. Rats were sacrificed at 25 weeks for histopathological evaluation and spectral imaging (39). At the final 40-week time point, evaluations included histopathology, metabolomics, microbiome profiles, and molecular analyses. As shown in Figure 1B, reflux significantly induced HGD and progression to EAC, while reducing the percentage of normal-appearing esophageal fields over time. C-PAC effectively mitigated the deleterious impact of reflux, as evident by preservation of normal epithelial fields and inhibition of HGD and EAC, most evident at 40 weeks. Compared with reflux alone, the C-PAC+reflux group mitigated EAC formation by 93.68% and 82.25% at 25 and 40 weeks, respectively. C-PAC reduced HGD by approximately 60% at both time points. At 25 and 40 weeks, C-PAC+reflux significantly maintained fields of normal epithelium (69.65% and 53.71%), relative to reflux alone (38.82% and 20.34%), supporting the notion that C-PAC potently mitigates refluxant, subsequent tissue damage, and progression to EAC.

C-PAC delivered in the drinking water was safe over the 40-week study duration based on mean body weight, weight gain, and food and water consumption, which did not differ between the reflux and C-PAC+reflux groups (Supplemental Figure 2, A–D, and Supplemental Tables 1–4). Body weight differences did emerge between reflux and nonreflux groups due to weight loss during the surgical week, from which the rodents never fully rebounded. Importantly, all groups continued to gain weight normally, allowing for valid comparisons across treatments.

### C-PAC mitigates reflux-driven proinflammatory gut microbiome changes.

**Figure 2:**
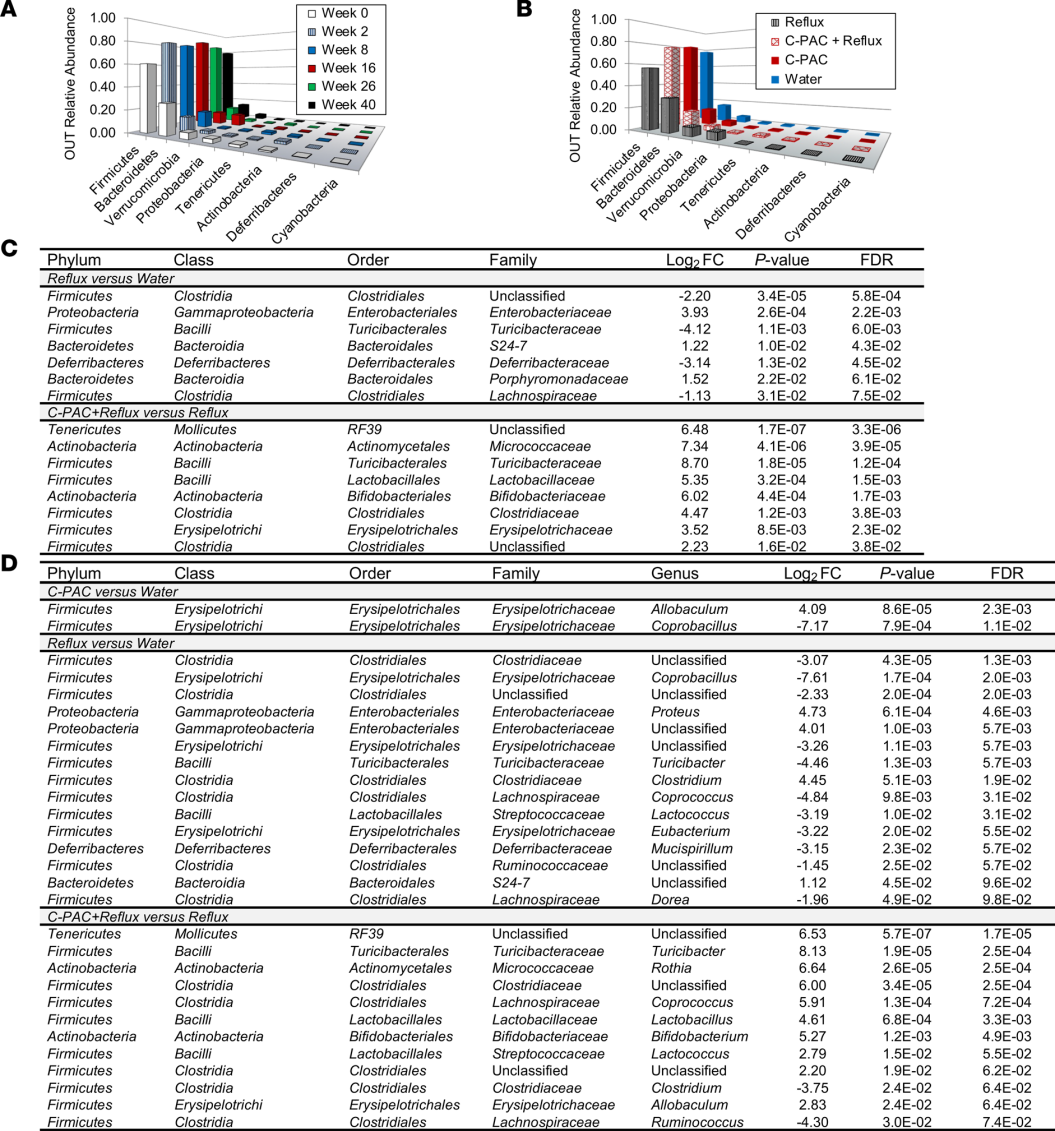
C-PAC ameliorates reflux-induced proinflammatory gut microbiome changes. (**A**) Gut microbiome profile of water-treated animals from baseline through 40 weeks. Phylum-level analysis of 16S rRNA gene sequencing shows stabilization of microbiome profiles after 2 weeks. (**B**) Phylum-level gut microbiome profiles for all treatment groups at 40 weeks. (**C**) Family-level changes in the microbiome for each treatment group at 40 weeks. (**D**) Genus-level changes in the microbiome across treatment groups (40 weeks). Data are presented as log_2_FC in relative abundance levels, with *P* ≤ 0.05 (Robinson and Smyth’s exact test) and FDR ≤ 0.1 (Benjamini-Hochberg correction). C-PAC, cranberry proanthocyanidins; FC, fold change.

To evaluate C-PAC’s capacity to modulate reflux-induced changes in the gut microbiome, we performed 16S rRNA gene sequencing of fecal pellets collected from water-treated rats over time and from all treatment groups at week 40. There was reasonable separation of animals by treatment group based on hierarchical clustering of fecal microbiome samples with the reflux group clustered together, the water and C-PAC treated group intermingled, and the C-PAC+reflux group clustered with either the reflux or nonreflux groups (Supplemental Figure 3A). There were no differences in species richness or α diversity, yet statistically significant differences were observed in β diversity, supporting differences in microbial communities in reflux compared with C-PAC+reflux groups (Supplemental Figure 3). Consistent with the published literature (40), Figure 2A shows that the rat fecal microbiome stabilized after 2 weeks and the normal microbiome was dominated by the phylum *Firmicutes* followed by *Bacteroidetes* and *Verrucomicrobia*, a composition mimicking normal healthy human esophageal microbiome profiles (11–14). At 40 weeks of study, reflux significantly reduced phylum abundance levels of *Firmicutes* and significantly induced proinflammatory LPS-linked Gram-negative *Proteobacteria*, *Deferribacteres*, and *Bacteroidetes* (Figure 2B) (12, 14, 18). In contrast, C-PAC treatment in the context of reflux significantly increased phylum abundance levels of Gram-positive *Firmicutes*, *Tenericutes*, and *Actinobacteria*, with significantly reduced *Proteobacteria* as well as reduced abundance of *Bacteroidetes* and *Verrucomicrobia* (Figure 2B).

At the family and genus levels, C-PAC in the context of reflux shifted the gut microbiome toward health-associated Gram-positive bacterial populations, increasing beneficial *Firmicutes* and *Actinobacteria* members including *Streptococcaceae*, *Lactobacillaceae*, and *Bifidobacteriaceae*, while reducing levels of deleterious *Clostridium* members (Figure 2, C and D) (11). Levels of *Bifidobacteriaceae* were also significantly increased by C-PAC (*P* = 0.021, FDR = 0.17). At the genus level, C-PAC alone increased levels of *Allobaculum* and *Coprobacillus* (Figure 2D). Conversely, family and genus levels of Gram-negative *Enterobacteriaceae* and *Proteus* were significantly increased with reflux.

Next, we evaluated species-level frequency changes utilizing the Axiom microbiome array platform. Results (Supplemental Table 5) revealed that reflux increased pathogenic bacteria, including *Odoribacter laneus*, *Streptococcus mutans*, *Streptococcus parasanguinis*, *Proteus mirabilis*, *E*. *coli*, *Enterococcus faecalis*, *Clostridium perfringens*, and *Lactobacillus* sp. ASF360, and reduced acid-resistant antiinflammatory *Lactobacillus johnsonii* (41). C-PAC mitigated many reflux-induced bacterial species-level changes. C-PAC also decreased specific viral families in the context of reflux, including *Myoviridae* (*P* = 0.0065) and *Siphoviridae* (*P* = 0.0549) (Supplemental Table 6).

### Esophageal metabolomics reveals anticancer activities of C-PAC.

**Figure 3:**
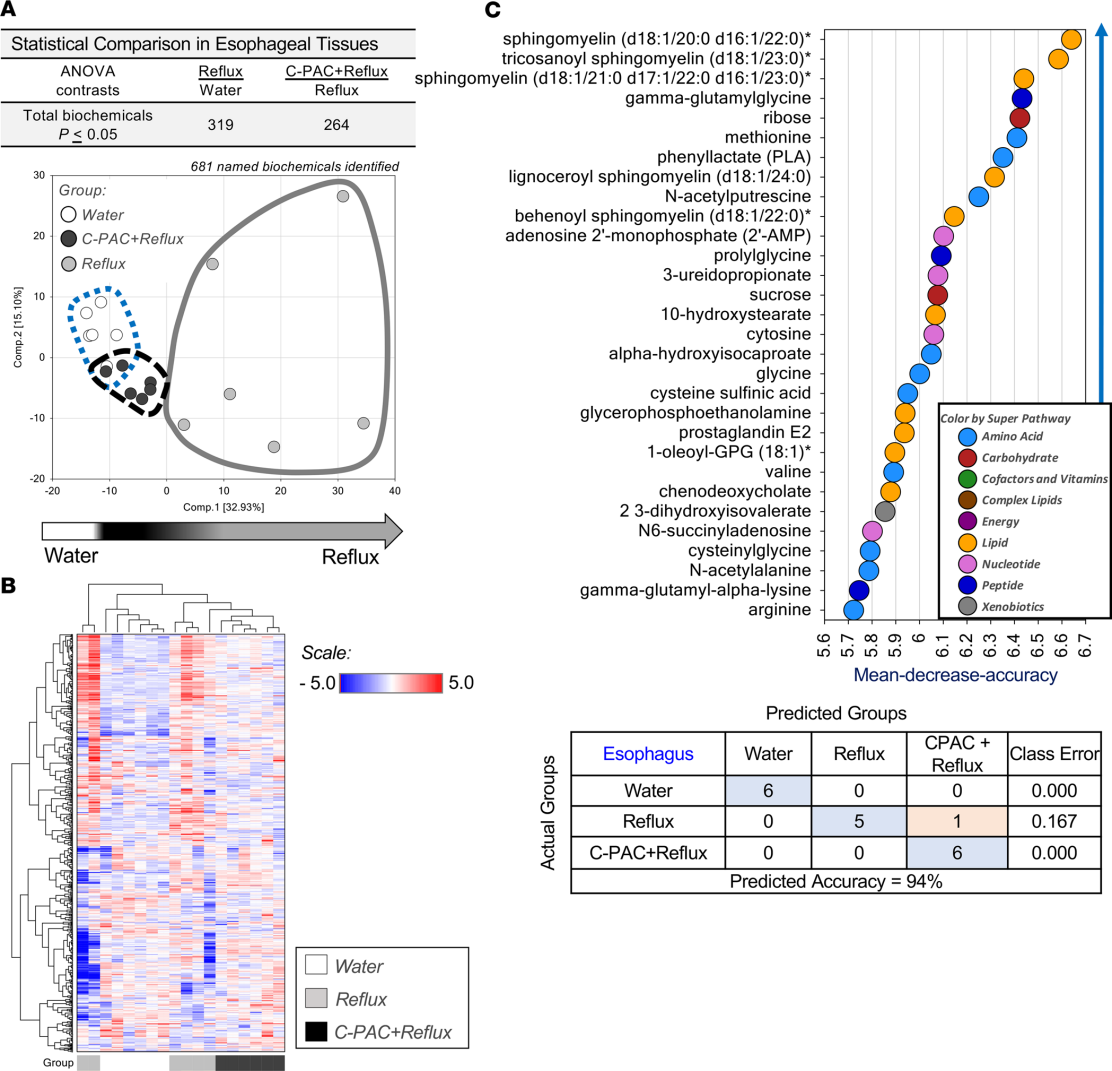
Untargeted metabolomic profiling of esophagi following treatment with C-PAC in reflux-induced EAC. (**A**) Untargeted metabolomics (*n* = 6 animals per treatment group) revealed 681 named biochemicals, with 319 and 264 significantly altered with reflux or C-PAC in the context of reflux, respectively. Principal component analysis supported variation between individual samples and treatment groups. (**B**) Hierarchical clustering of samples showed strong clustering based on treatment. (**C**) Biochemical importance plot results identified specific metabolites in superpathways of increasing importance, with random forest classification analysis providing a predictive accuracy of 94%. One-way ANOVA identified significant biochemicals (*P* ≤ 0.05). C-PAC, cranberry proanthocyanidins; EAC, esophageal adenocarcinoma.

To characterize metabolic effects of C-PAC in vivo, we conducted untargeted metabolomics on esophageal tissues across treatment groups. A total of 681 compounds were identified, with 319 metabolites significantly different in reflux and 264 metabolites significantly different in C-PAC+reflux (Figure 3A). The principal component analysis (PCA) plot in Figure 3A shows that esophageal samples separate into well-defined groups by treatment, with the greatest variability present with reflux induction. Hierarchical clustering of esophageal samples further supports strong clustering by treatment group (Figure 3B). Next, a biochemical importance plot (Figure 3C) was generated through random forest analysis, revealing superpathways and specific metabolites differentiating between treatments, with a predictive accuracy of 94%. Top identified superpathways were dominated by amino acids and lipids. Specific biochemicals were linked to redox homeostasis, bacterial pathogenesis, and inflammation, including γ-glutamyl amino acids, lysine, methionine, cytosine, cysteine, glycine, as well as numerous sphingomyelins, the proinflammatory lipid prostaglandin E2, and the primary BA chenodeoxycholate (Figure 3C).

**Figure 4:**
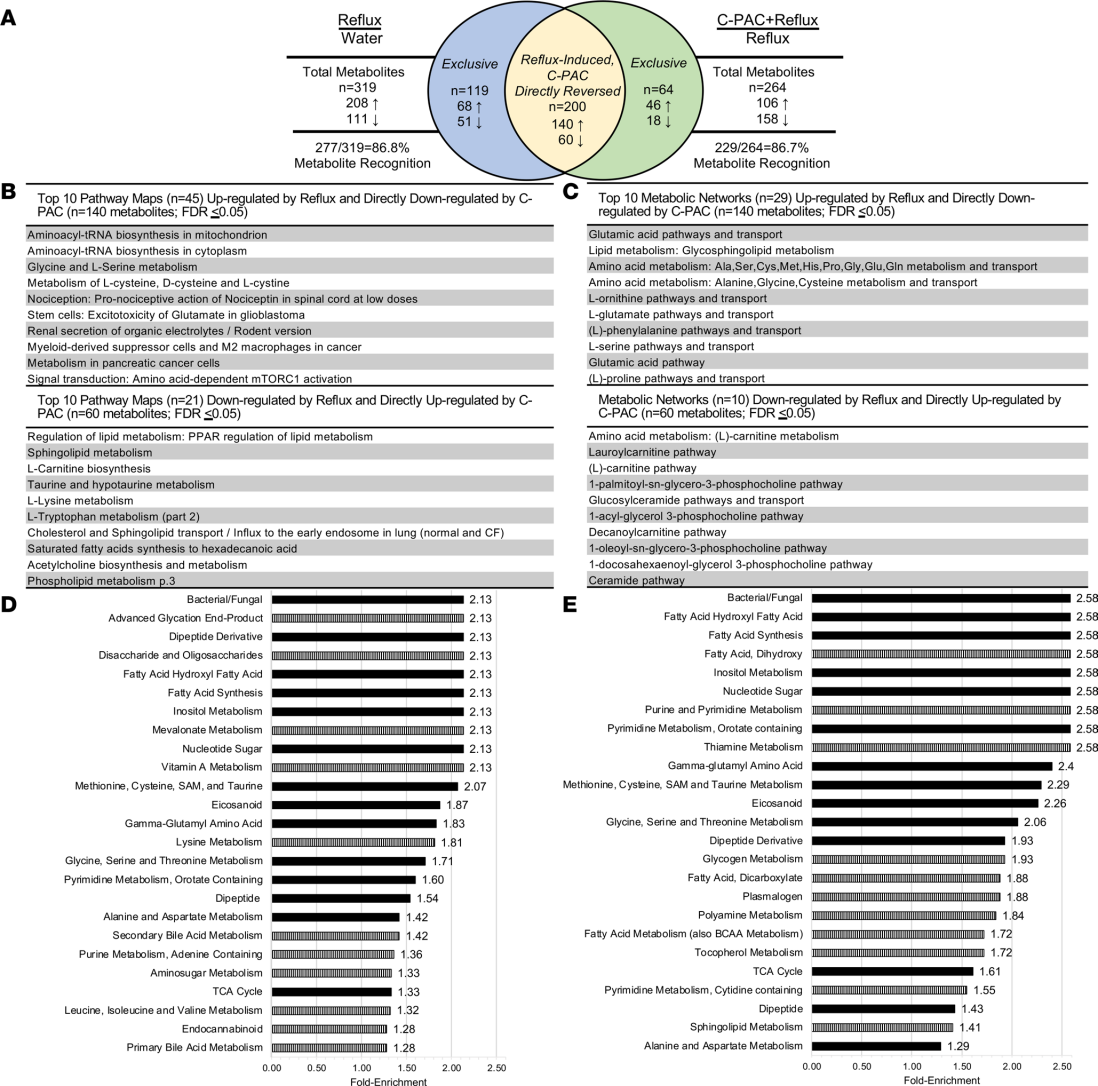
C-PAC mitigates reflux-induced esophageal metabolite dysregulation. (**A**) Venn diagram depicting significant (*P* ≤ 0.05) metabolites in reflux versus water (*n* = 319) and C-PAC+reflux versus reflux (*n* = 264) groups. The blue and green sections represent metabolites exclusive to reflux versus water (*n* = 119) and C-PAC+reflux versus reflux (*n* = 64), respectively. The yellow section represents metabolites induced by reflux and directly reversed by C-PAC (*n* = 200). Metabolite directionality is noted as upregulated (↑) or downregulated (↓). (**B**) The top 10 pathway maps for metabolites induced by reflux and directly reversed by C-PAC in the context of reflux. Supplemental Tables 9 and 10 provide complete lists of significant pathway maps. (**C**) The top 10 metabolic networks induced by reflux and directly reversed by C-PAC in the context of reflux. Supplemental Tables 11 and 12 provide complete results of significant metabolic networks detected. Pathway set enrichment analysis performed in Metabolync for (**D**) reflux versus water and (**E**) C-PAC+reflux versus reflux, where black bars are shared between comparisons and vertical striped bars are exclusive to the comparison in each panel. Significant pathway maps and metabolic networks are based on *P* ≤ 0.05 and FDR ≤ 0.05. C-PAC, cranberry proanthocyanidins.

Next, significantly modulated metabolites were evaluated based on treatment group comparisons. The Venn diagram in Figure 4A shows that of the 319 metabolites significantly altered by reflux, C-PAC directly reversed 62.7% (200 metabolites). Top identified pathway maps and metabolic networks derived from the 200 C-PAC–mitigated metabolites are summarized in Figure 4, B and C, and Supplemental Tables 7–10. These results align closely with the biochemical importance plot results, supporting the idea that reflux upregulated amino acid metabolism as well as aminoacyl-tRNA biosynthesis and myeloid-derived suppressor and M2 macrophages, all of which are directly reversed by C-PAC. Similarly, metabolic network analysis (Figure 4C) revealed that C-PAC reversed reflux-induced amino acid metabolism and transport as it relates to glutamate, serine, and proline, as well as glycosphingolipid metabolism. Metabolic networks, including carnitine, ceramide, and phosphocholine pathways, were significantly downregulated by reflux and directly reversed by C-PAC.

C-PAC mitigated upregulated reflux-induced process networks of translation and vesicle transport (Supplemental Table 11). Interestingly, leptin signaling and BA transport were significantly downregulated by reflux and directly reversed by C-PAC (Supplemental Table 12). Finally, pathway set enrichment analysis via Metabolync identified the top enriched pathways by treatment group, as shown in Figure 4, D and E. With reflux, 47 pathways were significantly enriched with bacterial/fungal, advanced glycation end-product, and dipeptide derivative as the top pathways. The top significantly enriched pathway in C-PAC+reflux was also bacterial/fungal, supporting mitigation of this reflux-induced pathway by C-PAC. Additional pathways modulated by C-PAC in the context of reflux include those associated with fatty acids, eicosanoids, amino acid and nucleotide metabolism, γ-glutamyl amino acids, and the TCA cycle.

### C-PAC mitigates reflux-induced changes in primary and secondary BAs.

**Figure 5:**
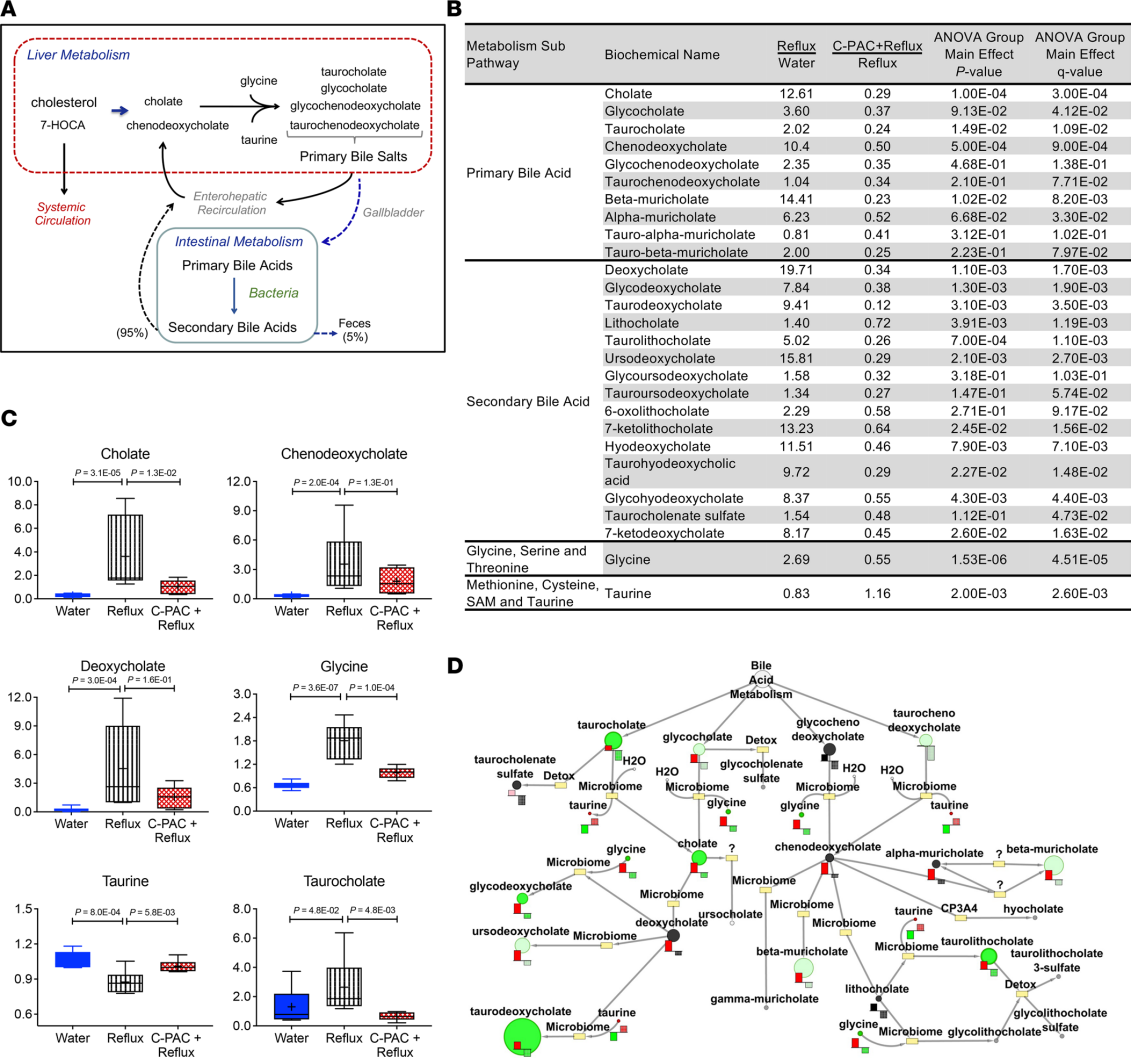
C-PAC mitigates reflux-driven alterations in esophageal bile acids and conjugating amino acids. (**A**) Summary diagram of BA production and processing. BA metabolism includes phase I BA synthesis in the liver generating relatively equal amounts of the 2 major primary BAs cholate and chenodeoxycholate from cholesterol in humans and in alignment with the rat EAC model. Next, BAs undergo phase II conjugation to glycine or taurine in preparation for phase III transport, deconjugation, and subsequent conversion by bacteria to highly insoluble and toxic secondary BAs. During gastrointestinal transit most BAs reenter enterohepatic recirculation, leaving only about 5% of BAs to pass through the colon and be excreted in the feces. (**B**) Primary and secondary BAs, as well as conjugating amino acids with relative fold change levels in reflux versus water or C-PAC+reflux versus reflux comparisons. Group main effects were determined by 1-way ANOVA with Storey’s correction for FDR (*q* value). (**C**) Representative box-and-whisker plots for select metabolites. Data are presented as maximal and minimal distribution values (whiskers), the limits of the upper (75th) and lower (25th) quartiles (box), group median values (line), and group mean values (+): water (blue), reflux (white with vertical black stripes), and C-PAC+reflux (red mesh). *P* values are based on 1-way ANOVA. (**D**) BA metabolism pathway generated in Metabolync displaying significantly dysregulated BAs (*P* ≤ 0.05), with circle size indicating relative metabolite level. Levels in bar-and-whisker plots for reflux versus water shown in red and C-PAC+reflux versus reflux shown in green. C-PAC, cranberry proanthocyanidins; BA, bile acid.

Esophageal tissue BAs were assessed, considering that reflux of bile and acidic stomach contents has a well-documented role in GERD, BE, and EAC progression (1). Fundamentally, BA metabolism includes phase I BA synthesis generating relatively equal amounts of the 2 major primary BAs, cholate and chenodeoxycholate, in humans and in alignment with our rat model data (42). Next, BAs undergo phase II conjugation to glycine or taurine (Figure 5A), which occurs in a 3:1 ratio in humans and again similarly in rats (42). Conjugation prepares BAs for phase III transport, deconjugation, and subsequent conversion by bacteria to highly insoluble and toxic secondary BAs (43). Metabolic profiling revealed that reflux significantly increased levels of multiple primary BAs in rat esophagi, including cholate (12.6-fold), chenodeoxycholate (10.3-fold), and their derivatives, with C-PAC mitigating BA induction (Figure 5B). C-PAC also diminished reflux-induced changes in the conjugating amino acids, glycine and taurine. Box-and-whisker plots of select primary and secondary BAs, as well as BA metabolites, are shown in Figure 5C. Bacteria have a well-defined role in transformation of primary BAs into secondary BAs (Figure 5D), including deoxycholate and additional injurious secondary BAs that are upregulated with reflux and mitigated by C-PAC. Additional quantitative analysis of esophageal cholate and taurocholate confirmed induction by reflux (158.5 nM and 240.8 nM, respectively) and mitigation by C-PAC (100.0 nM and 73.7 nM, respectively). Parallel metabolomic analysis of liver and fecal samples revealed limited reflux-induced changes in BA profiles (data not shown).

Briefly, in the stool, reflux induction resulted in only 1 BA significantly changed (*P* ≤ 0.05) from water controls, 3β-hydroxy-5-cholenoic acid. It significantly declined with reflux (0.36-fold), and C-PAC treatment nonsignificantly increased levels 1.29-fold. In the liver, reflux significantly increased levels of taurobetamuricholate (2.88-fold) and taurodeoxycholate (4.88-fold) compared with water treatment, with nonsignificant mitigation by C-PAC (0.48-fold and 0.37-fold, respectively). It is also possible that the differences noted in the esophagus, although statistically significant, were below levels that disrupt the normal patterns of reabsorption and intestine-to-liver homeostasis. Further support for these results comes from the fact that watery stools or fatty livers were not observed among any of the treatment groups.

### C-PAC modulates a comprehensive battery of reflux-induced bacterial, inflammatory, and immune-related genes and proteins modified in human EAC.

To further investigate mechanisms by which C-PAC inhibits EAC in vivo, esophageal gene expression analysis was performed targeting a panel of bacterial, inflammatory, and immune-related genes (Table 1). In total, 47 genes were significantly dysregulated by either reflux or C-PAC+reflux. Reflux upregulated markers associated with proinflammatory Gram-negative bacteria (*Cd14*, *Myd88*, *Tlr2*, *Tlr4*, *Tlr9*, *Lbp*, *Nlrp1a*, and *Nlrp3*), activation of NF-κB signaling (*Bcl10*, *Nfkb1*, *Nfkbia*, *Mapk14*, *Rela*, and *Traf6*), and immune recruiting and proinflammatory cytokines linked to BE progression (*Il1b*, *Il6*, *Il18*, *Ccl3*, *Ccl4*, *Ccl5*, and *Cxcl1*), with C-PAC mitigating identified changes.

To better understand the translational relevance of our results, we queried 2 human data sets for expression changes between histologically normal human esophageal tissues and EAC (GEO GSE26886), as well as BE tissues with low-grade dysplasia (LGD) versus BE with HGD and EAC (GEO GSE193946). Of the 47 genes significantly dysregulated in the rat reflux-induced EAC model, 22 markers (47%) were significantly changed and moving in the same direction as detected in humans (Table 1).

**Figure 6:**
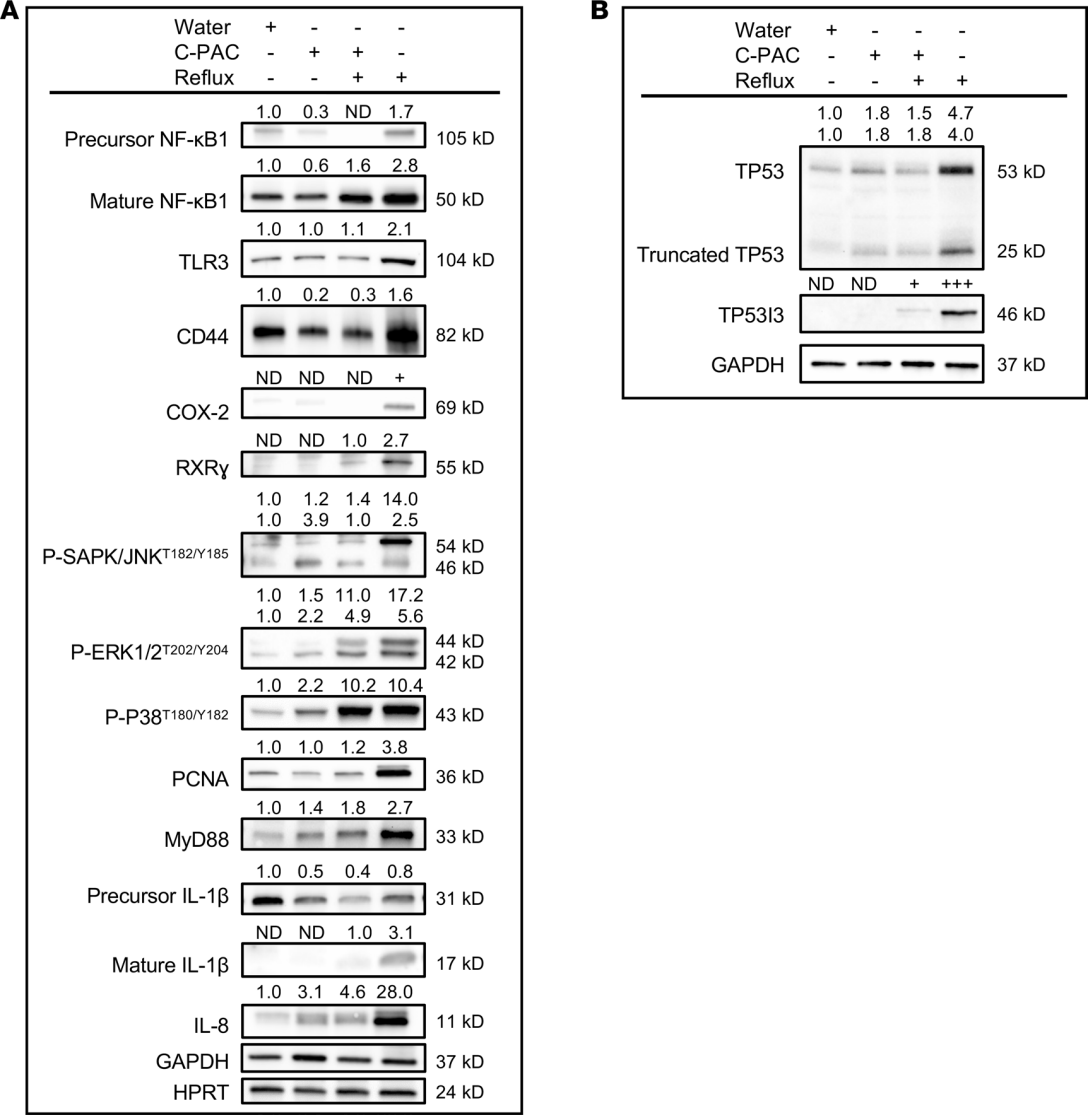
C-PAC mitigates bacterial, inflammatory, and immune-related markers dysregulated by reflux. At 40 weeks of study, Western blot analysis of esophageal lysates was performed using commercially available antibodies against (**A**) bacterial, inflammatory, and immune-linked proteins and (**B**) TP53-related proteins. The plus (+) symbol denotes treatment group. Expression values were normalized to GAPDH or HPRT as loading controls, and fold change from water calculated using ImageLab. Bands without detectable expression are denoted as ND. C-PAC, cranberry proanthocyanidins.

In addition to mitigating reflux-induced changes, C-PAC significantly altered expression of antimicrobial markers in normal, non–reflux-exposed esophagi (Supplemental Table 13), suggesting C-PAC exerts an early protective role preceding bile insult or altered pathology. In normal esophagi, C-PAC altered expression of numerous markers also identified as dysregulated in reflux (Table 1), including *Aps*, *Bcl10*, *Camp*, *Casp1*, *Ccl4*, *Ccl5*, *Cxcl1*, *Il6*, *Il12a*, *Irf7*, *Nfkbia*, *Prdx2*, *Sugt1*, and *Ticam2*. Five markers were uniquely altered by C-PAC in normal esophagi: *Ifnb1*, *Lyz2*, *Nod2*, *Prtn3*, and *Pycard*. In alignment with gene expression results, multiple bacteria- and inflammation-linked proteins were induced by reflux and mitigated by C-PAC (Figure 6). Increased levels of NF-κB1, TLR3, CD44, COX-2, MyD88, IL-1β, and IL-8 were observed with reflux and downregulated by C-PAC. Consistent with mutations in *TP53* arising with progression to human BE and EAC (44), reflux increased aberrant TP53 levels, with mitigation by C-PAC (45). Whole-genome sequencing (WGS) of rat esophagi across treatment groups identified 2,065 high- or moderate-level gene mutations induced by reflux, with C-PAC treatment directly reversing 774 (37.5%) of the mutations (data not shown). With direct relevance to mechanisms affecting *Tp53* function, reflux induced a high-impact stop-gain mutation in tumor protein *Tp53*-inducible protein 3 (*Tp53i3*) that was restored by C-PAC. *TP53I3* has quinone oxidoreductase activity and is involved in oxidative stress, DNA damage response, and *TP53*-mediated apoptosis (46, 47). Western blotting results showed strong induction of TP53I3 with reflux and mitigation by C-PAC at the protein level. These findings align with our previous findings showing that C-PAC mitigates reflux-induced esophageal DNA damage, as indicated by reduced levels of p-γH2AX (23). Thus, data shown in Figure 6B seem consistent with an oncogenic role for TP53I3 in this model system, as has been reported in papillary thyroid cancer via the regulation of the PI3K/AKT/PTEN pathway (47). Multiple other *p53*-interacting genes (i.e., *Tp53bp1*, *Tp53ill*, and *Tp53inp2*) were mutated in the reflux group as modifiers or noncoding variants or variants affecting noncoding genes, where predictions are difficult or unknown for discerning impact. We were not successful in locating antibodies to these gene “modifiers” that exhibited convincing specificity in the rat and therefore cannot draw any conclusions about their potential influence on TP53.

Reflux increased MAPK signaling through phosphorylation of SAPK/JNK^T182/Y185^, ERK1/2^T202/Y204^, and P38^T180/Y182^, while C-PAC mitigated MAPK signaling through reduced phosphorylation of SAPK/JNK and ERK1/2. Finally, consistent with human studies (48), increased levels of cell replication (PCNA) and retinoic acid receptor signaling (RXRγ) were observed with reflux and mitigated by C-PAC. Nuclear receptors, including retinoid family members, regulate host-microbiome crosstalk (49).

### Integration of metabolites and gene expression data reveals networks significantly altered by reflux and mitigated by C-PAC.

Given the changes in microbiome profiles, bacterial metabolites, and inflammatory signaling cascades with reflux, we utilized Metacore integration to investigate metabolite-gene networks based on significantly dysregulated metabolites in reflux versus water (*n* = 319) and C-PAC+reflux versus reflux (*n* = 264), coupled with significantly altered antibacterial genes (*n* = 47). Of note, Network 2 focuses on BA signaling through taurocholic acid, Networks 4–6 and 8–10 describe NF*-*κB signaling, whereas Networks 4 and 7 highlight signaling through TLR2, -4, and -9 (Supplemental Table 14), further supporting a strong role for microbiota in bile metabolism and inflammation. Networks 2 and 5 pictorially depict linkages between metabolite and gene signaling, including the BAs taurodeoxycholate and glycodeoxycholate, as well as the c-Myc/STAT3/IL-6 central signaling node, respectively (Supplemental Figure 4).

### Functional microbiome predictions using PICRUSt highlight the importance of transport, secondary metabolism, biofilms, and lipid biosynthesis for C-PAC’s anticancer activity in vivo.

**Figure 7:**
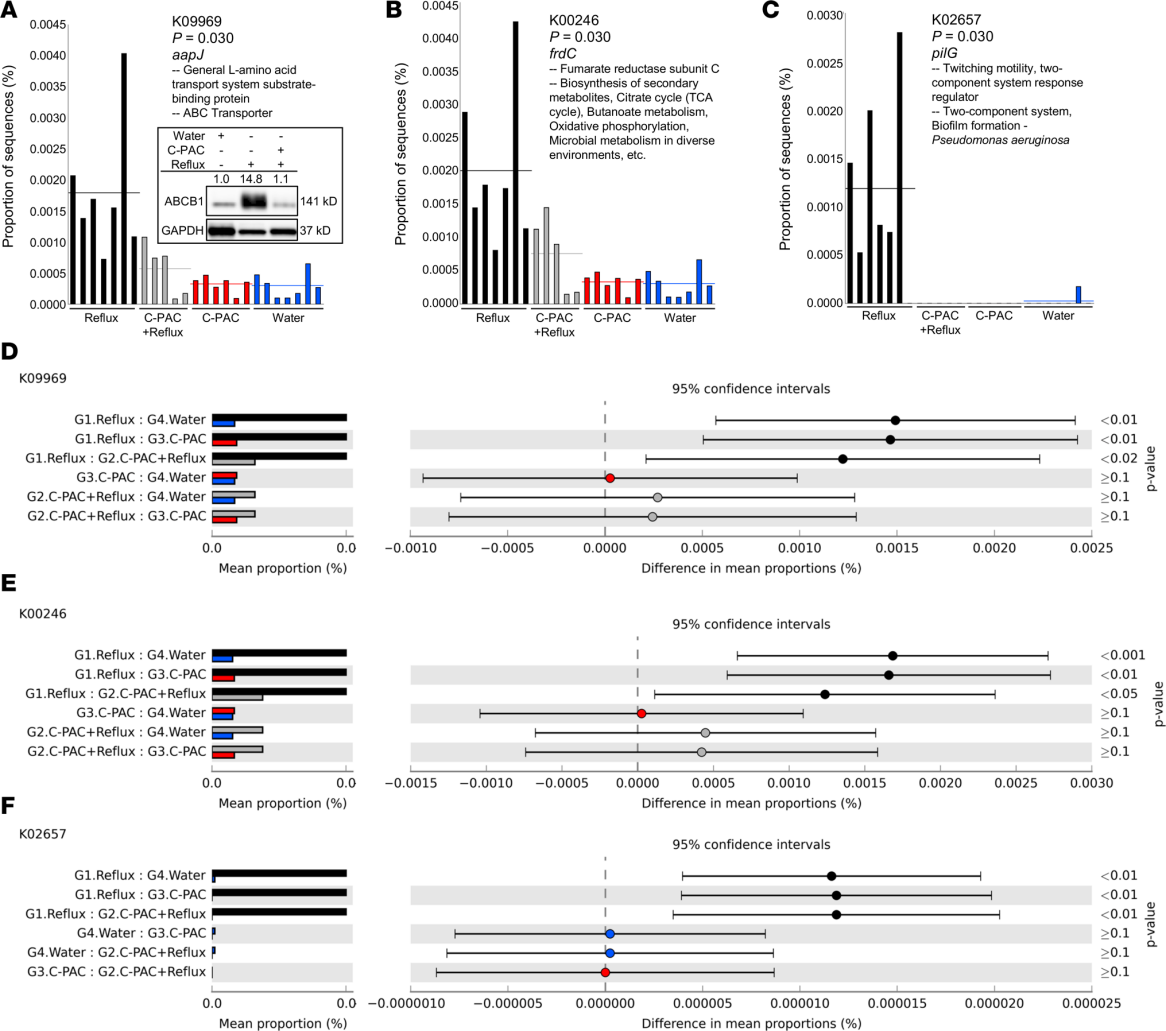
Functional microbiome predictions via PICRUSt reveal roles for C-PAC in vivo. 16S rRNA gene sequencing of fecal pellets from animals at 40 weeks was analyzed using PICRUSt and visualized in Statistical Analysis of Metagenomic Profiles (STAMP). (**A**–**C**) Highlighted results by animal and treatment assignment and (**D**–**F**) group-wise comparisons based on treatment are shown; (**A** and **D**) *aajP*, a general L-amino acid ABC transporter; (**B** and **E**) *frdC*, fumarate reductase subunit C; and (**C** and **F**) *pilG*, twitching motility gene. Treatment groups are denoted by color: reflux (black), C-PAC+reflux (gray), C-PAC (red), and water (blue). Data were analyzed by 1-way ANOVA with Tukey’s post hoc test and Storey’s FDR correction. (**A**) Western blot inset of bile acid ABC transporter, ABCB1, in rat esophageal tissues. ABCB1 protein levels were normalized to GAPDH and fold change from water was calculated. C-PAC, cranberry proanthocyanidins.

To infer functional consequences of gut microbiome changes in reflux-induced EAC and mitigation by C-PAC, we utilized phylogenetic investigation of communities by reconstruction of unobserved states (PICRUSt). KEGG Orthology (KO) and pathways were characterized at classification levels 2–3 (Table 2). Predicted significant (*P* < 0.05, FDR < 0.05) functional pathways at level 2 are related to metabolism, specifically glycan and lipid metabolism. Level 3 predicted functional pathways significantly changed across treatments (ANOVA *P* ≤ 0.05), including biosynthesis of ansamycins, bacterial invasion of epithelial cells, pathogenic *E*. *coli* infection, ABC transporters, and glutathione metabolism in alignment with other results herein. Functional analysis (Figure 7, A–F, and Supplemental Table 15) identified 125 KOs significantly modulated.

Multiple bacterial genes linked to transport, including *aapJ*, *uraA*, *tolA*, *tolR*, *lptE*, *phaA*, *phaC-G*, and *bamA* (Figure 7, A and E) were significantly upregulated with reflux and reversed by C-PAC. Consistent with *aapJ* belonging to the bacterial family of ABC transporters and being upregulated with reflux, we observed increases in the BA transporter ABCB1 and potent mitigation by C-PAC at the protein level (Figure 7A). Additionally, multiple genes with roles in bacterial metabolism were significantly dysregulated with reflux and restored by C-PAC (Figure 7, B and F), including *frdC*, *glpK*, *mlycD*, *fumB*, *pccA*, and *ligA*, which are linked to biosynthesis of secondary metabolites, tricarboxylic acid cycle (TCA), butanoate metabolism, oxidative phosphorylation, and propanoate metabolism (Supplemental Table 15). Bacterial twitching and biofilm formation occur through *pilG*, which was upregulated with reflux and ablated with C-PAC (Figure 7, C and F). Lastly, enzymes linked to lipid biosynthesis, including *fadK*, and peptidoglycan biosynthesis as it relates to glycan biosynthesis and metabolism (*ampD*, *mltA*, and *pbpG*) were upregulated with reflux and downregulated by C-PAC, consistent with observed changes in the gut microbiome and bacterial metabolites.

## Discussion

Our laboratory has previously shown the cancer inhibitory potential of C-PAC utilizing human esophageal normal, BE, and EAC cell lines and OE19 EAC tumor xenografts (22, 34–36, 50). The current study extends evaluation of C-PAC to a more translationally relevant reflux-induced EAC model. Importantly, this rat model permits assessment of C-PAC in the context of the main risk factor driving the development of BE and EAC progression (1), namely reflux of bile and acidic gastric contents into the lower esophagus. Oral delivery of C-PAC (690 μg/rat/day) was well tolerated throughout the chemoprevention bioassay and resulted in potent anticancer effects when ingested at behaviorally achievable levels (human equivalent of approximately 39 mg/day based on standard allometric scaling conversion methods from the rat). The totality of published literature on C-PAC in human populations support efficacy targeting microbial and metabolic outcomes at 36 to 211 mg C-PAC/day and when ingested twice a day due to C-PAC’s pharmacokinetics (24–28). Thus, bioactive concentrations of C-PAC (60–80 mg) can be achieved in humans by consuming 2–4 ounces of 100% cranberry juice, 8–10 ounces of a 27% cranberry juice cocktail, or approximately one-quarter cup fresh cranberries (24–28). While we and others have shown the cancer inhibitory potential of C-PAC in multiple in vitro models (36), we believe the current study is the first to show that C-PAC inhibits reflux-driven EAC in vivo and to decipher mechanisms of inhibition linked to reflux-driven dysbiosis, transport, and metabolism of bile, and TLR/NF-κB/TP53 signaling.

The role of the gut microbiota influencing systemic inflammation is well documented, but less is known regarding responses at remote tissue sites, including the esophagus. Emerging evidence supports the notion that changes in microbiota and metabolites are linked to GERD, BE, and EAC progression (11–14). The microbiome among these patients is increasingly dominated by Gram-negative LPS-producing pathogenic bacteria (12–16), as is the microbiome in the rat reflux-induced EAC model. Many proinflammatory family- and species-level bacteria (i.e., *Enterobacteriaceae*, *Verrucomicrobiaceae*, and *Proteus* spp.) are increased in BE and EAC patients (13) and similarly induced in the rat reflux model, with significant mitigation by C-PAC. Moreover, *S*. *mutans* and *S*. *parasanguinis* are linked to progression from BE to EAC and both are induced by reflux and mitigated by C-PAC in the rat stool (11–17). Overall, C-PAC restored reflux-induced gut microbiota changes to a more healthful, antiinflammatory state with increased levels of Gram-positive *Firmicutes* phylum members. C-PAC increased abundance of health-associated *Lactococcus*, *Lactobacillus*, and *Bifidobacterium*, bacteria frequently administered as probiotics (51). Furthermore, *Allobaculum*, a Gram-positive butyrate-producing bacterium (52), was significantly increased by C-PAC alone. Additionally, 2 members of the gut virome, *Myoviridae* and *Siphoviridae*, increased with progression from BE to EAC (53), and we report that C-PAC decreased the frequency of these viral families in the context of reflux. Virome-level changes were postulated to be linked to LPS-induced alterations in the gut microbiome composition resulting in inflammation and EAC progression (53). Thus, modulation of the gut microbiome and virome by C-PAC is consistent with abrogating reflux-induced inflammation-linked microbial pathogenesis and its contribution to EAC progression. To date, few human studies utilizing cranberries have been completed that profile the microbiome. However, 2 trials support the idea that cranberries exert beneficial effects on the microbiome. Two weeks of a single daily serving of sweetened dried cranberries increased commensal gut bacteria and decreased pathogenic bacteria in healthy individuals (29). Similarly, a short-duration randomized controlled trial of 11 healthy volunteers fed an animal-based diet linked to dysbiosis reported that cranberry powder attenuated effects by increasing *Firmicutes* and short-chain fatty acids, while decreasing *Bacteroidetes* and, interestingly, reducing deoxycholic and lithocholic secondary BAs, both implicated in reflux (28). These studies provide direct evidence that dietary cranberry products favorably influence the gut or fecal microbiome and microbial metabolites in healthy humans, yet studies in patients with altered esophageal pathologies are nonexistent.

Considering that microbiota exert procarcinogenic effects by influencing host metabolism (18–20), we conducted untargeted metabolomics across treatment groups revealing alterations in microbial, immune-, and inflammation-linked esophageal metabolites, with reflux and mitigation by C-PAC. Most notable is the influence of C-PAC on reflux-induced BAs, the main exposure contributing to EAC progression (1). C-PAC diminished levels of both primary and bacteria-derived secondary BAs in reflux-exposed esophagi. Favorable metabolic findings were further supported by Pathway map, Metabolic network, and enrichment analyses revealing that reflux changes bacterial/fungal, fatty acid synthesis, methionine/cysteine/SAM/taurine, γ-glutamyl amino acid, TCA cycle, and eicosanoid metabolism, with C-PAC reversing these proinflammatory cancer-linked changes. Additional specific metabolites upregulated by reflux and mitigated by C-PAC included glycine, glutamate, glutamine, and lysine, all metabolites identified in esophageal tissues and serum from EAC patients (54, 55).

Process network and functional microbiome prediction using PICRUSt provided additional mechanistic insight regarding C-PAC’s prebiotic cancer inhibitory activity. Several transporter KOs were significantly upregulated by reflux and downregulated by C-PAC, including the ABC transporter K09969 and the BA efflux pump *ABCB1*, also known as multidrug resistance 1 (*MDR1*) due to its role in transport of anticancer agents (56). In accord with this, ABC transporters are enriched in HGD and EAC samples compared with nondysplastic BE and LGD human esophageal tissues (13). Additional transporters are known to be dysregulated in EAC; however, much of the research has focused on therapeutic resistance, not cancer prevention (57–59). C-PAC also reversed reflux-induced K05501 and K02569, both linked to antibiotic resistance, as well as K04015 which is associated with nitrate/nitrite reductases and significantly dysregulated in EAC progression (15). Predictably, agents like C-PAC that normalize transport may have efficacious roles in cancer prevention as well as adjuvant therapy. In parallel with our metabolomic pathway map results, PICRUSt also identified altered glutathione metabolism in the reflux group and mitigation by C-PAC. These data align with earlier research by our group revealing that C-PAC protects patient-derived normal primary esophageal cultures against BA-induced damage through induction of glutathione *S*-transferase theta 2 (GSTT2), a phase II detoxification enzyme with postulated roles in esophageal mucosal defense and cancer inhibition (22, 23). Similarly, we also reported that C-PAC reversed reflux-induced loss of GSTT2 while simultaneously decreasing esophageal DNA damage, supporting a protective role for this protein targeting EAC in vivo (23). PICRUSt functional analysis provides further evidence that reflux induced deleterious microbiome changes that are mitigated by C-PAC, in part through effects on transport and detoxification, contributing to EAC inhibition.

Molecular analyses further characterized C-PAC’s capacity to mitigate reflux-linked modulation of the gut microbiome, microbial metabolites, inflammation, and immune signaling. LPS is a toxic bacterial metabolite secreted by Gram-negative bacteria and known to trigger host immune responses through receptors, including TLRs and LPS-binding protein (LBP), leading to activation of NF-κB and other inflammatory signaling cascades linked to reflux-driven EAC (60). C-PAC modifies signaling associated with bacterial and viral host recognition known to be dysregulated in EAC progression, as evidenced by C-PAC modulating reflux-induced effects on TLR2–TLR4 and TLR9, as well as LBP (18, 61). C-PAC also modulates reflux induction of CD44, a multifunctional transmembrane glycoprotein that regulates TLR activity and plays a role in barrier function through tight-junction assembly (62, 63). At the transcript and protein level, C-PAC mitigated immune recruiting, proinflammatory cytokines linked to BE progression (21), markers associated with increased Gram-negative bacteria, and activation of the NF-κB pathway, which is also known to be induced by specific BAs (18, 64). To our knowledge, we show for the first time that C-PAC decreased esophageal levels of aberrant TP53 in the context of reflux in vivo, consistent with our previous in vitro research (34, 35). Mutant TP53 levels have been noted in the rat reflux-induced EAC model based on accumulation of TP53 in nuclei and the cytoplasm of bile-developed columnar-lined epithelium in EAC tissues at 40 weeks (65). C-PAC mitigated approximately 38% of high- and moderate-level mutations induced in reflux, including a high-impact stop-gain mutation in *Tp53i3*, a quinone oxidoreductase involved in DNA damage response and TP53-mediated apoptosis (46, 47). Additional non–mutation-based changes in *Tp53* may also contribute to aberrant TP53 protein expression, as noted with reflux and mitigated by C-PAC. Additional research is warranted to determine whether posttranslational modifications that are both common and effective at regulating TP53 (66) play a role, or possibly re-education of stromal TP53 is involved (67).

The gut microbiome alters oncogenic functions of *TP53* (68), raising the question of whether *TP53*-linked cancers can be inhibited by microbiome-targeting agents such as C-PAC. Orally delivered C-PAC proved efficacious at inhibiting EAC progression through mitigating reflux-induced inflammation, immune suppression, and dysbiosis while reducing levels of aberrant TP53. Additionally, C-PAC reversed reflux-induced increases in MAPK signaling via SAPK/JNK and ERK1/2 but not P38. These results are consistent with our earlier in vitro findings in BE and EAC cell lines, as well as in OE19 xenografts, following C-PAC treatment (34, 35). The sustained signaling of P38 with C-PAC in the context of reflux combined with reflux-driven increased levels of aberrant TP53, may be in part due to MAPK and TP53 crosstalk (69). Collectively, these results highlight the potent effects of C-PAC on EAC inhibition not only through microbial, but also metabolic and molecular, reprogramming of the reflux-exposed esophagus.

Despite limitations and knowledge gaps, results from the present study hold strong potential for informing future clinical studies targeting GERD, BE, and EAC inhibition. Study limitations include that BA interrogation was conducted at the tissue and stool level without capacity to assess the total BA pool. Still, in alignment with potential impact on the BA pool and associated systemic effects, reflux induction significantly elevated liver cholesterol and taurine-conjugated BAs. Microbiome species-level changes in the stool were also restricted to frequency-based measures in the current study. Furthermore, clinical trials assessing cranberry polyphenols or C-PAC have mainly focused on infections related to the urinary tract, *Helicobacter pylori*, *E*. *coli*, or select pathogenic microbes (33, 70, 71). In fact, C-PAC was tested in only a single cancer cohort, showing that 30 days of oral delivery significantly reduces serum prostate-specific antigen levels (22.5%) in prostate cancer patients (72). In summary, C-PAC represents a safe, efficacious, and widely available dietary constituent that acts as a prebiotic mitigating reflux-induced inflammation and damage in a translationally relevant rat EAC model through modulation of the gut microbiome, microbial metabolite levels, and immune signaling cascades requisite for BE progression to EAC. Additional research is warranted to fully characterize effects of C-PAC on specific immune cell populations and barrier function. Future research should also include clinical evaluations of C-PAC in patients with GERD or BE, particularly those with mutant or aberrant *TP53* or at increased risk for EAC progression based on modeling or the presence of additional risk factors. GERD and BE are the strongest known risk factors for EAC followed by obesity, especially abdominal or visceral obesity (73). A polyphenol-rich cranberry extract protects from diet-induced obesity, visceral obesity, insulin resistance, and intestinal inflammation in association with increased *Akkermansia* spp. population in the gut microbiota of mice, supporting favorable effects in the context of an additional key risk factor for EAC (74). This aligns with our results revealing lipid metabolism as a top functional microbiome change linked to C-PAC inhibition of reflux-induced EAC in the rat model. Additionally, a recent randomized placebo-controlled clinical trial reported that 8 weeks of consumption of a cranberry beverage rich in C-PAC lead to a reduction in triacylglycerol and oxidative stress levels in patients with elevated levels of C-reactive protein, supporting favorable effects in humans that parallel changes identified in preclinical models (75). Mounting evidence supports the notion that microbial alterations contribute to reflux-induced BE and progression to EAC through key roles in metabolism, inflammatory signaling, and mucosal defense. Thus, C-PAC seems especially well suited for targeting BE and progression to EAC, as it mitigates the major risk factors, as well as the biologic and molecular sequelae.

## Methods

### Sex as a biologic variable.

Male rats were exclusively used because males are disproportionately burdened by EAC (1). It is unknown whether female rats would respond similarly.

### Animals.

Following a 1-week acclimation period to the facility, 5- to 7-week-old male Sprague-Dawley rats (Crl:SD, strain code 400; Charles River Laboratories) were weighed and randomized into experimental groups for the dose-range-finding study (*n* = 24) or the long-term chemoprevention bioassay (*n* = 130). Rats were pair-housed in a controlled environment with standard light/dark and temperature conditions and were fed AIN93M purified diet (Dyets Inc.) and water or C-PAC in the drinking water available ad libitum. Body weight and food and water consumption were measured at least weekly.

### C-PAC.

C-PAC was sourced and isolated as previously described (34). Briefly, cranberries (*Vaccinium macrocarpon* Ait.) were collected at the Marucci Center for Blueberry and Cranberry Research and purified C-PAC was isolated from the Early Black cultivar using solid-phase chromatography according to well-established methodology (32, 76). This cultivar is native to the eastern United States and Canada, making it readily available for both commercial purposes and research applications. Following homogenization in 70% acetone, the mixture was filtered, pulp discarded, and the resulting C-PAC fraction concentrated under reduced pressure and the purified extract isolated using bioassay-directed fractionation. To verify the presence of A-type linkages and PAC concentration, ^13^C NMR, electrospray mass spectrometry, matrix-assisted laser desorption/ionization time-of-flight mass spectrometry, and acid-catalyzed degradation with phloroglucinol were used. Purified C-PAC was freeze-dried and stored at –80°C. To prepare C-PAC in the drinking water for study, C-PAC was dissolved in 0.001% ethanol, sonicated, and dissolved to the appropriate final concentration in vivarium water. Fresh C-PAC was replenished at least weekly.

### The rat reflux-induced EAC model.

Reflux-induced EAC was created via an esophagogastroduodenal anastomosis (EGDA) as previously described (39, 77). Briefly, incisions were made at the gastroesophageal junction and at the antimesenteric border of the duodenum, followed by side-to-side duodenoesophageal anastomosis with accurate mucosa-to-mucosa opposition. The EGDA or anastomosis of the duodenum to the gastroesophageal junction creates chronic reflux of bile and acidic gastric contents into the lower esophagus, mimicking human GERD or reflux-driven BE, the only identified precursor lesion to EAC (78). As shown in Figure 1A, the anastomosis position is critical for inducing reflux of bile mixed with acidic gastric secretions, which promotes the development of premalignancy and EAC. Our group observed that surgical failure results if the anastomosis site is not placed at the esophageal gastric junction. Positional surgical failure results in the esophagus being exposed primarily to bile reflux, which does not induce premalignancy nor cancer in this model in the absence of acidic gastric excretions. Advantages of this reflux-induced EAC model, relative to other surgical EAC models, include induction of cancer without carcinogen administration and anatomical preservation of the stomach allowing for normal stomach function and weight gain, which is key for diet-based interventions and when investigating cancers linked to energy imbalance, given that energy restriction is a potent cancer inhibitor. Other models manipulate the stomach to retard weight gain or induce weight loss (79), which may confound study results.

### Dose-range-finding study.

To determine the appropriate concentration of C-PAC to utilize in the long-term chemoprevention bioassay, we consulted the published literature and performed a 6-week safety and dose-range-finding study in Sprague-Dawley rodents (32, 33, 76). In the dose-range-finding study, rats (*n* = 6/treatment group) received water or C-PAC in the drinking water at 250, 500, or 700 μg/rat/day ad libitum. Water and C-PAC were replenished at least weekly. Blood was drawn in EDTA-treated tubes and centrifuged immediately to permit collection of plasma for serology profiling (Supplemental Figure 1B).

### Long-term chemoprevention bioassay.

The study schematic, timeline, and experimental groups are detailed in Figure 1A. The experimental groups included water, C-PAC, reflux, and C-PAC+reflux. C-PAC levels were targeted at 350 or 700 μg/rat/day in the drinking water ad libitum throughout the study; however, surgical failures in the low-dose C-PAC+reflux group resulted in insufficient sample size and exclusion of these animals from the final analysis. The previous week’s water intake was utilized to accurately prepare the targeted concentrations of C-PAC required each week (target: 700 μg/rat/day, actual resultant delivery was 690 μg/rat/day over the 40-week bioassay). Animals were sacrificed at 25 and 40 weeks after reflux-inducing surgery, and organs were either flash-frozen in liquid nitrogen for molecular analyses or fixed in neutral buffered formalin for 24 hours prior to standard processing, paraffin embedding, sectioning, and hematoxylin and eosin (H&E) staining for histopathological evaluation of tissues. Prior to necropsy, fresh fecal pellets were flash-frozen in liquid nitrogen and stored at –80°C until further processing for microbiome analysis.

### Necropsy and histopathology evaluation.

At necropsy, rat blood, esophageal, and major organ tissues were harvested for evaluation. Each esophagus was harvested intact, split longitudinally, and characterized for gross changes and the anastomosis site confirmed in terms of position and size opening (≥0.5 cm). Neutral buffered formalin–fixed esophagi (6–8 animals per treatment group) were processed at the Medical College of Wisconsin Histology Core (Milwaukee, Wisconsin, USA). Tissues were paraffin-embedded, 25 sections (5 μm) prepared, and every fifth section stained with H&E. Histopathological evaluation of full-thickness sections included scanning the entire length of each esophagus (50 fields on average) at ×100 and blinded grading of each field as normal, hyperplasia, LGD, HGD, or EAC.

### Metabolomics and bioinformatic analyses.

Sample preparation, extraction, metabolite identification, data handling, and initial analysis for untargeted metabolomics were conducted by Metabolon, Inc. Homogenized samples were extracted in methanol and dried under vacuum to remove organic solvent. Samples were characterized using reverse-phase ultra-high-performance liquid chromatography–tandem mass spectrometry. Compounds were identified based on comparison to a library of authenticated standards maintained by Metabolon, Inc. and statistical analysis conducted in ArrayStudio on log-transformed data. Six samples per treatment group were characterized for metabolomics.

Statistically significant (*P* ≤ 0.05) metabolites induced by reflux and directly reversed by C-PAC (*n* = 200) were analyzed in Metacore and Cortellis Solution software (https://clarivate.com/products/metacore/, Clarivate Analytics). All *P* value and FDR significant Pathway Maps, Process Networks, Diseases, Go Processes, Metabolic Networks, GO Molecular Functions, and GO Localizations for each comparison are exported. Significance was calculated using the hypergeometric test as previously described (80), and it should be noted that Metacore had the highest recognition of metabolites at 86%, which contrasts with reduced metabolite recognition obtained in other programs evaluated, including Metaboanalyst (65%), Metscape (45%), or PaintOmics (35%).

Quantitative validation of specific BAs was conducted at the University of Michigan Metabolomics Core as previously described using reverse-phase liquid chromatography–mass spectrometry (81). BAs were measured using negative ionization mode triple quadrupole multiple reaction monitoring methods, as previously described (81).

### DNA isolation.

Total genomic DNA was isolated from fecal pellets using the PowerLyzer PowerSoil DNA Isolation Kit (Mo Bio Laboratories) according to the manufacturer’s instructions with 1 modification; fecal samples were transferred to glass bead tubes with 750 μL bead solution, incubated 65°C for 10 minutes, and transferred to 95°C for 10 minutes. DNA samples were eluted with 100 μL of Solution C6 and stored at –80°C until sequencing.

### Microbiome 16S rRNA gene sequencing and bioinformatic analyses.

PCR amplicon libraries targeting the 16S rRNA–encoding genes present in metagenomic DNA were produced using a barcoded primer set adapted for the Illumina MiSeq. DNA sequence data were generated using Illumina paired-end sequencing at the Environmental Sample Preparation and Sequencing Facility at Argonne National Laboratory (Lemont, Illinois, USA) as previously described (82). Amplicons were sequenced on a 151-bp × 12-bp × 151-bp MiSeq run using customized sequencing primers and procedures. Sequence data were processed in QIIME using published protocols (82). Operational taxonomic units (OTUs) were identified using Greengenes (https://greengenes.secondgenome.com) and aligned with PyNAST (https://biocore.github.io/pynast/) to construct phylogenetic trees using Fast Tree 2.0 (http://www.microbesonline.org/fasttree/). Sequencing data have been deposited to the NCBI Gene Expression Omnibus (GEO GSE254633). Plotting and statistical analyses were conducted in R v3.3.0 (https://cran-archive.r-project.org/bin/windows/base/old/3.3.0/) using phyloseq and EdgeR. There were 5–7 animals per treatment group included in this analysis, with sequencing results in Supplemental Table 16.

Diversity (α and β) was assessed using the Qiagen CLC Genomics Workbench Microbial Genomics Module 20.0 (https://digitalinsights.qiagen.com/). Briefly, sequencing files were imported into the software, followed by OTU clustering using the default parameters. A phylogenetic tree was reconstructed to perform α- and β-diversity analysis. Chao1 bias-corrected estimation was used for determining α diversity and β diversity was determined using the Bray-Curtis dissimilarity and Jaccard similarity indexes. The significance of β diversity was assessed using the permutational multivariate analysis of variance (PERMANOVA). The differential abundance analysis tool was used to determine significant changes in OTUs across samples, and the heatmap was generated using the top 25 most significantly changed OTUs (*P* ≤ 0.05, FDR ≤ 0.05, and Bonferroni-adjusted *P* ≤ 0.05).

### Species-level microbiome identification and analysis.

The same genomic DNA submitted for 16S rRNA gene sequencing was assessed for species-level changes using the Axiom Microbiome Array on the GeneTitan Multi-channel instrument (Thermo Fisher Scientific) at the University of North Carolina at Chapel Hill High-throughput Sequencing facility following the manufacturer’s instructions. Data were analyzed using the Axiom Microbial Detection Analysis Software (MiDAS; Thermo Fisher Scientific) following the manufacturer’s and published protocols (83) to determine the frequency of microbial families and species in each treatment group. Fisher’s exact tests were used to determine significant differences in families or species in gut microbiomes, with tests for trend using the χ^2^ test (*P* ≤ 0.05). This analysis included 6–8 animals per treatment group.

### PICRUSt analysis for functional microbial metagenomic profiling.

Predicted functions of bacterial communities were identified in PICRUSt based on published methods (84). PICRUSt utilizes existing annotations of gene content and 16S rRNA gene copy number from reference bacterial and archaeal genomes to predict the presence of gene families. Files were formatted as “.biom” and imported into PICRUSt for OTU tables generated using the pick_closed_references_otus script in QIIME (85). The OTU abundances were normalized against the reference 16S rRNA gene copy numbers using the normalize_by_copy_number.py script, which divides each OTU by the known or predicted 16S rRNA gene copy number abundance. The normalized table was used to predict functions for the metagenome using the script predict_metagenome.py. The script generated a table of function counts as KO by sample IDs (86). The accuracy of these predictions was evaluated by using the nearest sequenced taxon index (NSTI). Lastly, predicted KO abundances for the given OTU table were collapsed into individual KEGG pathways by using the script categorize_by_functions.py. The output files were uploaded to Statistical Analysis of Taxonomic and Functional Profiles (STAMP; https://beikolab.cs.dal.ca/software/STAMP), unclassified reads removed, and statistical analysis performed to determine differences between functional pathway data (KEGG pathways level 2 and 3), as well as 125 identified significant KEGG KOs by ANOVA with Storey’s FDR correction for multiple comparisons and Tukey’s post hoc test. Mean (± standard deviation) NTSI values for water, C-PAC, reflux, and C-PAC+reflux were 0.11 ± 0.03, 0.12 ± 0.01, 0.10 ± 0.02, and 0.12 ± 0.02, respectively, indicating samples were suitable for PICRUSt analysis (84).

### RNA isolation and expression analysis.

RNA was isolated from rat lower esophageal tissues using the RNeasy Fibrous Tissue Kit (Qiagen). Samples were homogenized in 400 μL of Buffer RLT with β-mercaptoethanol for three 10-second pulses with a homogenizer (Pro-Scientific Inc.) set on level 2. Three to 6 samples per treatment group were evaluated. RNA was purified following manufacturer’s instructions and eluted in 20 μL of Ambion RNA Storage Solution (Thermo Fisher Scientific). RNA concentration and quality were measured using the RNA 6000 Pico kit on the Bioanalyzer 2100 capillary electrophoresis system (Agilent) and stored at –80°C. One microgram of RNA per sample was reverse transcribed using the iScript Advanced cDNA Synthesis Kit (Bio-Rad) following the manufacturer’s protocol. Expression levels of 85 genes were assessed using the PrimePCR Antibacterial Response (SAB Target List) R384 rat plate (catalog 10047065, Bio-Rad) using SsoAdvanced Universal SYBR Green Supermix (Bio-Rad). Real-time PCR was performed on the CFX384 real-time PCR system (Bio-Rad) following the manufacturer’s protocol. Data were analyzed using CFX Manager (Bio-Rad), in which relative changes in gene expression were calculated by 2^–ΔΔCt^, where ΔΔCt = ΔCt_reflux_ – ΔCt_water_ or ΔΔCt = ΔCt_C-PAC+reflux_ – ΔCt_reflux_, as previously described (87). Data were normalized to expression levels of *Gapdh* and *Hsp90ab1*, with statistical significance determined by Student’s *t* test. *P* values of 0.05 or less were considered significant.

### Bacterial response genes identified in human EAC.

The publicly available GEO data set (GSE26886), originally contributed by Wolfgang Kemmner (88), was downloaded from the NCBI GEO website. For comparison purposes, log_2_-transformed data were organized by histopathology and contained a total of 69 human esophageal samples of mixed pathologies. Esophageal squamous cell data (*n* = 9) were removed from our analysis. The remaining data consisted of normal esophageal squamous epithelium (*n* = 19), BE (*n* = 20), and EAC (*n* = 21). Normal epithelium was compared to both BE and EAC. A Student’s *t* test with Benjamini-Hochberg FDR correction was utilized to determine statistically significant differences (FDR ≤ 0.05). A second publicly available GEO data set (GSE193946) containing human tissues from BE patients with LGD to BE with HGD and EAC (89) was also compared to gene-level changes in the rat esophagus. Results in Table 1 denoted by a superscript “A” in column 2 indicate that changes with reflux in the rat esophagus were consistent with significant dysregulation of the same marker in either human GEO data set.

### Protein isolation and Western blot analysis.

Rat lower esophageal lysates were prepared by homogenization (PRO Scientific Inc.) in T-PER Tissue Protein Extraction Reagent (Thermo Fisher Scientific) with cOmplete EDTA-free protease inhibitor cocktail and PhosSTOP phosphatase inhibitors (Roche) according to the manufacturer’s instructions. Protein levels in soluble lysates were quantified using the DC protein assay (Bio-Rad) and 15–30 μg/lane loaded into precast 4%–20% and 10% Mini-Protean TGX gels (Bio-Rad). Immunoblotting was performed using commercially available antibodies (Supplemental Table 17). Images were captured via the ChemiDoc Molecular Imager and band quantification with ImageLab analysis software (both Bio-Rad). Expression values normalized to appropriate loading controls were determined by chemiluminescent immunodetection, with fold-change from the water-alone group reported.

### Data integration of metabolites and antimicrobial genes.

To build integrated networks, significant antibacterial response genes (*P* value ≤ 0.05, *n* = 47) and significant metabolites (*P* value ≤ 0.05) for reflux versus water (*n* = 319) and C-PAC+reflux versus reflux (*n* = 264) were uploaded individually into Metacore and Cortellis Solution software. The C-PAC+reflux versus reflux gene and metabolite lists were activated and the analyze network feature applied to build networks with 100 nodes using all compound-target interactions. The “analyze network” feature first builds 1 large network, and this network is then divided into smaller fragments based on the chosen node size for easier visualization. The result is a list of 30 partially overlapping networks, each with associated GO processes, *P* values, *g* scores, and *z* scores calculated as previously described (80) and select networks were visualized.

### Statistics.

Additional statistical analyses not described elsewhere were performed in GraphPad Prism software. Body weight, food consumption, and water consumption data were analyzed for differences between all treatment groups using repeated-measures ANOVA where time was considered a variable and with Tukey’s post hoc test. All *P* values of 0.05 or less were considered significant.

### Study approval.

All experimental procedures were conducted in accordance with the protocol approved by the Institutional Laboratory Animal Care and Use Committee at the Medical College of Wisconsin (AUA3095) and consistent with the NIH *Guide for the Care and Use of Laboratory Animals* (National Academies Press, 2011).

### Data availability.

Sequencing data are available at the NCBI (GEO GSE254633). Additional data required to evaluate the study conclusions are present in the manuscript or the Supporting Data Values Excel file. Other data are available upon reasonable request made to the corresponding author.

## Author contributions

LAK and KMW conceived and designed the experiments. LAK, KMW, CLH, and YZ collected and contributed data. ABH prepared cranberry proanthocyanidins. LAK, KMW, CLH, YZ, JHR, JAA, MW, BAT, and JLC performed data analyses. LAK and KMW wrote the manuscript assisted by CLH, YZ, BAT, JLC, ABH, JHR, JAA, and MW. All authors reviewed and approved the final manuscript.

## Supplementary Material

Supplemental data

Unedited blot and gel images

Supplemental table 16

Supporting data values

## Figures and Tables

**Table 1:**
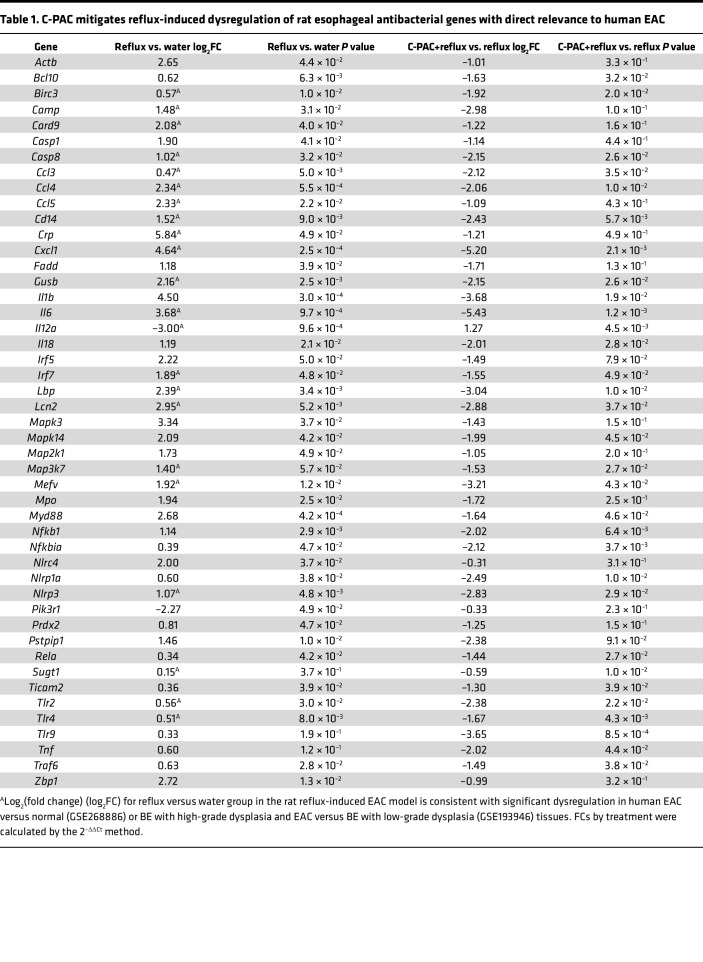
C-PAC mitigates reflux-induced dysregulation of rat esophageal antibacterial genes with direct relevance to human EAC

**Table 2:**
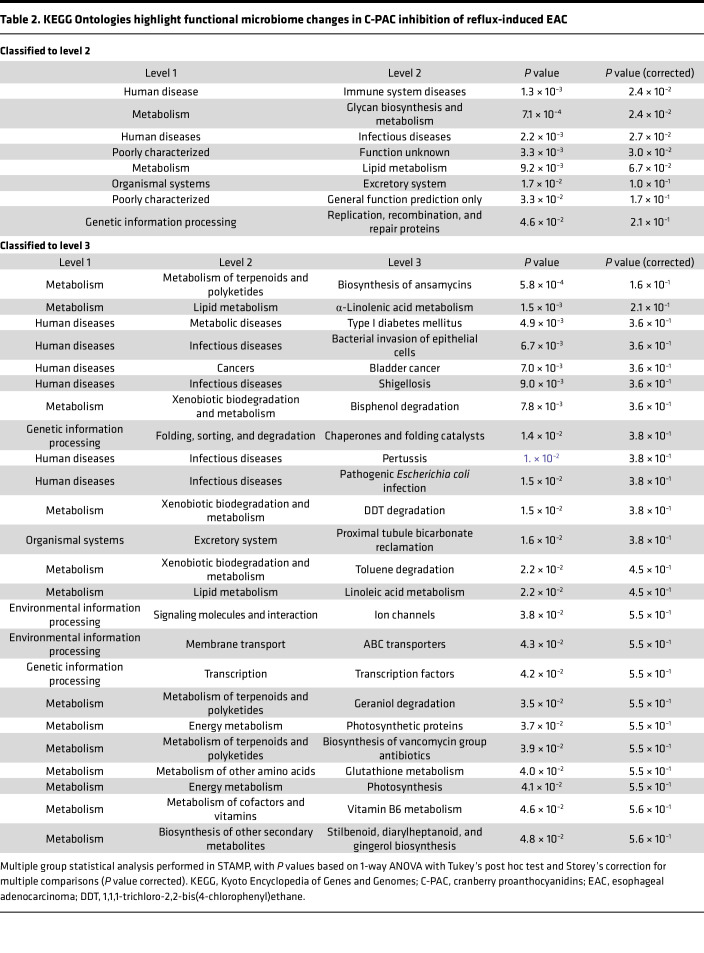
KEGG Ontologies highlight functional microbiome changes in C-PAC inhibition of reflux-induced EAC

## Version 1 02/08/2024

In-Press Preview

## Version 2 03/22/2024

Electronic publication
