# Supplementary material for: Prebiotic proanthocyanidins inhibit bile reflux–induced esophageal adenocarcinoma through reshaping the gut microbiome and esophageal metabolome
Source: JCI Insight. 2024 Feb 8;9(6):e168112. doi: 10.1172/jci.insight.168112 (PMC11063939; doi:10.1172/jci.insight.168112)
Supplement: Supplemental data [file jciinsight-9-168112-s046.pdf]

Prebiotic proanthocyanidins inhibit bile reflux-induced esophageal adenocarcinoma through reshaping the gut microbiome and esophageal metabolome

Katherine M. Weh, Connor L. Howard, Yun Zhang, Bridget A. Trip, Jennifer L. Clarke, Amy B. Howell, Joel H. Rubenstein, Julian A. Abrams, Maria Westerhoff and Laura A. Kresty

<u>Supplementary Figures and Tables</u>

Supplementary Figure 1. Measured animal parameters for dose range finding study and C-PAC safety.
Supplementary Figure 2. Measured animal parameters for rat model of reflux-induced EAC.
Supplementary Figure 3. Fecal microbiome sample hierarchical clustering and beta diversity measurements.
Supplementary Figure 4. Integrative analysis of significant untargeted metabolomics and gene expression antimicrobial pathway results.

Supplementary Table 1. Mean animal body weight (grams) by week of study.
Supplementary Table 2. Mean animal body weight gain (grams) by week of study.
Supplementary Table 3. Mean animal food consumption in (grams) by week of study.
Supplementary Table 4. Mean water consumption (milliliters) by week of study.
Supplementary Table 5. Frequency of gut microbiome species by treatment group in a rat model of reflux-induced EAC.
Supplementary Table 6. Frequency of gut microbiome families by treatment group in a rat model of reflux-induced EAC.
Supplementary Table 7. Pathway maps (n=45) up-regulated by reflux and directly reversed by C-PAC (n=140 metabolites; FDR $\leq$0.05).
Supplementary Table 8. Pathway maps (n=21) down-regulated by reflux and directly reversed by C-PAC (n=60 metabolites; FDR $\leq$0.05).
Supplementary Table 9. Metabolic networks (n=29) up-regulated by reflux and directly reversed by C-PAC (n=140 metabolites; FDR $\leq$0.05).
Supplementary Table 10. Metabolic networks (n=10) down-regulated by reflux and directly reversed by C-PAC (n=60 metabolites; FDR $\leq$0.05).
Supplementary Table 11. Process networks (n=5) up-regulated by reflux and directly reversed by C-PAC (n=140 metabolites; FDR $\leq$0.05).
Supplementary Table 12. Process networks (n=3) down-regulated by reflux and directly reversed by C-PAC (n=60 metabolites; FDR $\leq$0.05).
Supplementary Table 13. C-PAC alters bacterial gene expression in the normal rat esophagus.
Supplementary Table 14. Network list for integration of significant antimicrobial pathway genes and esophageal metabolites in C-PAC+reflux vs reflux.
Supplementary Table 15. PICRUSt multigroup analysis of fecal microbiomes from water, C-PAC, reflux and C-PAC+reflux treated animals (n=125 OTU ID).
Supplementary Table 16. Excel file of microbiome OTUs. (separate file)
Supplementary Table 17. Antibodies utilized in this research.

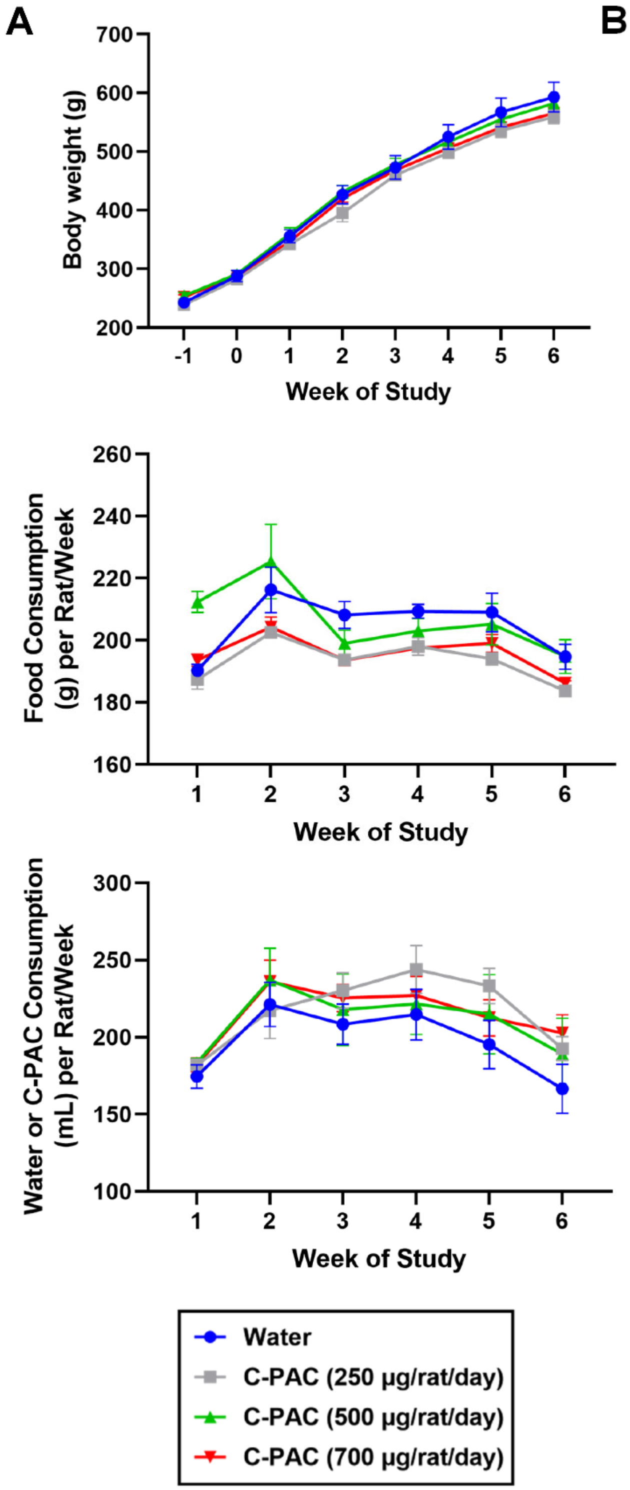

B

Serum Profile Measurements for C-PAC Dose Range Finding Study
Glucose
Aspartate Amino Transferase (AST/GOT)
Alanine Amino Transferase (ALT/GPT)
Alkaline Phosphatase
Total Bilirubin
Cholesterol
Total protein
Albumin
Globulin
Urea N
Creatinine
Phosphorous
Calcium
Sodium
Potassium
Chloride
Bicarbonate
Anion Gap
Gamma-GT
A/G ratio
Urea/Creatinine Ratio
Sodium/Potassium Ratio

Supplementary Figure 1. Measured animal parameters for dose range finding study and C-PAC safety. A six-week preliminary study was performed to assess multiple levels of C-PAC in rats (250, 500 or 700 µg/rat/day) in the drinking water *ad libitum* measuring (A) body weight (g), food consumption (g) and water or C-PAC consumption (mL). Each group is denoted by a different colored line: Water (blue), 250 µg/rat/day (gray), 500 µg/rat/day (green) and 700 µg/rat/day (red). Results were assessed using two way ANOVA with repeated measures with no significant findings for body weight, food consumption or water/C-PAC consumption over the 6 week study. (B) Profile of 22 serum measurements for C-PAC safety for animals in the six-week study. No statistical differences were observed between water and each C-PAC group for any of the serum measurements (Student's *T*-test, two-sided, $P<0.05$).

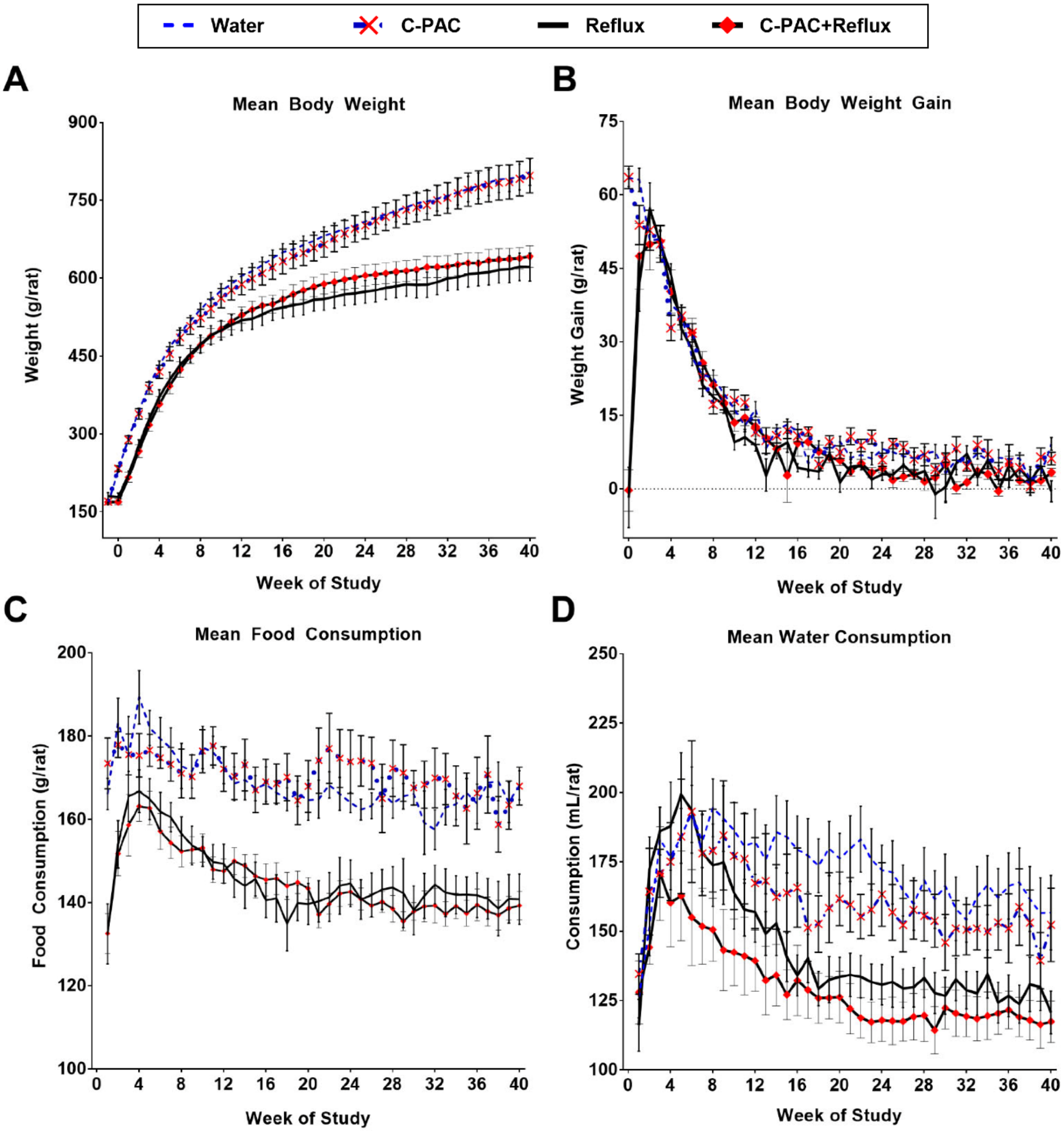

Supplementary Figure 2. Measured animal parameters for rat model of reflux-induced EAC. (A) Mean body weight (g/rat), (B) mean body weight gain (g/rat), (C) mean food consumption (g/rat) and (D) mean water consumption (mL/rat) was repeatedly measured in animals on study from 0-40 weeks. Data are shown as the mean ± standard error of the mean for a minimum of n=10 animals per treatment group. Treatment groups are denoted as follows: water (blue striped line), C-PAC (blue dotted line with red cross marks), Reflux (black solid line) and C-PAC+Reflux (black solid line with red diamond).

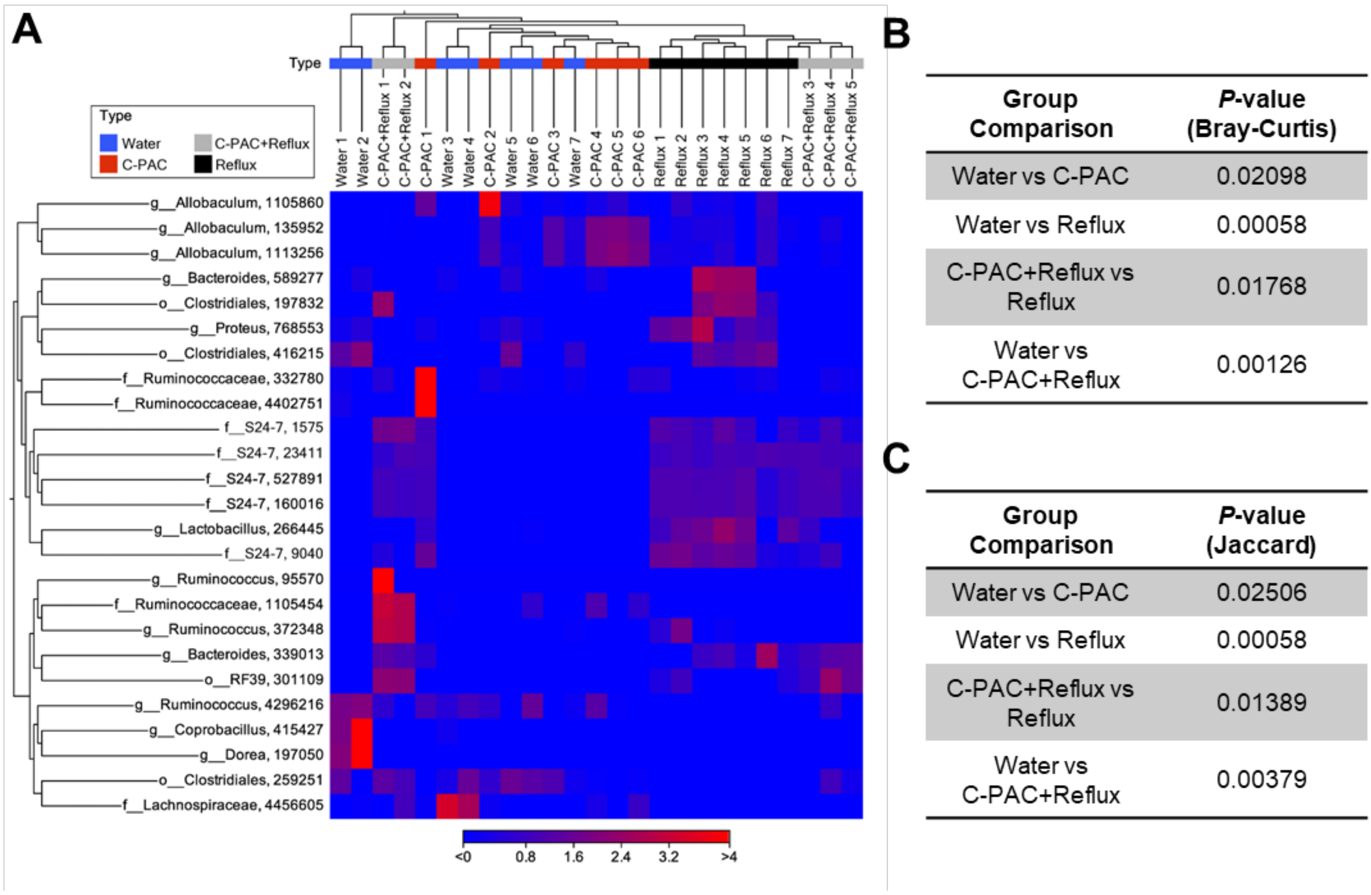

B

Group Comparison	*P*-value (Bray-Curtis)
Water vs C-PAC	0.02098
Water vs Reflux	0.00058
C-PAC+Reflux vs Reflux	0.01768
Water vs C-PAC+Reflux	0.00126

C

Group Comparison	*P*-value (Jaccard)
Water vs C-PAC	0.02506
Water vs Reflux	0.00058
C-PAC+Reflux vs Reflux	0.01389
Water vs C-PAC+Reflux	0.00379

Supplementary Figure 3. Fecal microbiome sample hierarchical clustering and beta diversity measurements. (A) Hierarchical clustering of fecal microbiome samples for all treatment groups were plotted based on the top 25 significantly altered bacterial taxa ($P \leq 0.05$ with Benjamini Hochberg correct for FDR$\leq$0.05). Increases in abundance are noted from changes in color from blue to red. Beta diversity was assessed based on (B) Bray-Curtis index (bacterial taxa abundance) and (C) Jaccard index (bacterial taxa presence or absence). C-PAC, cranberry proanthocyanidins.

A

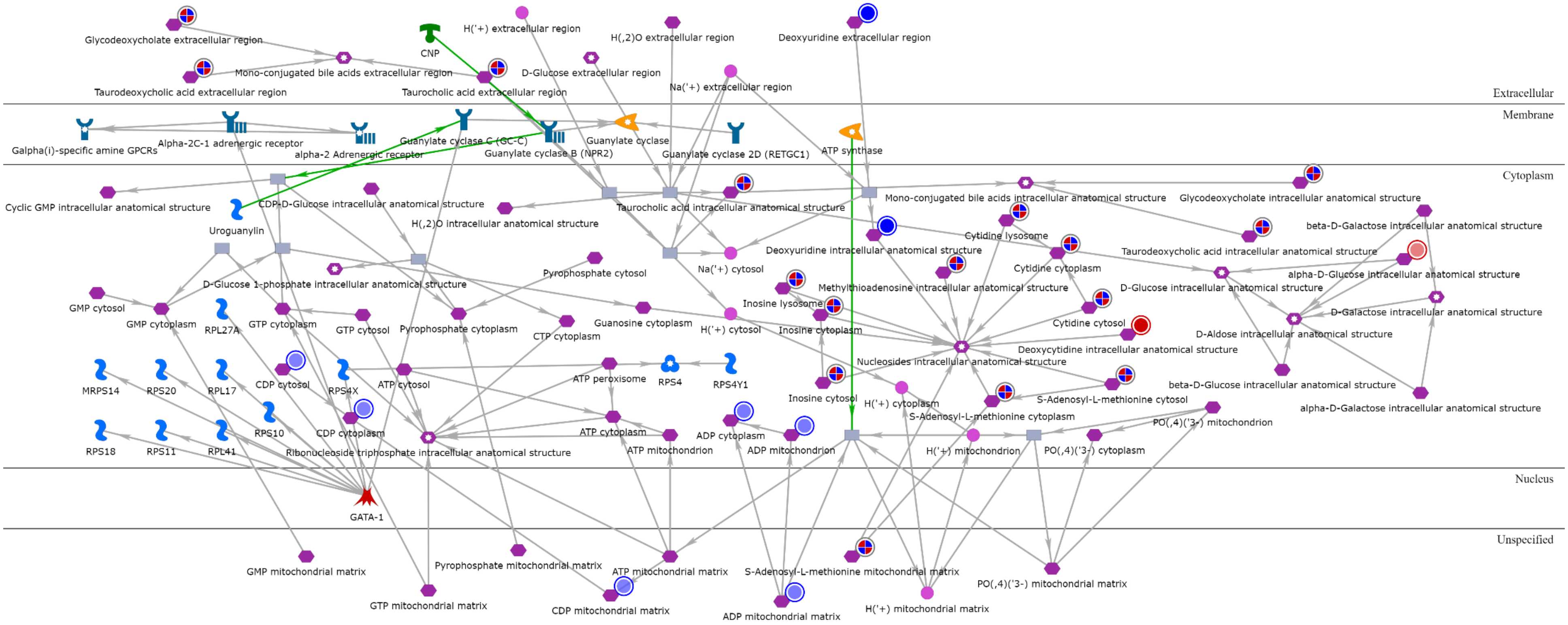

Integration Key (Adapted from Metacore Manual)

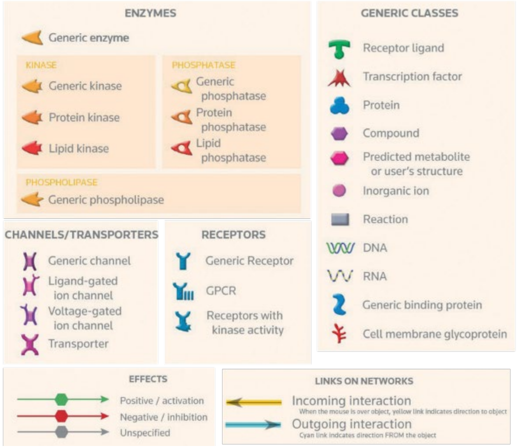

B

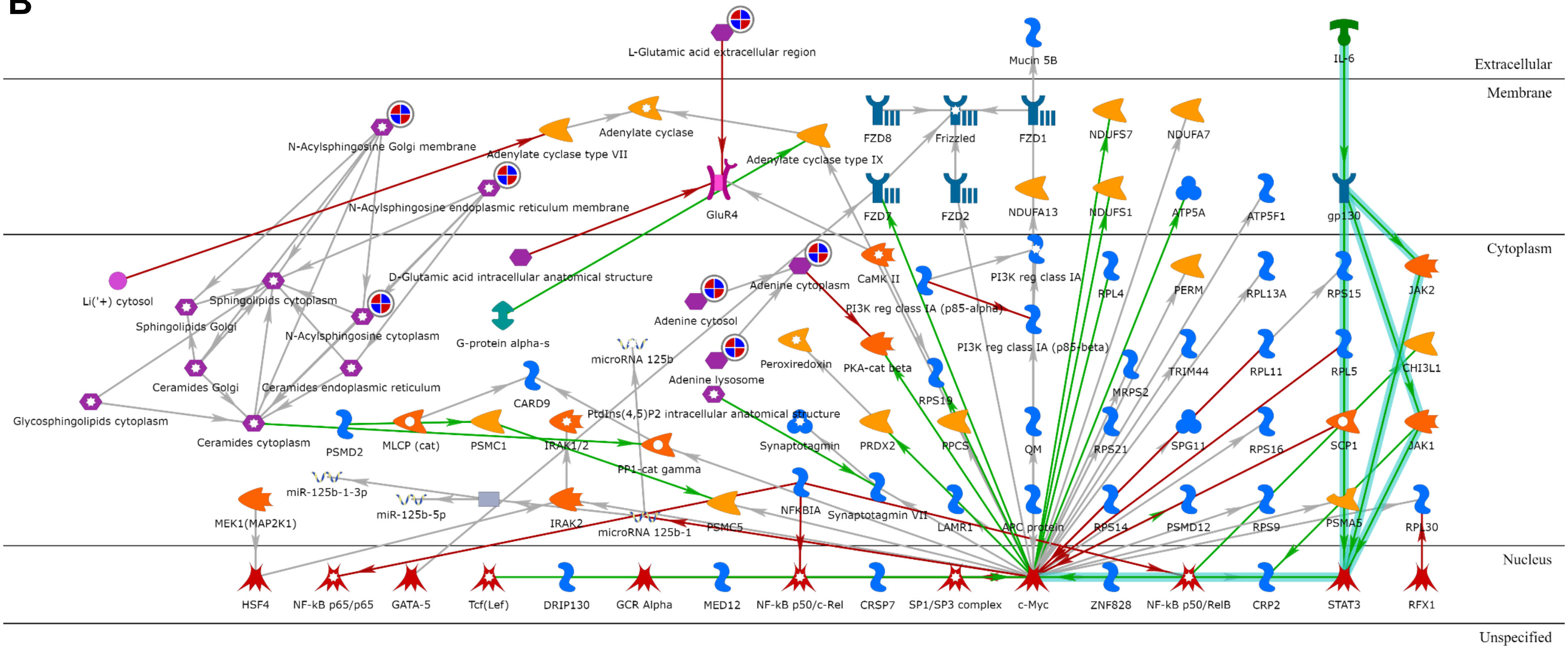

Supplementary Figure 4. Integrative analysis of significant untargeted metabolomics and gene expression antimicrobial pathway results. The Metacore integration tool was utilized to produce a network list from the 319 and 264 significantly dysregulated metabolites in the reflux vs water and C-PAC+reflux vs reflux comparisons, respectively, and the 49 significantly altered genes from the antibacterial response pathway plate. (A) Pictorial representation of Network 2 which is focused on bile acid signaling through taurocholic acid and (B) Network 5 highlighting signaling through *NF-κ*B with *c-Myc/IL-6/STAT3* as central signaling nodes. Metabolites and genes directly altered by reflux and reversed by C-PAC in the dataset are depicted as blue and/or red pie charts, respectively. An integration key at the bottom of the figure denotes symbol identification for panels in the figure. A complete list of the significant networks from the integrative analysis is found in online supplemental table 15.

Supplementary Table 1. Mean animal body weight (grams) by week of study.

	Water	C-PAC	Reflux	C-PAC+Reflux
Week -1	166.21 ± 2.11	169.85 ± 3.18	180.60 ± 6.18	169.31 ± 5.10
Week 0	229.68 ± 3.02	233.38 ± 5.17	178.80 ± 7.30	169.50 ± 5.41
Week 1	292.75 ± 4.68[d]	288.77 ± 7.55[b]	220.90 ± 8.05	217.00 ± 9.43[a,b]
Week 2	343.33 ± 6.39[d]	338.54 ± 9.82[b]	278.10 ± 10.87	266.94 ± 12.29[a,b]
Week 3	394.40 ± 7.44[d]	387.92 ± 11.58	328.20 ± 11.98	317.44 ± 12.39[a]
Week 4	432.13 ± 8.28[d]	420.15 ± 12.76	372.00 ± 13.18	357.69 ± 14.43[a]
Week 5	467.53 ± 8.97[d]	454.54 ± 13.54	404.60 ± 14.63	392.44 ± 15.00[a]
Week 6	494.27 ± 10.10[d]	485.62 ± 14.75	433.10 ± 15.12	424.31 ± 14.93[a]
Week 7	517.53 ± 10.84	507.85 ± 15.90	454.30 ± 16.28	449.97 ± 14.34
Week 8	540.13 ± 11.82[d]	523.62 ± 17.05	473.00 ± 17.40	471.13 ± 14.43[a]
Week 9	559.53 ± 12.99[d]	541.54 ± 17.79	490.10 ± 18.41	488.44 ± 14.75[a]
Week 10	575.80 ± 13.40[d]	561.69 ± 18.63	499.60 ± 18.91	501.94 ± 15.22[a]
Week 11	588.67 ± 14.15[c,d]	576.77 ± 18.69	510.10 ± 19.39[a]	516.38 ± 15.14[a]
Week 12	604.93 ± 14.68[c,d]	588.31 ± 20.13	519.00 ± 19.73[a]	529.06 ± 15.67[a]
Week 13	613.33 ± 14.92[c,d]	599.31 ± 21.55	521.80 ± 20.35[a]	539.38 ± 16.2[a]
Week 14	625.47 ± 15.23[c,d]	609.31 ± 22.51	530.10 ± 21.07[a]	547.50 ± 16.91[a]
Week 15	638.53 ± 15.77[c,d]	620.92 ± 23.14[c]	539.50 ± 20.94[a,b]	550.19 ± 17.91[a]
Week 16	650.27 ± 16.23[c,d]	632.38 ± 23.57[c,d]	543.90 ± 20.76[a,b]	559.50 ± 17.50[a,b]
Week 17	655.40 ± 16.48[c,d]	642.46 ± 23.50[c,d]	547.80 ± 21.58[a,b]	569.06 ± 17.89[a,b]
Week 18	664.20 ± 16.98[c,d]	648.38 ± 24.06[c,d]	551.30 ± 21.79[a,b]	576.69 ± 18.22[a,b]
Week 19	672.07 ± 17.11[c,d]	658.31 ± 24.54[c,d]	558.50 ± 21.79[a,b]	583.13 ± 18.70[a,b]
Week 20	681.73 ± 17.34[c,d]	665.15 ± 24.79[c,d]	559.80 ± 21.57[a,b]	588.94 ± 19.21[a,b]
Week 21	688.07 ± 18.01[c,d]	676.38 ± 25.21[c,d]	564.30 ± 21.81[a,b]	592.50 ± 19.54[a,b]
Week 22	695.00 ± 18.45[c,d]	685.85 ± 25.49[c,d]	569.00 ± 21.67[a,b]	597.69 ± 19.57[a,b]
Week 23	701.13 ± 18.99[c,d]	695.23 ± 25.46[c,d]	570.90 ± 21.83[a,b]	601.00 ± 20.02[a,b]
Week 24	709.27 ± 19.30[c,d]	701.77 ± 25.90[c,d]	573.90 ± 22.05[a,b]	605.19 ± 20.37[a,b]
Week 25	716.73 ± 19.55[c,d]	710.54 ± 26.09[c,d]	576.80 ± 22.29[a,b]	607.00 ± 19.80[a,b]
Week 26	723.40 ± 20.31[c,d]	718.46 ± 26.52[c,d]	581.60 ± 22.48[a,b]	609.50 ± 20.11[a,b]
Week 27	729.60 ± 20.98[c,d]	724.00 ± 26.68[c,d]	584.40 ± 22.37[a,b]	612.50 ± 19.85[a,b]
Week 28	736.80 ± 21.57[c,d]	732.08 ± 27.90[c,d]	588.00 ± 22.44[a,b]	614.00 ± 20.10[a,b]
Week 29	744.40 ± 22.03[c,d]	736.15 ± 27.73[c,d]	586.90 ± 24.98[a,b]	616.31 ± 19.70[a,b]
Week 30	747.60 ± 22.91[c,d]	740.85 ± 28.03[c,d]	587.30 ± 24.39[a,b]	621.31 ± 20.67[a,b]
Week 31	753.47 ± 23.96[c,d]	750.15 ± 29.08[c,d]	592.50 ± 24.01[a,b]	621.50 ± 20.53[a,b]
Week 32	758.60 ± 24.36[c,d]	754.69 ± 29.45[c,d]	599.60 ± 23.86[a,b]	622.81 ± 20.59[a,b]
Week 33	765.80 ± 24.41[c,d]	763.54 ± 30.00[c,d]	602.00 ± 24.79[a,b]	626.44 ± 20.70[a,b]
Week 34	771.60 ± 24.53[c,d]	770.62 ± 31.86[c,d]	607.90 ± 24.73[a,b]	629.44 ± 20.33[a,b]
Week 35	777.93 ± 24.69[c,d]	775.08 ± 31.88[c,d]	609.70 ± 24.54[a,b]	628.94 ± 20.48[a,b]
Week 36	784.00 ± 25.02[c,d]	779.62 ± 32.73[c,d]	611.70 ± 24.78[a,b]	634.00 ± 21.21[a,b]
Week 37	790.20 ± 25.15[c,d]	783.85 ± 33.27[c,d]	615.90 ± 25.45[a,b]	635.69 ± 21.07[a,b]
Week 38	792.07 ± 25.07[c,d]	785.31 ± 32.98[c,d]	617.50 ± 26.23[a,b]	636.94 ± 20.51[a,b]
Week 39	795.07 ± 24.72[c,d]	791.42 ± 33.07[c,d]	622.20 ± 26.64[a,b]	638.63 ± 20.52[a,b]
Week 40	804.20 ± 25.27[c,d]	797.49 ± 33.20[c,d]	621.70 ± 27.09[a,b]	642.00 ± 20.67[a,b]

Values are reported as mean body weight per rat in grams ± SEM and data were analyzed by Two-way ANOVA with repeated measures and Tukey's post-hoc test with time considered a variable ($P \leq 0.05$). Treatment groups were significantly different from a = Water, b = C-PAC, c = Reflux and d = C-PAC+Reflux.

Supplementary Table 2. Mean animal body weight gain (grams) by week of study.

	Water	C-PAC	Reflux	C-PAC+Reflux
Week 0	63.47 ± 1.79[c,d]	63.54 ± 2.30[c,d]	-1.80 ± 6.17[a,b]	-0.31 ± 4.23[a,b]
Week 1	63.07 ± 2.37[b,c,d]	53.85 ± 4.02[a,c]	42.10 ± 5.92[a,b]	47.50 ± 6.70[a]
Week 2	50.67 ± 1.98	52.77 ± 4.08	57.20 ± 5.24	49.94 ± 5.27
Week 3	51.07 ± 1.77	50.00 ± 3.73	50.10 ± 2.46	50.50 ± 3.37
Week 4	37.73 ± 1.46	32.85 ± 2.61[c]	43.80 ± 2.13[b]	40.25 ± 4.40
Week 5	35.40 ± 2.08	35.23 ± 1.78	32.60 ± 2.13	34.75 ± 2.33
Week 6	26.73 ± 1.58	31.85 ± 1.87	28.50 ± 1.88	31.88 ± 2.90
Week 7	23.27 ± 1.16	22.77 ± 2.38	21.20 ± 2.37	25.69 ± 2.36
Week 8	22.60 ± 1.64	17.23 ± 1.92	18.70 ± 2.08	21.19 ± 1.93
Week 9	19.40 ± 1.43	18.38 ± 2.31	17.10 ± 1.70	17.31 ± 1.72
Week 10	16.27 ± 0.79	18.00 ± 2.18	9.50 ± 1.58	13.50 ± 1.81
Week 11	12.87 ± 1.21	17.62 ± 1.46	10.50 ± 1.59	14.44 ± 1.54
Week 12	16.27 ± 1.52	11.54 ± 2.52	8.90 ± 1.22	12.69 ± 1.93
Week 13	8.33 ± 1.34	9.85 ± 2.60	2.80 ± 3.30	10.31 ± 0.88
Week 14	12.13 ± 0.95	10.85 ± 1.56	8.30 ± 2.16	8.13 ± 1.30
Week 15	13.07 ± 1.19[d]	11.92 ± 1.70[d]	9.40 ± 1.97	2.78 ± 5.47[a,b]
Week 16	11.73 ± 1.50	10.85 ± 1.23	4.30 ± 0.84	9.31 ± 2.64
Week 17	5.31 ± 1.60	11.69 ± 0.93	3.90 ± 1.51	9.56 ± 1.57
Week 18	8.80 ± 1.01	5.00 ± 1.35	3.50 ± 1.54	7.63 ± 1.29
Week 19	7.87 ± 1.10	9.62 ± 1.20	7.20 ± 1.29	6.44 ± 1.05
Week 20	9.67 ± 1.02	7.46 ± 1.30	1.30 ± 2.01	5.81 ± 1.06
Week 21	6.33 ± 1.05	10.69 ± 1.71	4.50 ± 1.53	3.56 ± 0.94
Week 22	6.93 ± 1.46	8.85 ± 1.28	4.70 ± 0.83	5.19 ± 0.80
Week 23	6.13 ± 1.26	10.54 ± 1.43	1.90 ± 1.85	3.31 ± 1.09
Week 24	8.13 ± 1.33	5.92 ± 1.33	3.00 ± 1.26	4.19 ± 0.90
Week 25	7.47 ± 1.62	9.23 ± 1.01	2.90 ± 0.95	1.81 ± 1.59
Week 26	6.67 ± 1.59	8.38 ± 1.25	4.80 ± 1.66	2.50 ± 1.02
Week 27	6.20 ± 1.30	6.08 ± 2.28	2.80 ± 0.85	3.00 ± 1.07
Week 28	7.20 ± 1.33	6.92 ± 2.25	3.60 ± 1.90	1.50 ± 1.84
Week 29	7.60 ± 1.44[c]	3.92 ± 1.21	-1.10 ± 5.02[a]	2.31 ± 1.47
Week 30	3.20 ± 5.74	6.38 ± 0.94	0.40 ± 3.19	5.00 ± 2.04
Week 31	5.87 ± 1.92	8.23 ± 2.37[d]	5.20 ± 2.33	0.19 ± 1.25[b]
Week 32	5.13 ± 1.13	4.54 ± 0.92	7.10 ± 1.68	1.31 ± 1.40
Week 33	7.20 ± 1.12	8.92 ± 1.72	2.40 ± 1.75	3.63 ± 1.33
Week 34	5.80 ± 1.37	7.08 ± 2.94	5.90 ± 1.29	3.00 ± 1.10
Week 35	6.33 ± 1.67	3.62 ± 1.76	1.80 ± 0.89	-0.50 ± 0.94
Week 36	6.07 ± 1.64	5.38 ± 3.63	2.00 ± 1.71	5.06 ± 1.59
Week 37	6.20 ± 1.17	4.31 ± 2.57	4.20 ± 0.98	1.69 ± 0.97
Week 38	1.87 ± 2.07	0.46 ± 1.27	1.60 ± 2.91	1.25 ± 1.16
Week 39	3.00 ± 1.18	6.46 ± 1.88	4.70 ± 2.00	1.69 ± 1.08
Week 40	9.13 ± 1.32[c]	6.15 ± 1.29	-0.50 ± 2.14[a]	3.38 ± 0.82

Values are reported as mean body weight gain per rat in grams ± SEM and data were analyzed by Two-way ANOVA with repeated measures and Tukey's post-hoc test with time considered a variable ($P \leq 0.05$). Treatment groups were significantly different from a = Water, b = C-PAC, c = Reflux and d = C-PAC+Reflux.

Supplementary Table 3. Mean animal food consumption in grams by week of study.				
	**Water**	**C-PAC**	**Reflux**	**C-PAC+Reflux**
Week 1	166.07 ± 3.80[c,d]	173.42 ± 6.13[c,d]	132.43 ± 7.25[a,b]	132.55 ± 4.75[a,b]
Week 2	183.14 ± 6.00[c,d]	177.96 ± 3.10[c,d]	153.99 ± 5.72[a,b]	151.75 ± 5.30[a,b]
Week 3	175.21 ± 9.52	175.56 ± 4.91	165.49 ± 6.02	158.60 ± 7.38
Week 4	189.37 ± 6.40[c,d]	175.34 ± 5.21	166.73 ± 5.11[a]	163.14 ± 3.59[a]
Week 5	181.98 ± 4.20[d]	176.59 ± 3.84	164.84 ± 4.92	162.70 ± 4.05[a]
Week 6	179.36 ± 5.14[d]	174.74 ± 2.55[d]	161.82 ± 4.66	157.12 ± 4.30[a,b]
Week 7	177.11 ± 4.84[d]	173.20 ± 4.03[d]	160.49 ± 3.53	154.35 ± 3.80[a,b]
Week 8	172.81 ± 5.44[d]	171.00 ± 5.86[d]	156.52 ± 4.06	152.31 ± 3.77[a,b]
Week 9	171.51 ± 4.05[c,d]	170.23 ± 5.19[d]	153.71 ± 4.63[a]	152.71 ± 3.16[a,b]
Week 10	177.25 ± 5.15[c,d]	176.37 ± 5.18[c,d]	152.24 ± 3.61[a,b]	153.14 ± 3.37[a,b]
Week 11	176.11 ± 3.84[c,d]	177.64 ± 4.59[c,d]	149.76 ± 4.16[a,b]	147.94 ± 3.16[a,b]
Week 12	172.79 ± 4.27[c,d]	172.10 ± 5.38[c,d]	149.32 ± 4.71[a,b]	147.55 ± 2.89[a,b]
Week 13	168.55 ± 4.03[c,d]	170.30 ± 5.55[c,d]	145.63 ± 4.37[a,b]	149.94 ± 3.20[a,b]
Week 14	169.45 ± 4.74[c,d]	173.04 ± 6.25[c,d]	143.99 ± 5.42[a,b]	148.80 ± 2.74[a,b]
Week 15	167.46 ± 2.99[c,d]	167.07 ± 5.46[c,d]	145.62 ± 4.08[a,b]	146.34 ± 5.25[a,b]
Week 16	168.42 ± 4.17[c,d]	169.03 ± 5.39[c,d]	140.53 ± 4.16[a,b]	145.46 ± 3.89[a,b]
Week 17	166.43 ± 2.88[c,d]	168.56 ± 4.71[c,d]	140.99 ± 5.69[a,b]	145.75 ± 3.85[a,b]
Week 18	164.79 ± 2.76[c,d]	170.13 ± 5.63[c,d]	134.88 ± 6.51[a,b]	143.93 ± 3.24[a,b]
Week 19	166.16 ± 3.07[c,d]	164.53 ± 5.84[c,d]	139.84 ± 4.24[a,b]	144.82 ± 2.74[a,b]
Week 20	164.59 ± 4.84[c,d]	167.89 ± 6.22[c,d]	139.64 ± 4.94[a,b]	143.38 ± 2.66[a,b]
Week 21	164.83 ± 4.61[c,d]	174.10 ± 7.83[c,d]	140.26 ± 4.94[a,b]	137.10 ± 3.22[a,b]
Week 22	168.25 ± 3.17[c,d]	177.03 ± 8.44[c,d]	141.29 ± 5.85[a,b]	139.67 ± 2.46[a,b]
Week 23	166.09 ± 3.96[c,d]	174.61 ± 6.97[c,d]	144.01 ± 6.87[a,b]	142.13 ± 3.06[a,b]
Week 24	164.03 ± 2.42[c,d]	173.86 ± 7.62[c,d]	144.60 ± 5.59[a,b]	142.55 ± 3.00[a,b]
Week 25	162.34 ± 2.50[c,d]	174.04 ± 6.05[c,d]	140.72 ± 4.72[a,b]	140.84 ± 2.64[a,b]
Week 26	163.24 ± 3.12[c,d]	173.47 ± 6.27[c,d]	141.79 ± 5.23[a,b]	139.25 ± 2.76[a,b]
Week 27	166.56 ± 3.46[c,d]	165.01 ± 8.22[c,d]	143.13 ± 4.82[a,b]	140.13 ± 2.48[a,b]
Week 28	163.83 ± 3.62[c,d]	172.24 ± 6.77[c,d]	143.58 ± 4.96[a,b]	138.48 ± 3.22[a,b]
Week 29	165.51 ± 3.05[c,d]	171.14 ± 6.80[c,d]	142.23 ± 8.31[a,b]	135.50 ± 2.27[a,b]
Week 30	166.09 ± 2.67[c,d]	167.56 ± 4.13[c,d]	138.08 ± 5.87[a,b]	137.82 ± 2.12[a,b]
Week 31	159.16 ± 7.60[c,d]	168.31 ± 5.63[c,d]	141.58 ± 5.40[a,b]	139.06 ± 3.75[a,b]
Week 32	157.52 ± 4.74[c,d]	169.91 ± 7.16[c,d]	144.29 ± 4.59[a,b]	139.25 ± 4.15[a,b]
Week 33	162.87 ± 4.24[c,d]	169.64 ± 6.16[c,d]	142.36 ± 5.75[a,b]	137.28 ± 2.94[a,b]
Week 34	163.59 ± 3.69[c,d]	165.63 ± 7.23[c,d]	141.83 ± 4.59[a,b]	139.15 ± 2.98[a,b]
Week 35	166.38 ± 3.44[c,d]	162.59 ± 8.52[c,d]	141.89 ± 4.29[a,b]	137.40 ± 3.06[a,b]
Week 36	163.73 ± 4.70[c,d]	166.27 ± 8.65[c,d]	141.73 ± 5.34[a,b]	139.42 ± 3.84[a,b]
Week 37	168.77 ± 3.45[c,d]	170.77 ± 9.26[c,d]	140.88 ± 4.84[a,b]	137.88 ± 3.49[a,b]
Week 38	169.09 ± 4.61[c,d]	158.76 ± 6.68[c,d]	138.52 ± 6.54[a,b]	137.96 ± 3.59[a,b]
Week 39	164.06 ± 4.84[c,d]	163.43 ± 5.80[c,d]	140.86 ± 5.85[a,b]	138.60 ± 2.85[a,b]
Week 40	168.19 ± 3.48[c,d]	167.94 ± 4.54[c,d]	140.78 ± 6.01[a,b]	139.25 ± 3.38[a,b]

Values are reported as mean food consumption per rat in grams ± SEM and data were analyzed by Two-way ANOVA with repeated measures and Tukey's post-hoc test with time considered a variable ($P \leq 0.05$). Treatment groups were significantly different from a = Water, b = C-PAC, c = Reflux and d = C-PAC+Reflux.

Supplementary Table 4. Mean water consumption (milliliters) by week of study.

	Water	C-PAC	Reflux	C-PAC+Reflux
Week 1	126.24 ± 7.56	134.59 ± 7.29	116.08 ± 9.36	127.94 ± 11.46
Week 2	151.50 ± 10.41	164.26 ± 15.66	172.36 ± 11.66	144.19 ± 6.07
Week 3	183.11 ± 11.67	170.91 ± 8.70	186.10 ± 8.63	170.73 ± 16.93
Week 4	176.64 ± 11.71	175.06 ± 14.07	187.86 ± 15.84	160.25 ± 15.82
Week 5	184.40 ± 9.88	184.13 ± 20.68	199.20 ± 15.21	162.78 ± 16.15
Week 6	192.33 ± 10.24	193.10 ± 25.47	193.63 ± 15.52[d]	154.95 ± 17.28[c]
Week 7	182.46 ± 9.54	178.06 ± 15.21	178.29 ± 13.94	151.44 ± 13.73
Week 8	194.49 ± 10.39[d]	179.04 ± 14.92	173.76 ± 14.87	150.56 ± 11.34[a]
Week 9	190.56 ± 11.88[d]	184.56 ± 19.67[d]	174.79 ± 15.10	143.19 ± 14.68[a,b]
Week 10	186.43 ± 10.11[d]	177.06 ± 19.25	163.84 ± 13.09	142.40 ± 12.02[a]
Week 11	180.65 ± 11.98[d]	176.14 ± 16.29	157.79 ± 9.54	141.05 ± 13.99[a]
Week 12	182.19 ± 11.77[d]	167.31 ± 16.40	156.90 ± 10.91	139.36 ± 11.05[a]
Week 13	175.81 ± 12.46[d]	168.14 ± 17.02	149.18 ± 7.90	132.25 ± 12.37[a]
Week 14	185.75 ± 12.98[d]	162.34 ± 11.68	152.82 ± 11.65	134.14 ± 11.07[a]
Week 15	183.98 ± 13.07[c,d]	163.81 ± 13.96	140.62 ± 8.79[a]	127.06 ± 8.96[a]
Week 16	179.46 ± 12.10[c,d]	165.70 ± 14.32	134.00 ± 7.41[a]	132.26 ± 12.70[a]
Week 17	177.30 ± 13.26[d]	151.27 ± 12.11	140.12 ± 8.72	128.83 ± 10.36[a]
Week 18	173.55 ± 9.73[c,d]	152.60 ± 10.16	129.26 ± 6.18[a]	125.86 ± 9.07[a]
Week 19	179.84 ± 9.80[c,d]	158.39 ± 12.58	132.50 ± 8.32[a]	125.99 ± 7.65[a]
Week 20	176.18 ± 9.07[c,d]	161.69 ± 9.65	133.56 ± 7.02[a]	126.27 ± 9.27[a]
Week 21	179.78 ± 11.87[c,d]	159.54 ± 9.80	134.26 ± 7.87[a]	122.00 ± 9.04[a]
Week 22	183.15 ± 11.88[c,d]	155.29 ± 7.94	133.54 ± 7.63[a]	118.71 ± 7.25[a]
Week 23	175.91 ± 11.06[c,d]	157.73 ± 9.33	131.71 ± 6.39[a]	117.22 ± 7.34[a]
Week 24	172.06 ± 8.88[c,d]	163.26 ± 11.45[d]	130.68 ± 7.29[a]	117.87 ± 7.60[a,b]
Week 25	171.21 ± 11.41[d]	156.83 ± 10.05	131.68 ± 9.08	117.62 ± 8.55[a]
Week 26	164.61 ± 9.16[d]	152.20 ± 9.61	129.37 ± 6.94	117.47 ± 7.07[a]
Week 27	159.83 ± 9.49[d]	157.66 ± 10.48	129.84 ± 7.13	119.16 ± 8.40[a]
Week 28	168.38 ± 11.78[d]	155.54 ± 9.13	133.16 ± 7.09	119.60 ± 9.36[a]
Week 29	161.44 ± 10.44[d]	153.74 ± 9.51	127.71 ± 7.39	114.28 ± 8.52[a]
Week 30	166.30 ± 11.47[d]	145.91 ± 10.07	126.69 ± 6.61	122.31 ± 7.52[a]
Week 31	158.11 ± 11.44	151.03 ± 9.97	132.65 ± 7.62	120.40 ± 8.28
Week 32	154.04 ± 8.05	150.57 ± 9.09	128.72 ± 6.73	119.29 ± 7.28
Week 33	162.43 ± 10.87[d]	151.03 ± 7.81	127.59 ± 7.86	118.41 ± 7.90[a]
Week 34	166.61 ± 10.68[d]	149.86 ± 9.46	134.50 ± 8.69	119.48 ± 8.63[a]
Week 35	162.08 ± 10.53[d]	153.13 ± 9.97	124.23 ± 7.35	120.35 ± 7.47[a]
Week 36	166.26 ± 11.89[d]	150.99 ± 12.41	126.67 ± 7.60	121.62 ± 6.73[a]
Week 37	167.69 ± 12.37[c,d]	158.71 ± 13.79	123.70 ± 6.84[a]	119.05 ± 6.99[a]
Week 38	161.55 ± 11.66[d]	153.10 ± 11.77	130.80 ± 9.82	117.88 ± 7.16[a]
Week 39	156.55 ± 12.75[d]	139.41 ± 10.13	129.88 ± 8.55	116.32 ± 8.50[a]
Week 40	156.69 ± 13.44[d]	152.27 ± 13.19	120.68 ± 7.65	117.40 ± 7.47[a]

Values are reported as mean water consumption per rat in milliliters ± SEM and data were analyzed by Two-way ANOVA with repeated measures and Tukey's post-hoc test with time considered a variable ($P \leq 0.05$). Treatment groups were significantly different from a = Water, b = C-PAC, c = Reflux and d = C-PAC+Reflux.

Supplementary Table 5. Frequency of gut microbiome species by treatment group in a rat model of reflux-induced EAC.								
**Detected Species**	**Water**	**C-PAC**	**Reflux**	**C-PAC + Reflux**	**Trend Test (*P*-value)**	**Gram Status**	**Family**	**Phylum**
Alistipes onderdonkii	0.00%	0.00%	28.60%	0.00%	0.0947	-	*Rikenellaceae*	*Bacteroidetes*
Bacteroides dorei	0.00%	0.00%	28.60%	0.00%	0.0947	-	*Bacteroidaceae*	*Bacteroidetes*
Bacteroides sp. 1_1_6	28.60%	0.00%	71.40%	0.00%[b]	0.0052	-	*Bacteroidaceae*	*Bacteroidetes*
Bacteroides sp. 4_1_36	28.60%	0.00%	100.00%[a]	62.50%	0.0020	-	*Bacteroidaceae*	*Bacteroidetes*
Bacteroides thetaiotamicron	71.40%	66.70%	0.00%[a]	12.50%	0.0069	-	*Bacteroidaceae*	*Bacteroidetes*
Bifidobacterium animalis	0.00%	16.70%	85.70%[a]	25.00%[b]	0.0039	+	*Bifidobacteriaceae*	*Actinobacteria*
Citrobacter rodentium	0.00%	0.00%	85.70%[a]	50.00%	0.0014	-	*Enterobacteriaceae*	*Proteobacteria*
Clostridium hathewayi	0.00%	0.00%	28.60%	0.00%	0.0947	-	*Clostridiaceae*	*Firmicutes*
Clostridium perfringens	14.30%	16.70%	71.40%	25.00%	0.0778	+	*Clostridiaceae*	*Firmicutes*
Clostridium phage vB-CpeS-CP51	14.30%	16.70%	71.40%	25.00%	0.0778	N/A	*Siphoviridae*	*Uroviricota*
Clostridium sp. JCC	100.00%	100.00%	57.10%	25.00%	0.0041	+	*Clostridiaceae*	*Firmicutes*
Collinsella aerofaciens	0.00%	0.00%	57.10% [a]	0.00% [b]	0.0029	+	*Coriobacteriaceae*	*Actinobacteria*
Dorea sp. 5-2	57.10%	0.00%	28.60%	0.00%	0.0252	+	*Lachnospiraceae*	*Firmicutes*
Enterobacteria phage lambda	14.30%	0.00%	57.10%	12.50%	0.0568	N/A	*Siphoviridae*	*Uroviricota*
Enterococcus faecalis	0.00%	0.00%	100.00% [a]	75.00%	0.0001	+	*Enterococcaceae*	*Firmicutes*
Escherichia coli	57.10%	0.00%	100.00%	75.00%	0.0023	-	*Enterobacteriaceae*	*Proteobacteria*
Eubacterium limosum	0.00%	50.00%	42.90%	0.00%	0.0305	+	*Eubacteriaceae*	*Firmicutes*
Eubacterium sp. 14-2	85.70%	50.00%	0.00% [a]	12.50%	0.0030	+	*Eubacteriaceae*	*Firmicutes*
Lachnospiraceae bacterium 1_4_56FAA	28.60%	83.30%	14.30%	50.00%	0.0689	+	*Lachnospiraceae*	*Firmicutes*
Lachnospiraceae bacterium 10-1	0.00%	16.70%	28.60%	75.00%	0.0131	+	*Lachnospiraceae*	*Firmicutes*
Lactobacillus johnsonii	57.10%	100.00%	14.30%	75.00%[b]	0.0121	+	*Lactobacillaceae*	*Firmicutes*
Lactobacillus sp. ASF360	0.00%	0.00%	85.70%[a]	25.00%[b]	0.0009	+	*Lactobacillaceae*	*Firmicutes*
Lactococcus lactis	71.40%	100.00%	100.00%	37.50%[b]	0.0156	+	*Streptococcaceae*	*Firmicutes*
Odoribacter laneus	28.60%	0.00%	85.70%	50.00%	0.0148	-	*Odoribacteraceae*	*Bacteroidetes*
Parabacteroides distasonis	71.40%	100.00%	57.10%	100.00%	0.0868	-	*Tannerellaceae*	*Bacteroidetes*
Parasutterella excrementihomonis	14.30%	0.00%	57.10%	25.00%	0.0985	-	*Sutterellaceae*	*Proteobacteria*
Proteus mirabilis	0.00%	0.00%	57.10%[a]	0.00%[b]	0.0029	-	*Morganellaceae*	*Proteobacteria*
Rikenella microfusus	28.60%	0.00%	85.70%	37.50%	0.0144	-	*Rikenellaceae*	*Bacteroidetes*
Streptococcus macedonicus	57.10%	83.30%	14.30%	37.50%	0.0795	+	*Streptococcaceae*	*Firmicutes*
Streptococcus mutans	0.00%	50.00%	85.70%[a]	50.00%	0.0149	+	*Streptococcaceae*	*Firmicutes*
Streptococcus parasanguinis	14.30%	33.30%	85.70%[a]	37.50%	0.0481	+	*Streptococcaceae*	*Firmicutes*
Subdoligranulum sp. 4_3_54A2FAA	57.10%	83.30%	14.30%	25.00%	0.0466	-	*Ruminococcaceae*	*Firmicutes*
Yersina phage L-413C	0.00%	0.00%	42.90%	0.00%	0.0179	N/A	*Myoviridae*	*Uroviricota*

[a] Significantly different from water or [b] Significantly different from reflux with *P*-value determined by Fisher's exact test. The Chi square test was utilized as a test for trend with *P*-value reported. C-PAC, cranberry proanthocyanidins; EAC, esophageal adenocarcinoma; N/A, not applicable.

Supplementary Table 6. Frequency of gut microbiome families by treatment group in a rat model of reflux-induced EAC.

Detected Family	Water	C-PAC	Reflux	C-PAC + Reflux	Trend Test (*P*-value)	Gram Status	Phylum
Bifidobacteriaceae	42.86%	100.00%	100.00%[a]	50.00%	0.0203	+	*Actinobacteria*
Clostridiaceae	100.00%	100.00%	100.00%	37.50%[b]	0.0016	+/-	*Firmicutes*
Coriobacteriaceae	100.00%	100.00%	85.71%	50.00%	0.0364	+	*Actinobacteria*
Deferribacteraceae	71.43%	33.33%	14.29%	12.50%	0.0603	-	*Deferribacteres*
Enterobacteriaceae	57.14%	0.00%	100.00%	87.50%	0.0008	-	*Proteobacteria*
Enterococcaceae	14.29%	16.67%	100.00%[a]	75.00%	0.0017	+	*Firmicutes*
Eubacteriaceae	100.00%	100.00%	85.71%	37.50%	0.0082	+	*Firmicutes*
Microviridae	28.57%	0.00%	0.00%	0.00%	0.0912	N/A	*Phixviricota*
Myoviridae	0.00%	0.00%	71.43%[a]	50.00%	0.0065	N/A	*Uroviricota*
Siphoviridae	28.57%	16.67%	85.71%	37.50%	0.0549	N/A	*Uroviricota*
Sutterellaceae	14.29%	0.00%	57.14%	25.00%	0.0985	-	*Proteobacteria*

[a] Significantly different from water or [b] Significantly different from reflux with *P*-value determined by Fisher's exact test. The Chi square test was utilized as a test for trend with *P*-value reported. C-PAC, cranberry proanthocyanidins; EAC, esophageal adenocarcinoma; N/A, not applicable.

Supplementary Table 7. Pathway Maps (n=45) up-regulated by reflux and directly reversed by C-PAC (n=140 metabolites; FDR <0.05).

Pathway Map	FDR	Metabolites in Data
Aminoacyl-tRNA biosynthesis in mitochondrion	1.897E-17	L-Valine intracellular anatomical structure, Glycine cytoplasm, L-Cysteine intracellular anatomical structure, L-Alanine intracellular anatomical structure, L-Glutamic acid cytoplasm, L-Tryptophan intracellular anatomical structure, L-Methionine intracellular anatomical structure, L-Serine cytoplasm, L-Phenylalanine cytoplasm, L-Threonine cytoplasm, L-Tyrosine intracellular anatomical structure, L-Leucine cytoplasm, L-Arginine cytoplasm, L-Proline cytoplasm, L-Isoleucine intracellular anatomical structure, L-Aspartic acid cytoplasm, L-Histidine cytoplasm, L-Lysine cytoplasm
Aminoacyl-tRNA biosynthesis in cytoplasm	3.064E-16	L-Valine intracellular anatomical structure, Glycine cytoplasm, L-Cysteine intracellular anatomical structure, L-Alanine intracellular anatomical structure, L-Glutamic acid cytoplasm, L-Tryptophan intracellular anatomical structure, L-Methionine intracellular anatomical structure, L-Serine cytoplasm, L-Phenylalanine cytoplasm, L-Threonine cytoplasm, L-Tyrosine intracellular anatomical structure, L-Leucine cytoplasm, L-Arginine cytoplasm, L-Proline cytoplasm, L-Isoleucine intracellular anatomical structure, L-Aspartic acid cytoplasm, L-Histidine cytoplasm, L-Lysine cytoplasm
Glycine and L-Serine metabolism	1.292E-12	Glycine mitochondrial matrix, Glycine cytoplasm, L-Alanine intracellular anatomical structure, Betaine cytoplasm, Betaine cytosol, L-Glutamic acid cytoplasm, Betaine mitochondrial matrix, L-Methionine intracellular anatomical structure, L-Serine cytoplasm, L-Serine mitochondrial matrix, Glycine extracellular region, S-Adenosyl-L-methionine cytoplasm, L-Serine cytosol, N-Methyl-glycine intracellular anatomical structure, Glycine cytosol, L-Serine extracellular region, Betaine aldehyde intracellular anatomical structure
Metabolism of L-cysteine, D-cysteine and L-cystine	7.610E-10	L-alpha-Aminobutyric acid intracellular anatomical structure, Glycine cytoplasm, 3-Sulfino-L-alanine mitochondrial matrix, L-Cysteine intracellular anatomical structure, 3-Sulfino-L-alanine cytoplasm, L-Cystine cytosol, L-Glutamic acid cytoplasm, L-Cystine extracellular region, L-Serine cytoplasm, L-Cystathionine intracellular anatomical structure, L-Cysteine extracellular region, L-Cystine lysosome, Glutathione intracellular anatomical structure, L-Cystine cytoplasm
Nociception_Pro-nociceptive action of Nociceptin in spinal cord at low doses	6.767E-09	L-Glutamic acid cytosol, Glycine extracellular region, L-Arginine cytoplasm, L-Citrulline cytoplasm, L-Glutamic acid extracellular region, Prostaglandin E2 extracellular region, Histamine intracellular anatomical structure, Glycine cytosol, Histamine extracellular region
Stem cells_Excitotoxicity of Glutamate in glioblastoma	2.244E-08	L-Cysteine intracellular anatomical structure, 3-Sulfino-L-alanine cytoplasm, L-Cystine cytosol, L-Glutamic acid cytoplasm, L-Cystine extracellular region, L-Glutamic acid cytosol, L-Glutamic acid extracellular region, Glutathione intracellular anatomical structure, L-Cystine cytoplasm
Renal secretion of organic electrolytes / Rodent version	1.297E-07	Succinic acid cytosol, Uric acid extracellular region, L-Methionine intracellular anatomical structure, L-Methionine extracellular region, Succinic acid extracellular region, Histamine intracellular anatomical structure, Glutathione intracellular anatomical structure, Histamine extracellular region, Uric acid intracellular anatomical structure, Glutathione extracellular region
Myeloid-derived suppressor cells and M2 macrophages in cancer	4.468E-07	L-Cysteine intracellular anatomical structure, L-Tryptophan extracellular region, L-Cystine extracellular region, L-Tryptophan intracellular anatomical structure, L-Arginine cytoplasm, L-Arginine cytosol, Prostaglandin E2 extracellular region, L-Arginine extracellular region, L-Cystine cytoplasm
Metabolism in pancreatic cancer cells	5.249E-07	Glycine cytoplasm, L-Cysteine intracellular anatomical structure, L-Cystine cytosol, L-Glutamic acid cytoplasm, L-Cystine extracellular region, L-Glutamic acid cytosol, L-Glutamic acid extracellular region, Glutathione intracellular anatomical structure, L-Cystine cytoplasm
Signal transduction_Amino acid-dependent mTORC1 activation	1.291E-05	L-Leucine cytosol, L-Leucine extracellular region, L-Glutamic acid cytoplasm, L-Leucine lysosome, L-Arginine lysosome, L-Leucine cytoplasm, L-Arginine cytoplasm, L-Arginine cytosol, L-Arginine extracellular region

L-Alanine and L-cysteine metabolism	1.549E-04	Glycine cytoplasm, L-Cysteine intracellular anatomical structure, L-Alanine intracellular anatomical structure, 3-Sulfino-L-alanine cytoplasm, L-Glutamic acid cytoplasm, L-Cystathionine intracellular anatomical structure
Neurophysiological process_PGE2-induced pain processing	1.615E-04	Prostaglandin E2 intracellular anatomical structure, L-Glutamic acid cytosol, Glycine extracellular region, L-Glutamic acid extracellular region, Prostaglandin E2 extracellular region, Glycine cytosol
Urea cycle	6.005E-04	L-Arginine cytoplasm, L-Citrulline cytoplasm, L-Citrulline cytosol, L-Citrulline mitochondrial matrix, L-Aspartic acid cytoplasm
IgE-dependent production of pro-inflammatory mediators by neutrophils in asthma	7.308E-04	Prostaglandin E2 intracellular anatomical structure, Prostaglandin E2 extracellular region, Histamine intracellular anatomical structure, Histamine extracellular region, L-Histidine cytoplasm
Histidine-glutamate-glutamine metabolism	8.491E-04	Succinic acid cytoplasm, L-Alanine intracellular anatomical structure, L-Glutamic acid cytoplasm, L-Proline cytoplasm, Histamine intracellular anatomical structure, L-Aspartic acid cytoplasm, L-Histidine cytoplasm
Disruption of methionine metabolism in induction and progression of HCC	9.785E-04	L-Methionine intracellular anatomical structure, L-Serine cytoplasm, L-Cystathionine intracellular anatomical structure, Methylthioadenosine intracellular anatomical structure, S-Adenosyl-L-methionine cytoplasm
Sulfur metabolism	9.785E-04	L-Cysteine intracellular anatomical structure, 3-Sulfino-L-alanine cytoplasm, L-Serine cytoplasm, L-Cystathionine intracellular anatomical structure, Glutathione intracellular anatomical structure
Taurine and hypotaurine metabolism	9.785E-04	Taurocholic acid intracellular anatomical structure, L-Cysteine intracellular anatomical structure, 3-Sulfino-L-alanine cytoplasm, Glutathione intracellular anatomical structure
Glutathione metabolism	1.121E-03	Glycine cytoplasm, L-Cysteine intracellular anatomical structure, L-Glutamic acid cytoplasm, Cys-Gly intracellular anatomical structure, 5-Oxo-L-proline intracellular anatomical structure, Glutathione intracellular anatomical structure
L-Phenylalanine metabolism	1.345E-03	L-Phenylalanine extracellular region, L-Phenylalanine cytoplasm, L-Phenylalanine cytosol, L-Phenylalanine lysosome, L-Tyrosine intracellular anatomical structure, Phenylpyruvic acid intracellular anatomical structure
Neurophysiological process_nNOS signaling in neuronal synapses	2.956E-03	L-Glutamic acid cytosol, L-Arginine cytoplasm, L-Citrulline cytoplasm, L-Glutamic acid extracellular region
Signal transduction_Production and main functions of biologically active prostaglandins and Thromboxane A2	3.930E-03	Prostaglandin E2 intracellular anatomical structure, 15D-PGJ2 intracellular anatomical structure, Prostaglandin F2 alpha intracellular anatomical structure, Prostaglandin F2 alpha extracellular region, Prostaglandin E2 extracellular region
EGFR signaling pathway in colorectal cancer	3.958E-03	Glycodeoxycholate extracellular region, Prostaglandin E2 intracellular anatomical structure, Taurodeoxycholic acid extracellular region, Taurolithocholic acid extracellular region, Prostaglandin E2 extracellular region
L-Lysine metabolism	3.958E-03	L-Lysine extracellular region, L-Glutamic acid cytoplasm, L-Lysine cytosol, L-Lysine mitochondrial matrix, L-Lysine cytoplasm, 5-Hydroxy-L-lysine intracellular anatomical structure
Beta-alanine metabolism	4.566E-03	Uracil intracellular anatomical structure, S-Adenosyl-L-methionine cytoplasm, L-Aspartic acid cytoplasm, L-Histidine cytoplasm
Aspartate and asparagine metabolism	8.937E-03	L-Glutamic acid cytoplasm, L-Arginine cytoplasm, L-Citrulline cytoplasm, N-Carbamoylaspartate intracellular anatomical structure, L-Aspartic acid cytoplasm
Metabolism of L-Proline and derivatives	9.041E-03	L-Proline mitochondrial matrix, L-Proline cytosol, L-Glutamic acid cytoplasm, L-Proline extracellular region, L-Proline cytoplasm
Leucine, isoleucine and valine metabolism	9.041E-03	L-Valine intracellular anatomical structure, L-Glutamic acid cytoplasm, L-Leucine cytoplasm, L-Isoleucine intracellular anatomical structure
L-Arginine metabolism	1.147E-02	L-Arginine cytoplasm, L-Citrulline cytoplasm, S-Adenosyl-L-methionine cytoplasm, L-Aspartic acid cytoplasm, Spermidine intracellular anatomical structure

L-Threonine metabolism	1.493E-02	L-Threonine cytoplasm, L-Threonine mitochondrial matrix, L-Threonine extracellular region, L-Threonine cytosol
Stem cells_mGluR3 signaling in glioblastoma stem cells	1.673E-02	L-Cystine cytosol, L-Cystine extracellular region, L-Glutamic acid cytosol, L-Glutamic acid extracellular region
Histamine metabolism	2.200E-02	N'-Acetylhistamine intracellular anatomical structure, Histamine intracellular anatomical structure, L-Histidine cytoplasm
L-Methionine metabolism	2.432E-02	L-Methionine intracellular anatomical structure, L-Methionine extracellular region, Methylthioadenosine intracellular anatomical structure, S-Adenosyl-L-methionine cytoplasm
Reactive oxygen and nitrogen species production in eosinophils in asthma	2.432E-02	L-Tyrosine intracellular anatomical structure, L-Arginine cytoplasm, L-Citrulline cytoplasm, Histamine extracellular region
Aminosugar metabolism	2.512E-02	CMP-N-Acetylneuraminic acid cytoplasm, L-Glutamic acid cytoplasm, CMP-N-Acetylneuraminic acid Golgi lumen, CMP-N-Acetylneuraminic acid cytosol
Muscle contraction_Oxytocin signaling in uterus and mammary gland	2.752E-02	Prostaglandin E2 intracellular anatomical structure, Prostaglandin F2 alpha intracellular anatomical structure, Prostaglandin F2 alpha extracellular region, Prostaglandin E2 extracellular region
Polyamine metabolism	3.004E-02	L-Arginine cytoplasm, Methylthioadenosine intracellular anatomical structure, S-Adenosyl-L-methionine cytoplasm, Spermidine intracellular anatomical structure
Muscle contraction_Regulation of eNOS activity in endothelial cells	3.093E-02	L-Arginine cytoplasm, L-Citrulline cytoplasm, L-Arginine cytosol, L-Arginine extracellular region
Ceramide and ganglioside metabolism and neuropathic pain	3.847E-02	Sphinganine intracellular anatomical structure, L-Glutamic acid cytoplasm, L-Serine cytoplasm, Prostaglandin E2 intracellular anatomical structure, L-Glutamic acid cytosol, L-Glutamic acid extracellular region
Endothelial cell apoptosis in COPD	3.930E-02	L-Arginine cytoplasm, L-Citrulline cytoplasm, Glutathione intracellular anatomical structure
G-protein signaling_Rac2 regulation pathway	3.930E-02	Inositol 1-phosphate intracellular anatomical structure, L-Arginine cytoplasm, L-Citrulline cytoplasm
A shift in alveolar macrophage phenotype in COPD	4.048E-02	N-Acetyl-D-glucosamine extracellular region, L-Arginine cytoplasm, L-Citrulline cytoplasm
Deregulation of PSD-95-dependent signaling in Huntington's disease	4.048E-02	L-Arginine cytoplasm, L-Citrulline cytoplasm, L-Glutamic acid extracellular region
Immune response_Distinct metabolic pathways in naive and effector CD8+ T cells	4.241E-02	L-Leucine cytosol, L-Leucine extracellular region, L-Glutamic acid cytoplasm, L-Malic acid cytoplasm
De novo IMP biosynthesis	4.485E-02	Glycine cytoplasm, L-Glutamic acid cytoplasm, L-Aspartic acid cytoplasm

Significant pathway maps determined in Metacore with a false discovery rate (FDR) set at 0.05. C-PAC, cranberry proanthocyanidins.

Supplementary Table 8. Pathway Maps (n=21) down-regulated by reflux and directly reversed by C-PAC (n=60 metabolites; FDR ≤0.05).

Pathway Map	FDR	Metabolites in Data
Regulation of lipid metabolism_PPAR regulation of lipid metabolism	2.534E-05	Acyl-L-carnitine mitochondrial matrix, L-Carnitine cytoplasm, Acyl-L-carnitine cytosol, L-Carnitine mitochondrial matrix, L-Carnitine cytosol
Sphingolipid metabolism	3.649E-05	N-Acylsphingosine endoplasmic reticulum membrane, N-Acylsphingosine cytoplasm, Sphingomyelin intracellular anatomical structure, Phosphatidylcholine intracellular anatomical structure, Lactosylceramide intracellular anatomical structure, N-Acylsphingosine Golgi membrane
L-Carnitine biosynthesis	3.057E-03	L-Carnitine cytoplasm, L-Carnitine extracellular region, L-Carnitine cytosol
Taurine and hypotaurine metabolism	3.576E-02	Hypotaurine intracellular anatomical structure, Taurine intracellular anatomical structure
L-Lysine metabolism	3.576E-02	L-Acetylcarnitine mitochondrial matrix, L-Acetylcarnitine cytoplasm, L-Acetylcarnitine cytosol
L-Tryptophan metabolism (part 2)	3.576E-02	L-Acetylcarnitine mitochondrial matrix, L-Acetylcarnitine cytoplasm, L-Acetylcarnitine cytosol
Cholesterol and Sphingolipid transport / Influx to the early endosome in lung (normal and CF)	3.576E-02	Sphingomyelin intracellular anatomical structure, Sphingomyelin plasma membrane
Saturated fatty acids synthesis to hexadecanoic acid	3.576E-02	Malonic acid cytosol, Malonic acid mitochondrial matrix, Malonic acid cytoplasm
Acetylcholine biosynthesis and metabolism	3.576E-02	Lysophosphatidylcholine intracellular anatomical structure, Phosphatidylcholine intracellular anatomical structure
Phospholipid metabolism p.3	3.576E-02	Lysophosphatidylcholine intracellular anatomical structure, Phosphatidylcholine intracellular anatomical structure
Pentose phosphate pathway	3.576E-02	D-Ribulose-5-phosphate intracellular anatomical structure, D-Sedoheptulose-7-phosphate intracellular anatomical structure
Action of lithium on synaptic transmission and autophagy	3.576E-02	Myo-Inositol extracellular region, Myo-Inositol intracellular anatomical structure
Beta-alanine metabolism	3.576E-02	Carnosine cytoplasm, Anserine intracellular anatomical structure
Cholesterol and Sphingolipid transport / Transport from Golgi and ER to the apical membrane (normal and CF)	3.657E-02	N-Acylsphingosine cytoplasm, Sphingomyelin intracellular anatomical structure
Role of glycosphingolipids in inhibition of Insulin signaling	3.657E-02	N-Acylsphingosine cytoplasm, Lactosylceramide intracellular anatomical structure
Apoptosis and survival_Ceramides signaling pathway	3.657E-02	N-Acylsphingosine cytoplasm, Sphingomyelin intracellular anatomical structure
Role of TNF-alpha in type 2 diabetes in skeletal muscle cells	3.657E-02	Acyl-L-carnitine mitochondrial matrix, L-Carnitine cytoplasm
Transcription_FXR-regulated cholesterol and bile acids cellular transport	3.657E-02	Phosphatidylcholine extracellular region, Phosphatidylcholine intracellular anatomical structure
Apoptosis and survival_TNF-alpha-induced Caspase-8 signaling	3.657E-02	N-Acylsphingosine cytoplasm, Sphingomyelin intracellular anatomical structure
Regulation of GSK3 beta in bipolar disorder	3.960E-02	N-Acylsphingosine cytoplasm, Sphingomyelin intracellular anatomical structure
Ceramide and ganglioside metabolism and neuropathic pain	5.407E-02	N-Acylsphingosine cytoplasm, Sphingomyelin intracellular anatomical structure, Lactosylceramide intracellular anatomical structure

Significant pathway maps determined in Metacore with a false discovery rate (FDR) set at 0.05. C-PAC, cranberry proanthocyanidins.

Supplementary Table 9. Metabolic Networks (n=29) up-regulated by reflux and directly reversed by C-PAC (n=140 metabolites; FDR <0.05).		
**Metabolic Network**	**FDR**	**Metabolites in Data**
Glutamic acid pathways and transport	6.522E-12	L-Valine intracellular anatomical structure, L-Cysteine intracellular anatomical structure, L-Leucine cytosol, L-Tryptophan extracellular region, L-Tyrosine extracellular region, L-Leucine extracellular region, L-Isoleucine extracellular region, L-Cystine cytosol, L-Glutamic acid cytoplasm, L-Cystine extracellular region, L-Tryptophan intracellular anatomical structure, L-Glutamic acid mitochondrial matrix, L-Glutamic acid cytosol, L-Valine extracellular region, L-Tyrosine intracellular anatomical structure, L-Leucine cytoplasm, L-Glutamic acid cytoplasmic vesicle, L-Cysteine extracellular region, L-Glutamic acid extracellular region, L-Isoleucine intracellular anatomical structure, L-Cystine cytoplasm
Lipid metabolism_Glycosphingolipid metabolism	1.158E-11	Succinic acid cytoplasm, Glycine cytoplasm, L-Cysteine intracellular anatomical structure, L-Alanine intracellular anatomical structure, Betaine cytoplasm, 3-Sulfino-L-alanine cytoplasm, L-Alanine extracellular region, L-Glutamic acid cytoplasm, L-Methionine intracellular anatomical structure, L-Serine cytoplasm, L-Methionine extracellular region, L-Proline extracellular region, Glycine extracellular region, L-Cystathionine intracellular anatomical structure, L-Proline cytoplasm, S-Adenosyl-L-methionine cytoplasm, N-Methyl-glycine intracellular anatomical structure, L-Cysteine extracellular region, Histamine intracellular anatomical structure, Glutathione intracellular anatomical structure, L-Histidine cytoplasm, L-Serine extracellular region, Glutathione extracellular region
Aminoacid metabolism_Ala,Ser,Cys,Met,His,Pro,Gly,Glu,Gln metabolism and transport	1.158E-11	Succinic acid cytoplasm, Glycine cytoplasm, L-Cysteine intracellular anatomical structure, L-Alanine intracellular anatomical structure, Betaine cytoplasm, 3-Sulfino-L-alanine cytoplasm, L-Alanine extracellular region, L-Glutamic acid cytoplasm, L-Methionine intracellular anatomical structure, L-Serine cytoplasm, L-Methionine extracellular region, L-Proline extracellular region, Glycine extracellular region, L-Cystathionine intracellular anatomical structure, L-Proline cytoplasm, S-Adenosyl-L-methionine cytoplasm, N-Methyl-glycine intracellular anatomical structure, L-Cysteine extracellular region, Histamine intracellular anatomical structure, Glutathione intracellular anatomical structure, L-Histidine cytoplasm, L-Serine extracellular region, Glutathione extracellular region
Aminoacid metabolism_Alanine,Glycine,Cysteine metabolism and transport	5.525E-10	Glycine cytoplasm, L-Cysteine intracellular anatomical structure, L-Leucine cytosol, L-Alanine intracellular anatomical structure, Betaine cytoplasm, L-Leucine extracellular region, 3-Sulfino-L-alanine cytoplasm, L-Cystine cytosol, L-Alanine extracellular region, L-Glutamic acid cytoplasm, Glycine extracellular region, L-Cystathionine intracellular anatomical structure, S-Adenosyl-L-methionine cytoplasm, N-Methyl-glycine intracellular anatomical structure, L-Cysteine extracellular region, Glutathione intracellular anatomical structure, Betaine aldehyde intracellular anatomical structure
L-ornithine pathways and transport	1.974E-09	Succinic acid cytoplasm, Glycine cytoplasm, L-Aspartic acid cytosol, L-Leucine cytosol, L-Alanine intracellular anatomical structure, L-Leucine extracellular region, L-Glutamic acid cytoplasm, L-Aspartic acid extracellular region, L-Leucine cytoplasm, Glycine extracellular region, L-Arginine cytoplasm, L-Citrulline cytoplasm, L-Arginine cytosol, L-Glutamic acid extracellular region, L-Arginine extracellular region, Glycine cytosol, Spermidine intracellular anatomical structure
L-glutamate pathways and transport	2.221E-07	L-Valine intracellular anatomical structure, L-Cysteine intracellular anatomical structure, L-Tryptophan extracellular region, L-Tyrosine extracellular region, L-Isoleucine extracellular region, L-Glutamic acid cytoplasm, L-Tryptophan intracellular anatomical structure, L-Glutamic acid mitochondrial matrix, L-Glutamic acid cytosol, L-Valine extracellular region, L-Tyrosine

		intracellular anatomical structure, L-Glutamic acid cytoplasmic vesicle, L-Cysteine extracellular region, L-Glutamic acid extracellular region, L-Isoleucine intracellular anatomical structure
(L)-phenylalanine pathways and transport	2.276E-07	L-Phenylalanine extracellular region, L-Tryptophan extracellular region, Serotonin intracellular anatomical structure, L-Tyrosine extracellular region, L-Glutamic acid cytoplasm, L-Tryptophan intracellular anatomical structure, L-Phenylalanine cytoplasm, L-Glutamic acid cytosol, Serotonin extracellular region, Cys-Gly intracellular anatomical structure, L-Phenylalanine cytosol, L-Tyrosine intracellular anatomical structure, Phenylpyruvic acid intracellular anatomical structure, L-Glutamic acid extracellular region
L-serine pathways and transport	2.276E-07	Glycine cytoplasm, L-Cysteine intracellular anatomical structure, L-Tryptophan extracellular region, Serotonin intracellular anatomical structure, L-Tryptophan intracellular anatomical structure, L-Serine cytoplasm, Serotonin extracellular region, L-Aspartic acid extracellular region, Glycine extracellular region, L-Arginine cytoplasm, L-Cystathionine intracellular anatomical structure, L-Arginine cytosol, L-Cysteine extracellular region, L-Arginine extracellular region, L-Serine extracellular region
Glutamic acid pathway	3.572E-06	L-Valine intracellular anatomical structure, L-Cysteine intracellular anatomical structure, L-Tryptophan extracellular region, L-Tyrosine extracellular region, L-Glutamic acid cytoplasm, L-Tryptophan intracellular anatomical structure, L-Glutamic acid mitochondrial matrix, L-Glutamic acid cytosol, L-Tyrosine intracellular anatomical structure, L-Glutamic acid cytoplasmic vesicle, L-Glutamic acid extracellular region, L-Isoleucine intracellular anatomical structure
(L)-proline pathways and transport	1.411E-05	Succinic acid cytoplasm, L-Lysine extracellular region, L-Proline cytosol, L-Tryptophan extracellular region, Serotonin intracellular anatomical structure, L-Glutamic acid cytoplasm, L-Tryptophan intracellular anatomical structure, Serotonin extracellular region, L-Lysine cytosol, L-Proline extracellular region, L-Arginine cytoplasm, L-Proline cytoplasm
L-citrulline pathway	3.141E-05	L-Glutamic acid cytoplasm, L-Arginine cytoplasm, L-Citrulline cytoplasm, L-Citrulline cytosol, L-Arginine cytosol, L-Glutamic acid extracellular region, L-Arginine extracellular region, Glutathione intracellular anatomical structure, Spermidine intracellular anatomical structure, asym-Dimethylarginine intracellular anatomical structure
Aminoacid metabolism_Arginine metabolism and transport	3.622E-05	L-Aspartic acid cytosol, L-Glutamic acid cytoplasm, L-Glutamic acid mitochondrial matrix, L-Aspartic acid extracellular region, L-Arginine cytoplasm, L-Citrulline cytoplasm, L-Citrulline cytosol, L-Arginine cytosol, L-Arginine extracellular region, L-Aspartic acid cytoplasm, Spermidine intracellular anatomical structure
Tyrosine pathway	3.622E-05	L-Tryptophan extracellular region, Serotonin intracellular anatomical structure, Tyramine intracellular anatomical structure, L-Glutamic acid cytoplasm, L-Tryptophan intracellular anatomical structure, L-Phenylalanine cytoplasm, Serotonin extracellular region, L-Tyrosine intracellular anatomical structure, L-Arginine cytoplasm, L-Aspartic acid cytoplasm
Aminoacid metabolism_Asparagine, Aspartic acid metabolism and transport	3.863E-05	Uracil intracellular anatomical structure, L-Glutamic acid cytoplasm, L-Glutamic acid mitochondrial matrix, L-Glutamic acid cytosol, L-Aspartic acid extracellular region, L-Arginine cytoplasm, L-Citrulline cytoplasm, L-Glutamic acid extracellular region, N-Carbamoylaspartate intracellular anatomical structure, L-Aspartic acid cytoplasm, Spermidine intracellular anatomical structure
(L)-leucine pathways and transport	1.361E-04	L-Cysteine intracellular anatomical structure, L-Leucine cytosol, L-Leucine extracellular region, L-Cystine cytosol, L-Glutamic acid cytoplasm, L-Cystine extracellular region, L-Leucine cytoplasm, L-Cysteine extracellular region, L-Cystine cytoplasm

Aminoacid metabolism_Asparagine, Aspartic acid, Arginine metabolism and transport	3.720E-04	L-Glutamic acid cytoplasm, L-Aspartic acid extracellular region, L-Arginine cytoplasm, L-Citrulline cytoplasm, L-Arginine cytosol, N-Carbamoylaspartate intracellular anatomical structure, L-Arginine extracellular region, L-Aspartic acid cytoplasm
Vitamin, mediator and cofactor metabolism_Nitric oxide biosynthesis and transport	1.473E-03	L-Arginine cytoplasm, L-Citrulline cytoplasm, L-Citrulline cytosol, L-Citrulline mitochondrial matrix, L-Arginine cytosol, L-Arginine extracellular region
(L)-alanine pathways and transport	2.223E-03	Glycine cytoplasm, L-Tryptophan extracellular region, L-Alanine intracellular anatomical structure, Serotonin intracellular anatomical structure, L-Alanine extracellular region, L-Tryptophan intracellular anatomical structure, Serotonin extracellular region, Glycine extracellular region
Aminoacid metabolism_Tryptophan metabolism and transport	3.377E-03	L-Tryptophan extracellular region, L-Alanine intracellular anatomical structure, Serotonin intracellular anatomical structure, 5-Hydroxindoleacetic acid intracellular anatomical structure, L-Alanine extracellular region, L-Tryptophan intracellular anatomical structure, Serotonin extracellular region
Glycine pathways and transport	4.732E-03	Glycine cytoplasm, L-Cysteine intracellular anatomical structure, L-Tryptophan extracellular region, L-Alanine intracellular anatomical structure, L-Alanine extracellular region, L-Tryptophan intracellular anatomical structure, Cys-Gly intracellular anatomical structure, Glycine extracellular region
Methionine pathways and transport	4.767E-03	Betaine cytoplasm, Serotonin intracellular anatomical structure, L-Tryptophan intracellular anatomical structure, L-Methionine intracellular anatomical structure, L-Methionine extracellular region, Serotonin extracellular region, L-Arginine cytoplasm, S-Adenosyl-L-methionine cytoplasm
(L)-threonine pathways and transport	5.336E-03	L-Tryptophan extracellular region, Serotonin intracellular anatomical structure, L-Tryptophan intracellular anatomical structure, L-Threonine cytoplasm, Serotonin extracellular region, L-Threonine extracellular region, L-Threonine cytosol
Aminoacid metabolism_Tryptophan, Phenylalanine, Tyramine, Methionine metabolism and transport	7.874E-03	L-Phenylalanine extracellular region, L-Tryptophan extracellular region, L-Tyrosine extracellular region, L-Glutamic acid cytoplasm, L-Tryptophan intracellular anatomical structure, L-Phenylalanine cytoplasm, L-Phenylalanine cytosol, L-Tyrosine intracellular anatomical structure, Phenylpyruvic acid intracellular anatomical structure
Carbohydrate metabolism_TCA and tricarboxylic acid transport	8.329E-03	Succinic acid cytoplasm, Succinic acid cytosol, L-Malic acid mitochondrial matrix, L-Malic acid cytosol, L-Malic acid cytoplasm, cis-Aconitic acid intracellular anatomical structure, Succinic acid extracellular region
Vitamin, mediator and cofactor metabolism_CoA biosynthesis and transport	1.129E-02	L-Cysteine intracellular anatomical structure, L-Leucine cytosol, L-Leucine extracellular region, L-Cystine cytosol, L-Cystine extracellular region, L-Cysteine extracellular region
Aminoacid metabolism_Beta-Alanine metabolism and transport	2.155E-02	L-Aspartic acid cytosol, Uracil intracellular anatomical structure, L-Aspartic acid extracellular region, L-Aspartic acid cytoplasm
(S)-citrulline pathway	3.319E-02	L-Arginine cytoplasm, L-Citrulline cytoplasm, L-Arginine cytosol, L-Arginine extracellular region, asym-Dimethylarginine intracellular anatomical structure
D-glucuronic acid pathway	3.554E-02	Succinic acid cytoplasm, Succinic acid cytosol, L-Glutamic acid cytoplasm, L-Glutamic acid cytosol, L-Glutamic acid cytoplasmic vesicle
(L)-lysine pathways and transport	3.617E-02	L-Aspartic acid cytosol, L-Lysine extracellular region, L-Lysine cytosol, L-Aspartic acid extracellular region, Spermidine intracellular anatomical structure, L-Lysine cytoplasm

Significant metabolic networks determined in Metacore with a false discovery rate (FDR) set at 0.05. C-PAC, cranberry proanthocyanidins.

Supplementary Table 10. Metabolic Networks (n=10) down-regulated by reflux and directly reversed by C-PAC (n=60 metabolites; FDR ≤0.05).

Metabolic Network	FDR	Metabolites in Data
Aminoacid metabolism_(L)-carnitine metabolism	1.432E-04	Acyl-L-carnitine mitochondrial matrix, L-Carnitine cytoplasm, Acyl-L-carnitine cytosol, L-Carnitine mitochondrial matrix, L-Carnitine cytosol
Lauroylcarnitine pathway	1.432E-04	Acyl-L-carnitine mitochondrial matrix, L-Carnitine cytoplasm, Acyl-L-carnitine cytosol, Acyl-L-carnitine cytoplasm, L-Carnitine extracellular region, L-Carnitine cytosol
(L)-carnitine pathway	1.432E-04	L-Carnitine cytoplasm, L-Acetylcarnitine extracellular region, Acyl-L-carnitine cytoplasm, L-Acetylcarnitine cytoplasm, L-Carnitine mitochondrial matrix, L-Acetylcarnitine cytosol
1-palmitoyl-sn-glycero-3-phosphocholine pathway	8.608E-04	Taurine intracellular anatomical structure, Lysophosphatidylcholine intracellular anatomical structure, Phosphatidylcholine intracellular anatomical structure, Taurine extracellular region, 1-Palmitoyl-2-arachidonyl-3-sn-phosphatidylcholine intracellular anatomical structure
Glucosylceramide pathways and transport	8.608E-04	Taurine intracellular anatomical structure, Lysophosphatidylcholine intracellular anatomical structure, Phosphatidylcholine intracellular anatomical structure, Taurine extracellular region, 1-Palmitoyl-2-arachidonyl-3-sn-phosphatidylcholine intracellular anatomical structure
1-acyl-glycerol_3-phosphocholine pathway	9.489E-04	Taurine intracellular anatomical structure, Lysophosphatidylcholine intracellular anatomical structure, 1-Linolenoyl-3-sn-phosphatidylcholine intracellular anatomical structure, Phosphatidylcholine intracellular anatomical structure, Taurine extracellular region
Decanoylcarnitine pathway	8.177E-03	L-Carnitine cytoplasm, Acyl-L-carnitine cytoplasm, L-Carnitine extracellular region, L-Carnitine cytosol
1-oleoyl-sn-glycero-3-phosphocholine pathway	2.720E-02	Taurine intracellular anatomical structure, Lysophosphatidylcholine intracellular anatomical structure, Phosphatidylcholine intracellular anatomical structure, Taurine extracellular region
1-docosahexaenoyl-glycerol_3-phosphocholine pathway	3.119E-02	Taurine intracellular anatomical structure, Lysophosphatidylcholine intracellular anatomical structure, Phosphatidylcholine intracellular anatomical structure, Taurine extracellular region
Ceramide pathway	4.179E-02	N-Acylsphingosine cytoplasm, Sphingomyelin intracellular anatomical structure, Lactosylceramide intracellular anatomical structure

Significant metabolic networks determined in Metacore with a false discovery rate (FDR) set at 0.05. C-PAC, cranberry proanthocyanidins.

Supplementary Table 11. Process Networks (n=5) up-regulated by reflux and directly reversed by C-PAC (n=140 metabolites; FDR <0.05).

Process Network	FDR	Metabolites in Data
Translation_Elongation-Termination	1.660E-14	L-Valine intracellular anatomical structure, Glycine cytoplasm, L-Cysteine intracellular anatomical structure, L-Alanine intracellular anatomical structure, L-Glutamic acid cytoplasm, L-Tryptophan intracellular anatomical structure, L-Methionine intracellular anatomical structure, L-Serine cytoplasm, L-Phenylalanine cytoplasm, L-Threonine cytoplasm, L-Tyrosine intracellular anatomical structure, L-Leucine cytoplasm, L-Arginine cytoplasm, L-Proline cytoplasm, L-Isoleucine intracellular anatomical structure, L-Aspartic acid cytoplasm, L-Histidine cytoplasm, L-Lysine cytoplasm
Translation_Translation in mitochondria	6.162E-14	L-Valine intracellular anatomical structure, Glycine cytoplasm, L-Cysteine intracellular anatomical structure, L-Alanine intracellular anatomical structure, L-Glutamic acid cytoplasm, L-Tryptophan intracellular anatomical structure, L-Methionine intracellular anatomical structure, L-Serine cytoplasm, L-Threonine cytoplasm, L-Tyrosine intracellular anatomical structure, L-Leucine cytoplasm, L-Proline cytoplasm, L-Isoleucine intracellular anatomical structure, L-Aspartic acid cytoplasm, L-Histidine cytoplasm, L-Lysine cytoplasm
Transport_Synaptic vesicle exocytosis	1.148E-05	Serotonin intracellular anatomical structure, L-Glutamic acid cytoplasm, Serotonin extracellular region, L-Proline extracellular region, Glycine extracellular region, S-Adenosyl-L-methionine cytoplasm, L-Glutamic acid extracellular region, Histamine intracellular anatomical structure, Histamine extracellular region
Signal transduction_Oxytocin signaling	1.214E-04	Prostaglandin E2 intracellular anatomical structure, Prostaglandin F2 alpha intracellular anatomical structure, L-Arginine cytoplasm, L-Citrulline cytoplasm, Prostaglandin F2 alpha extracellular region, Prostaglandin E2 extracellular region
Muscle contraction_Nitric oxide signaling in the cardiovascular system	5.294E-02	L-Arginine cytoplasm, L-Citrulline cytoplasm, L-Arginine cytosol, L-Arginine extracellular region

Significant process networks determined in Metacore with a false discovery rate (FDR) set at 0.05. C-PAC, cranberry proanthocyanidins.

Supplementary Table 12. Process Networks (n=3) down-regulated by reflux and directly reversed by C-PAC (n=60 metabolites; FDR <0.05).

Process Network	FDR	Metabolites in Data
Signal transduction_Leptin signaling	8.766E-04	L-Carnitine cytoplasm, Acyl-L-carnitine cytoplasm, L-Carnitine mitochondrial matrix
Transport_Bile acids transport and its regulation	6.071E-03	Phosphatidylcholine extracellular region, Phosphatidylcholine intracellular anatomical structure
Signal transduction_Oxytocin signaling	6.428E-03	Lysophosphatidylcholine intracellular anatomical structure, Phosphatidylcholine intracellular anatomical structure

Significant process networks determined in Metacore with a false discovery rate (FDR) set at 0.05. C-PAC, cranberry proanthocyanidins.

Supplementary Table 13. C-PAC alters bacterial gene expression in the normal rat esophagus.

Gene	C-PAC vs Water Log_2FC	C-PAC vs Water *P*-value
Apcs[a]	-2.13	3.7E-02
Bcl10[a]	-1.20	3.0E-02
Camp[a]	-2.26	2.9E-02
Casp1[a]	-0.99	2.7E-02
Ccl4[a]	-1.36	1.0E-02
Ccl5[a]	-1.24	3.3E-02
Cxcl1[a]	-1.33	4.2E-03
Ifnb1	-1.45	3.9E-03
Il6[a]	-2.35	2.7E-03
Il12a[a]	-0.81	4.7E-02
Irf7[a]	-1.49	3.0E-02
Lyz2	-0.90	3.5E-02
Nfkbia[a]	-1.24	2.5E-02
Nod2	1.44	7.8E-03
Prdx2[a]	-1.05	1.8E-02
Prtn3	-1.99	4.4E-02
Pycard	-1.18	3.3E-02
Sugt1[a]	-0.92	3.2E-02
Ticam2[a]	-0.78	3.0E-02

[a] Denotes common in Reflux-induced EAC (table1). *P*-value determined by Student's T-test; C-PAC, cranberry proanthocyanidins; FC, fold change

Supplementary Table 14. Network list for integration of significant antimicrobial pathway genes and esophageal metabolite in C-PAC+reflux vs reflux animals.									
ID	Network	GO Processes	Total nodes	Seed notes	Pathways	*P*-value	Z Score	G Score
1	L-Glutamic acid extracellular region, MEK1/2, Serotonin extracellular region, PI3K reg class IA (p85-alpha), ERK1/2	response to oxygen-containing compound (82.0%; 4.041e-51), cellular response to oxygen-containing compound (73.0%; 1.015e-50), response to nitrogen compound (73.0%; 1.613e-49), response to organonitrogen compound (70.8%; 2.589e-48), response to endogenous stimulus (74.2%; 2.216e-45)	107	17	402	1.580E-31	48.16	550.66
2	Taurocholic acid extracellular region, Taurocholic acid intracellular anatomical structure, CDP mitochondrial matrix, Deoxyuridine extracellular region, Deoxyuridine intracellular anatomical structure	SRP-dependent cotranslational protein targeting to membrane (45.5%; 7.589e-19), cotranslational protein targeting to membrane (45.5%; 1.231e-18), viral transcription (45.5%; 4.625e-18), protein targeting to ER (45.5%; 8.111e-18), nuclear-transcribed mRNA catabolic process, nonsense-mediated decay (45.5%; 9.469e-18)	100	23	0	1.330E-50	81.49	81.49
3	Pyrin (MEFV), TNF-alpha, CARD7, Actin, CAMP	cellular response to chemical stimulus (72.3%; 1.999e-26), response to oxygen-containing compound (61.4%; 4.534e-26), response to organic substance (72.3%; 1.732e-24), positive regulation of biological process (88.0%; 4.647e-24), positive regulation of metabolic process (72.3%; 7.597e-24)	100	20	5	5.790E-39	58.46	64.71
4	TLR4, ACTB, IRF5, NF-kB, Actin	positive regulation of metabolic process (85.4%; 5.411e-39), positive regulation of macromolecule metabolic process (83.1%; 5.776e-39), response to abiotic stimulus (60.7%; 2.611e-35), response to oxygen-containing compound (68.5%; 5.600e-35), positive regulation of cellular metabolic process (77.5%; 1.693e-34)	99	21	1	3.080E-41	61.08	62.33
5	NFKBIA, CARD9, Adenine cytoplasm, IL-6, PERM	regulation of gene expression (77.5%; 3.775e-21), regulation of macromolecule metabolic process (81.2%; 2.599e-18), cellular response to chemical stimulus (62.5%; 5.393e-18), SRP-dependent cotranslational protein targeting to membrane (16.2%; 1.681e-17), cotranslational protein targeting to membrane (16.2%; 3.152e-17)	100	19	3	8.410E-37	56.1	59.85
6	IRF7, Caspase-1, NALP3, NF-kB, Caspase-8	cellular response to organic substance (80.2%; 3.494e-43), response to oxygen-containing compound (73.6%; 1.617e-41), response to organic substance (84.6%; 9.037e-40), cellular response to chemical stimulus (82.4%; 1.054e-39), positive regulation of cellular metabolic process (81.3%; 1.509e-39)	100	20	0	5.790E-39	58.46	58.46
7	TLR2, TLR9, PERM, ERK1/2, MEK1(MAP2K1)	intracellular signal transduction (67.9%; 1.661e-26), cell activation (60.7%; 4.714e-26), cellular response to oxygen-containing compound (64.3%; 1.430e-25), regulation of apoptotic process (66.1%; 4.871e-25), response to organic cyclic compound (62.5%; 5.463e-25)	100	17	1	7.410E-34	55.71	56.96
8	NF-kB1 (p105), NF-kB1 (p50), RelA (p65 NF-kB subunit), Caspase-8, PSTPIP1	positive regulation of nitrogen compound metabolic process (83.7%; 4.948e-48), response to cytokine (64.3%; 1.621e-47), response to mechanical stimulus (43.9%; 7.123e-47), response to organic substance (86.7%; 1.398e-45), positive regulation of cellular metabolic process (83.7%; 1.430e-45)	100	18	3	4.030E-34	52.04	55.79
9	NALP3, Bcl-10, NF-kB p50/p65, c-IAP1, IMP1(ZBP1)	response to oxygen-containing compound (74.7%; 7.814e-43), intracellular signal transduction (65.9%; 5.610e-40), response to organic substance (84.6%; 9.037e-40), response to organonitrogen compound (61.5%; 5.609e-38), cellular response to oxygen-containing compound (61.5%; 1.569e-37)	100	19	0	1.600E-36	55.23	55.23

10	NFKBIA, IL-12 alpha, p38alpha (MAPK14), NF-kB p65/p65, MEK1(MAP2K1)	positive regulation of cellular metabolic process (89.5%; 2.646e-52), positive regulation of metabolic process (92.6%; 8.885e-52), positive regulation of macromolecule metabolic process (89.5%; 4.417e-50), positive regulation of nitrogen compound metabolic process (85.3%; 9.260e-49), positive regulation of biosynthetic process (74.7%; 1.844e-47)	100	16	7	1.360E-29	46.22	54.97

Statistical analysis performed in Metacore with *P*-values determined using the basic formula for hypergeometric distribution. The z-score calculates the level of saturation of the networks by taking into account the database size, the number of objects in the subnetworks, and the number of objects from the user's data used to construct the given network. A higher z-Score means the network is more saturated with the user's data. The g-Score modifies the z-Score based on the number of linear canonical pathways parts in the given network. If a network has a high g-Score, it is saturated with objects from the user's dataset, and contains a large amount of canonical pathway fragments. C-PAC, cranberry proanthocyanidins.

Supplemental Table 15. PICRUSt multigroup analysis of fecal microbiomes from water, C-PAC, reflux and C-PAC+reflux treated animals (n=125 OTU ID).					
**OTU ID**	***P*-value (corrected)[a]**	**Effect size**	**KO Description**	**KEGG KO Functional Hierarchy**	**KEGG KO Pathways**
K00114	0.0299	0.5578	*exaA*; alcohol dehydrogenase (cytochrome c) [EC:1.1.2.8]	Enzymes	Metabolic Pathways; Microbial metabolism in diverse environments; Biosynthesis of secondary metabolites; Pyruvate metabolism; Glycolysis/Gluconeogenesis; Chloroalkane and chloroalkene degradation
K00124	0.0299	0.5649	*fdoH*; formate dehydrogenase iron-sulfur subunit	Metabolism	Metabolic Pathways; Microbial metabolism in diverse environments; Carbon metabolism; Glyoxylate and dicarboxylate metabolism; Methane metabolism
K00127	0.0299	0.6186	*fdoI*; formate dehydrogenase subunit gamma	Metabolism	Metabolic Pathways; Microbial metabolism in diverse environments; Carbon metabolism; Glyoxylate and dicarboxylate metabolism; Methane metabolism
K00245	0.0299	0.5762	*frdB*; fumarate reductase iron-sulfur subunit [EC:1.3.5.4]	Metabolism; Environmental information processing	Metabolic Pathways; Two-component system; Microbial metabolism in diverse environments; Biosynthesis of secondary metabolites; Carbon metabolism; Pyruvate metabolism; Citrate cycle (TCA cycle); Carbon fixation pathways in prokaryotes; Butanoate metabolism; Oxidative phosphorylation
K00246	0.0299	0.5627	*frdC*; fumarate reductase subunit C	Metabolism; Environmental information processing	Metabolic Pathways; Two-component system; Microbial metabolism in diverse environments; Biosynthesis of secondary metabolites; Carbon metabolism; Pyruvate metabolism; Citrate cycle (TCA cycle); Carbon fixation pathways in prokaryotes; Butanoate metabolism; Oxidative phosphorylation
K00436	0.0299	0.5578	*hoxH*; NAD-reducing hydrogenase large subunit [EC:1.12.1.2]	Enzymes	Not available
K00864	0.0299	0.6190	*glpK*; glycerol kinase [EC:2.7.1.30]	Metabolism; Enzymes; Exosomes	Metabolic Pathways; Glycerolipid metabolism; Plant-pathogen interaction; PPAR signaling pathway
K01141	0.0299	0.5633	*sbcB*; exodeoxyribonuclease I [EC:3.1.11.1]	Enzymes; DNA repair and recombination proteins	Mismatch repair
K01147	0.0299	0.5632	*rnb*; exoribonuclease II [EC:3.1.13.1]	Enzymes; Transfer RNA biogenesis	Not available
K01167	0.0299	0.5578	*rnaSA*; ribonuclease T1 [EC:4.6.1.24]	Enzymes; Transfer RNA biogenesis; Messenger RNA biogenesis	Not available
K01525	0.0299	0.5632	*apaH*; bis(5'-nucleosyl)-tetraphosphatase (symmetrical) [EC:3.6.1.41]	Metabolism; Enzymes	Metabolic Pathways; Purine Metabolism
K01578	0.0299	0.5578	*mlycD*; malonyl-CoA decarboxylase [EC:4.1.1.9]	Metabolism; Enzymes	Metabolic Pathways; Propanoate metabolism; Beta-alanine metabolism; Alcoholic liver disease; Peroxisome; AMPK signaling pathway

K01678	0.0299	0.5686	*fumB*; fumarate hydratase subunit beta [EC:4.2.1.2]	Metabolism; Enzymes	Metabolic Pathways; Microbial metabolism in diverse environments; Biosynthesis of secondary metabolites; Carbon metabolism; Pyruvate metabolism; Citrate cycle (TCA cycle); Carbon fixation pathways in prokaryotes
K01914	0.0299	0.5639	*asnA*; aspartate--ammonia ligase [EC:6.3.1.1]	Metabolism; Enzymes	Metabolic Pathways; Biosynthesis of secondary metabolites; Biosynthesis of amino acids; Cyanoamino acid metabolism; Alanine, aspartate and glutamate metabolism
K01965	0.0299	0.5578	*pccA*; propionyl-CoA carboxylase alpha chain [EC:6.4.1.3]	Metabolism; Enzymes	Metabolic Pathways; Microbial metabolism in diverse environments; Biosynthesis of secondary metabolism; Carbon metabolites; Glyoxylate and dicarboxylate metabolism; Propanoate metabolism; Valine, leucine and isoleucine degradation
K02119	0.0299	0.6514	*ntpC, atpC*; V/A-type H+/Na+-transporting ATPase subunit C	Metabolism	Metabolic Pathways; Oxidative phosphorylation
K02282	0.0299	0.6211	*cpaE*; pilus assembly protein CpaE	Secretion system; Bacterial motility proteins	Not available
K02339	0.0299	0.5633	*holC*; DNA polymerase III subunit chi [EC:2.7.7.7]	Enzymes; DNA repair and recombination proteins; DNA replication proteins	Mismatch repair; Homologous recombination; DNA replication
K02569	0.0299	0.5649	*napC*; cytochrome c-type protein NapC	Metabolism	Not available
K02657	0.0299	0.5578	*pilG*; twitching motility two-component system response regulator PilG	Two-component system; Secretion system; Bacterial motility proteins	Two-component system; Biofilm formation - *Pseudomonas aeruginosa*
K02676	0.0299	0.5578	*pilZ*; type IV pilus assembly protein PilZ	Secretion system; Bacterial motility proteins	Not available
K02824	0.0299	0.6455	*uraA*; uracil permease	Transporters	Not available
K03117	0.0299	0.6759	*tatB*; sec-independent protein translocase protein TatB	Secretion system	Bacterial secretion system; Protein export
K03560	0.0299	0.5632	*tolR*; biopolymer transport protein TolR	Transporters	Not available
K03599	0.0299	0.5632	*sspA*; stringent starvation protein A	Transporters; Transcription machinery	Not available
K03600	0.0299	0.5632	*sspB*; stringent starvation protein B	Transcription machinery	Not available
K03619	0.0299	0.5603	*hyaE*; hydrogenase-1 operon protein HyaE	Not available	Not available
K03643	0.0299	0.5633	*lptE*; LPS-assembly lipoprotein	Transporters	Not available
K03646	0.0299	0.5631	*tolA*; colicin import membrane protein	Transporters	Not available
K03668	0.0299	0.5753	*hslJ*; heat shock protein HslJ	Not available	Not available
K03756	0.0299	0.6191	*potE*; putrescine:ornithine antiporter	Transporters	Not available
K03806	0.0299	0.5632	*ampD*; N-acetyl-anhydromuramoyl-L-alanine amidase [EC:3.5.1.28]	Enzymes; Peptidoglycan biosynthesis and degradation proteins	Not available
K03855	0.0299	0.5988	*fixX*; ferredoxin like protein	Not available	Not available
K03923	0.0299	0.5613	*mdaB*; NADPH dehydrogenase (quinone) [EC:1.6.5.10]	Enzymes	Not available

K03981	0.0299	0.5632	*dsbC*; thiol:disulfide interchange protein DsbC [EC:5.3.4.1]	Enzymes; Secretion system; Chaperones and folding catalysts	Not available
K04015	0.0299	0.6232	*nrfD*; protein NrfD	Not available	Not available
K04044	0.0299	0.5632	*hscA*; molecular chaperone HscA	Chaperones and folding catalysts	Not available
K04082	0.0299	0.5632	*hscB*; molecular chaperone HscB	Chaperones and folding catalysts; Mitochondrial biogenesis	Not available
K04085	0.0299	0.5637	*tusA*; tRNA 2-thiouridine synthesizing protein A [EC:2.8.1.-]	Enzymes; Transfer RNA biogenesis	Sulfur relay system
K04097	0.0299	0.5830	*HPGDS*; prostaglandin-H2 D-isomerase / glutathione transferase [EC:5.3.99.2 2.5.1.18]	Enzymes	Metabolic Pathways; Drug metabolism - cytochrome P450; Glutathione metabolism; Arachidonic acid metabolism; Chemical carcinogenesis - DNA adducts; Metabolism of xenobiotics by cytochrome P450
K04100	0.0299	0.5578	*ligA*; protocatechuate 4,5-dioxygenase, alpha chain [EC:1.13.11.8]	Enzymes; Dioxygenases	Metabolic Pathways; Microbial metabolism in diverse environments; Benzoate degradation; Polycyclic aromatic hydrocarbon degradation; Aminobenzoate degradation
K04101	0.0299	0.5578	*ligB*; protocatechuate 4,5-dioxygenase, beta chain [EC:1.13.11.8]	Enzymes; Dioxygenases	Metabolic Pathways; Microbial metabolism in diverse environments; Benzoate degradation; Polycyclic aromatic hydrocarbon degradation; Aminobenzoate degradation
K04760	0.0299	0.5632	*greB*; transcription elongation factor GreB	Transcription machinery	Not available
K05501	0.0299	0.5632	*slmA*; TetR/AcrR family transcriptional regulator	Transcription factors; Chromosome and associated proteins	Not available
K05559	0.0299	0.5578	*phaA*; multicomponent K+:H+ antiporter subunit A	Transporters	Not available
K05560	0.0299	0.5578	*phaC*; multicomponent K+:H+ antiporter subunit C	Transporters	Not available
K05561	0.0299	0.5578	*phaD*; multicomponent K+:H+ antiporter subunit D	Transporters	Not available
K05562	0.0299	0.5578	*phaE*; multicomponent K+:H+ antiporter subunit E	Transporters	Not available
K05563	0.0299	0.5578	*phaF*; multicomponent K+:H+ antiporter subunit F	Transporters	Not available
K05564	0.0299	0.5578	*phaG*; multicomponent K+:H+ antiporter subunit G	Transporters	Not available
K05805	0.0299	0.5632	*creA*; CreA protein	Not available	Not available
K05887	0.0299	0.5603	*ydiB*; quinate/shikimate dehydrogenase [EC:1.1.1.282]	Enzymes	Metabolic Pathways; Biosynthesis of secondary metabolites; Phenylalanine, tyrosine and tryptophan biosynthesis
K05962	0.0299	0.5578	Uncharacterized protein	Not available	Not available
K06186	0.0299	0.5632	*bamE*; outer membrane protein assembly factor BamE	Transporters	Not available
K06189	0.0299	0.5632	*corC*; hemolysin (HlyC) family protein	Transporters	Not available

K06190	0.0299	0.5632	*ispZ*; intracellular septation protein	Not available	Not available
K06202	0.0299	0.5632	*cyaY*; iron-sulfur cluster assembly protein CyaY	Not available	Not available
K06879	0.0299	0.5641	*queF*; 7-cyano-7-deazaguanine reductase [EC:1.7.1.13]	Enzymes; Transfer RNA biogenesis	Metabolic Pathways; Folate biosynthesis
K07014	0.0299	0.5632	Uncharacterized protein	Not available	Not available
K07091	0.0299	0.5633	*lptF*; lipopolysaccharide export system permease protein	Transporters	ABC transporters
K07130	0.0299	0.5719	*kynB*; arylformamidase [EC:3.5.1.9]	Enzymes	Metabolic Pathways; Glyoxylate and dicarboxylate metabolism; Biosynthesis of cofactors; Tryptophan metabolism
K07178	0.0299	0.5578	*RIOK1*; RIO kinase 1 [EC:2.7.11.1]	Enzymes; Ribosome biogenesis; Protein kinases	Ribosome biogenesis in eukaryotes
K07227	0.0299	0.5658	*chuX*; heme iron utilization protein	Not available	Not available
K07278	0.0299	0.5623	*tamA*; translocation and assembly module TamA	Transporters	Not available
K07287	0.0299	0.5632	*bamC*; outer membrane protein assembly factor BamC	Transporters	Not available
K07320	0.0299	0.5632	*prmB*; ribosomal protein L3 glutamine methyltransferase [EC:2.1.1.298]	Enzymes; Ribosome biogenesis	Not available
K07323	0.0299	0.5632	*mlaC*; phospholipid transport system substrate-binding protein	Transporters	ABC transporters
K07489	0.0299	0.5950	Transposase	Not available	Not available
K07667	0.0299	0.6350	*kdpE*; two-component system, OmpR family, KDP operon response regulator KdpE	Two-component system	Two-component system; Quorum sensing
K07673	0.0299	0.5632	*narX*; two-component system, NarL family, nitrate/nitrite sensor histidine kinase NarX [EC:2.7.13.3]	Enzymes; Two-component system; Protein kinases	Two-component system
K07674	0.0299	0.5599	*narQ*; two-component system, NarL family, nitrate/nitrite sensor histidine kinase NarQ [EC:2.7.13.3]	Enzymes; Two-component system; Protein kinases	Two-component system
K07684	0.0299	0.6014	*narL*; two-component system, NarL family, nitrate/nitrite response regulator NarL	Two-component system	Two-component system
K07708	0.0299	0.5632	*glnL*; two-component system, NtrC family, nitrogen regulation sensor histidine kinase GlnL [EC:2.7.13.3]	Enzymes; Two-component system; Protein kinases	Two-component system
K08082	0.0299	0.5578	*algZ*; two-component system, LytTR family, sensor histidine kinase AlgZ [EC:2.7.13.3]	Enzymes; Two-component system; Protein kinases	Two-component system
K08083	0.0299	0.5578	*algR*; two-component system, LytTR family, response regulator AlgR	Two-component system	Two-component system
K08304	0.0299	0.5633	*mltA*; membrane-bound lytic murein transglycosylase A [EC:4.2.2.-]	Enzymes; Peptidoglycan biosynthesis and degradation proteins	Not available

K09005	0.0299	0.6427	Uncharacterized protein	Not available	Not available
K09017	0.0299	0.6466	*rutR*; TetR/AcrR family transcriptional regulator	Transcription factors	Not available
K09136	0.0299	0.6389	*ycaO*; ribosomal protein S12 methylthiotransferase accessory factor	Ribosome biogenesis	Not available
K09252	0.0299	0.5578	*FAEB*; feruloyl esterase [EC:3.1.1.73]	Enzymes	Not available
K09800	0.0299	0.5632	*tamB*; translocation and assembly module TamB	Transporters	Not available
K09801	0.0299	0.5636	Uncharacterized protein	Not available	Not available
K09858	0.0299	0.5640	SEC-C motif domain protein	Not available	Not available
K09862	0.0299	0.5636	Uncharacterized protein	Not available	Not available
K09889	0.0299	0.5632	*yjgA*; ribosome-associated protein	Ribosome biogenesis	Not available
K09919	0.0299	0.5628	Uncharacterized protein	Not available	Not available
K09969	0.0299	0.5595	*aapJ*; general L-amino acid transport system substrate-binding protein	Transporters	ABC transporters
K09970	0.0299	0.5654	*aapQ*; general L-amino acid transport system permease protein	Transporters	ABC transporters
K09971	0.0299	0.5596	*aapM*; general L-amino acid transport system permease protein	Transporters	ABC transporters
K10001	0.0299	0.5632	*gltI*; glutamate/aspartate transport system substrate-binding protein	Transporters	Two-component system; ABC transporters
K10002	0.0299	0.5631	*gltK*; glutamate/aspartate transport system permease protein	Transporters	Two-component system; ABC transporters
K10003	0.0299	0.5632	*gltJ*; glutamate/aspartate transport system permease protein	Transporters	Two-component system; ABC transporters
K10004	0.0299	0.5631	*gltL*; glutamate/aspartate transport system ATP-binding protein [EC:7.4.2.1]	Enzymes; Transporters	Two-component system; ABC transporters
K10039	0.0299	0.6277	*peb1A*; aspartate/glutamate/glutamine transport system substrate-binding protein	Transporters	ABC transporters
K10763	0.0299	0.5632	*hda*; DnaA-homolog protein	DNA replication proteins	Not available
K10764	0.0299	0.5578	*metZ*; O-succinylhomoserine sulfhydrylase [EC:2.5.1]	Enzymes	Metabolic Pathways; Cysteine and methionine metabolism; Sulfur metabolism
K11250	0.0299	0.5659	*leuE*; leucine efflux protein	Transporters	Not available
K12297	0.0299	0.5905	*rlmKL*; 23S rRNA (guanine2069-N7)-methyltransferase / 23S rRNA (guanine2445-N2)-methyltransferase [EC:2.1.1.264 2.1.1.173]	Enzymes; Ribosome biogenesis	Not available
K12339	0.0299	0.5586	*cysM*; S-sulfo-L-cysteine synthase (O-acetyl-L-serine-dependent) [EC:2.5.1.144]	Enzymes	Metabolic Pathways; Cysteine and methionine metabolism; Biosynthesis of amino acids

K12508	0.0299	0.5578	*fcs*; feruloyl-CoA synthase [EC:6.2.1.34]	Enzymes	Not available
K14058	0.0299	0.5633	*ttcA*; tRNA 2-thiocytidine biosynthesis protein TtcA	Transfer RNA biogenesis	Not available
K00390	0.0301	0.5570	*cysH*; phosphoadenosine phosphosulfate reductase [EC:1.8.4.8 1.8.4.10]	Enzymes	Metabolic Pathways; Microbial metabolism in diverse environments; Sulfur metabolism
K03673	0.0312	0.5551	*dsbA*; protein dithiol oxidoreductase (disulfide-forming) [EC:1.8.4.15]	Enzymes; Chaperones and folding catalysts	Cationic antimicrobial peptide (CAMP) resistance
K03746	0.0313	0.5537	*hns*; DNA-binding protein H-NS	DNA repair and recombination proteins; Chromosome and associated proteins	Not available
K05964	0.0313	0.5541	*citX*; holo-ACP synthase [EC:2.7.7.61]	Enzymes	Two-component system
K11904	0.0313	0.5545	*vgrG*; type VI secretion system secreted protein VgrG	Secretion system	Bacterial secretion system
K12507	0.0336	0.5497	*fadK*; acyl-CoA synthetase [EC:6.2.1.-]	Enzymes; Lipid biosynthesis proteins	Not available
K13590	0.0336	0.5498	*dgcB*; diguanylate cyclase [EC:2.7.7.65]	Enzymes	Cell cycle - *Caulobacter*
K02670	0.0359	0.5459	*pilU*; twitching motility protein PilU	Secretion system; Bacterial motility proteins	Not available
K03821	0.0359	0.5459	*phaC*; poly[(R)-3-hydroxyalkanoate] polymerase subunit PhaC [EC:2.3.1.304]	Enzymes	Metabolic Pathways; Butanoate metabolism
K02199	0.0373	0.5438	*ccmG*; cytochrome c biogenesis protein CcmG, thiol:disulfide interchange protein DsbE	Chaperones and folding catalysts	Not available
K04719	0.0375	0.5432	*bluB*; 5,6-dimethylbenzimidazole synthase [EC:1.13.11.79]	Enzymes	Metabolic Pathways; Biosynthesis of cofactors; Riboflavin metabolism
K13288	0.0377	0.5426	*orn*; oligoribonuclease [EC:3.1.-.-]	Enzymes; Ribosome biogenesis; Messenger RNA biogenesis	Ribosome biogenesis in eukaryotes
K07039	0.0397	0.5397	Uncharacterized protein	Not available	Not available
K00945	0.0401	0.5385	*cmk*; CMP/dCMP kinase [EC:2.7.4.25]	Enzymes	Metabolic Pathways; Pyrimidine metabolism; Nucleotide metabolism
K03760	0.0401	0.5375	*eptA*; lipid A ethanolaminephosphotransferase [EC:2.7.8.43]	Enzymes; Lipopolysaccharide biosynthesis proteins	Metabolic Pathways; Cationic antimicrobial peptide (CAMP) resistance; Lipopolysaccharide biosynthesis
K07262	0.0401	0.5374	*pbpG*; serine-type D-Ala-D-Ala endopeptidase (penicillin-binding protein 7) [EC:3.4.21.-]	Enzymes; Peptidoglycan biosynthesis and degradation proteins; Peptidases and inhibitors	Not available
K07810	0.0401	0.5389	*cusF*; Cu(I)/Ag(I) efflux system periplasmic protein CusF	Not available	Two-component system
K09937	0.0401	0.5374	Uncharacterized protein	Not available	Not available
K03757	0.0401	0.5366	*cadB*; cadaverine:lysine antiporter	Transporters	Not available
K05834	0.0401	0.5367	*rhtB*; homoserine/homoserine lactone efflux protein	Transporters	Not available

K06995	0.0410	0.5353	Uncharacterized protein	Not available	Not available
K01463	0.0419	0.5338	*bshB1*; N-acetylglucosamine malate deacetylase 1 [EC:3.5.1.-]	Enzymes	Not available
K03326	0.0459	0.5292	*dcuC, dcuD*; C4-dicarboxylate transporter, DcuC family	Transporters	Not available
K05539	0.0485	0.5263	*dusA*; tRNA-dihydrouridine synthase A [EC:1.-.-.-]	Enzymes; Transfer RNA biogenesis	Not available

[a]*P*-values are reported following analysis in STAMP by ANOVA with Storey's False Discovery Rate (FDR) used for multiple test corrections. PICRUSt, Phylogenetic Investigation of Communities by Reconstruction of Unobserved States; C-PAC, cranberry proanthocyanidins; OTU ID, operation taxonomic unit identifier; KEGG, Kyoto Encyclopedia of Genes and Genomes; KO, KEGG ontology.

Supplemental Table 17. Antibodies utilized in this research.

Protein	Company	Catalog Number	Dilution
ABCB1	Abcam	ab170904	1:500
CD44	Abcam	ab189524	1:500
COX-2	Abcam	ab15191	1:100
GAPDH	Cell Signaling	2118	1:20,000
HPRT	Santa Cruz	sc-20975	1:750
IL-1β	Abcam	ab9722	1:500
IL-8	Novus Biologicals	MAB208-100	1:500
MyD88	Cell Signaling	4283	1:1000
NFκ-B1	Cell Signaling	13586	1:400
TP53	Millipore	OP43	1:1000
PCNA	Santa Cruz	sc-7907	1:200
Phospho-ERK1/2$^{T202/Y185}$	Cell Signaling	4370	1:1000
Phospho-P38$^{T180/Y192}$	Cell Signaling	4511	1:1000
Phospho-SAPK/JNK$^{T182/Y185}$	Cell Signaling	4668	1:1000
RXRɣ	Cell Signaling	5629	1:1000
TLR3	Novus Biologicals	NBP2-24565	1:500
TP53I3	Exalpha	X1155P	1:500
