# Supplementary material for: Prebiotic proanthocyanidins inhibit bile reflux–induced esophageal adenocarcinoma through reshaping the gut microbiome and esophageal metabolome
Source: JCI Insight. 2024 Feb 8;9(6):e168112. doi: 10.1172/jci.insight.168112 (PMC11063939; doi:10.1172/jci.insight.168112)

Prebiotic proanthocyanidins inhibit bile reflux-induced esophageal adenocarcinoma through reshaping the gut microbiome and esophageal metabolome

Katherine M. Weh, Connor L. Howard, Yun Zhang, Bridget A. Trip, Jennifer L. Clarke, Amy B. Howell, Joel H. Rubenstein, Julian A. Abrams, Maria Westerhoff and Laura A. Kresty

Unedited/uncropped western blot images for data presented in manuscript
Figure 6 - entire figure
Figure 7 - panel A

Complete Figure 6

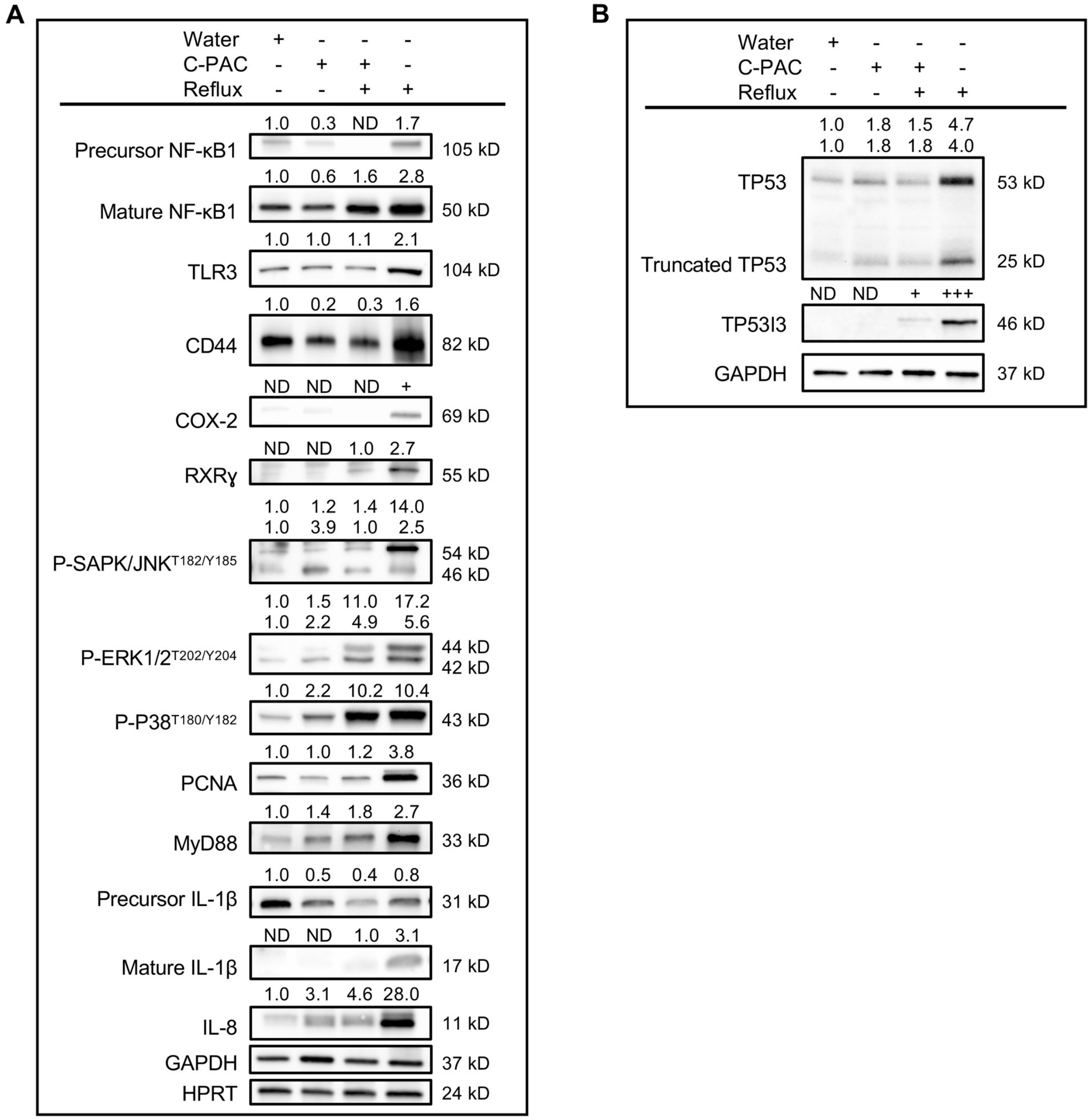

Figure 6A.
NF-KB1
Cell Signaling
Catalog #13586
1:400 dilution

Precursor NF-κB1

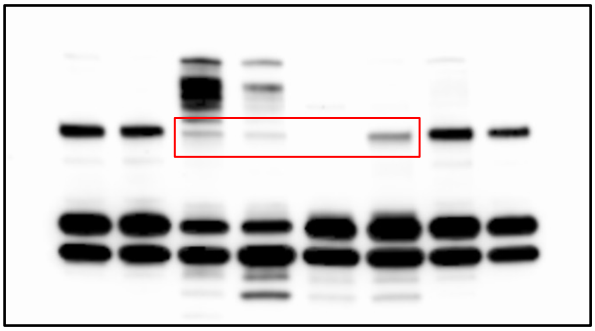

Mature NF-κB1

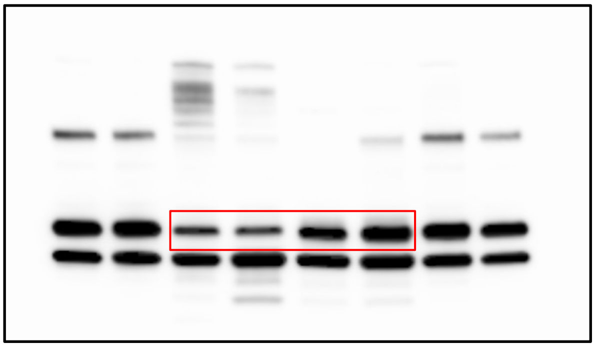

Figure 6A.
TLR3
Novus Biologicals
Catalog #NBP2-24565
1:500 dilution

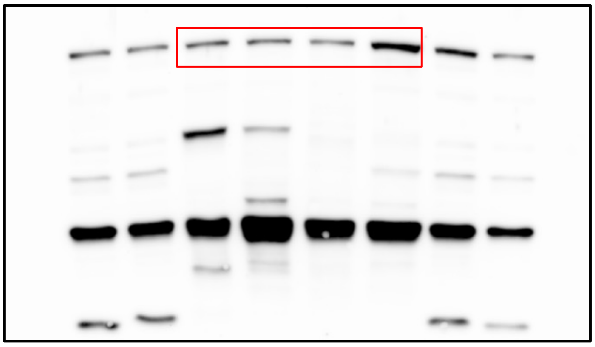

Figure 6A.
CD44
Abcam
Catalog #ab189524
1:500 dilution

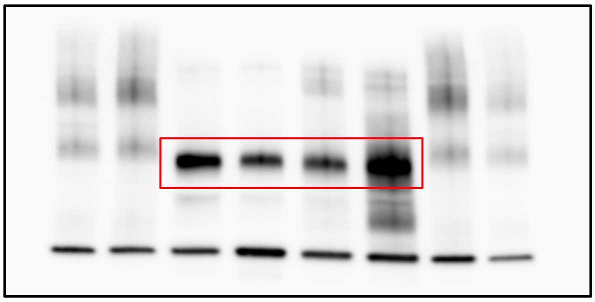

Figure 6A.
CD44
Abcam
Catalog #ab189524
1:500 dilution

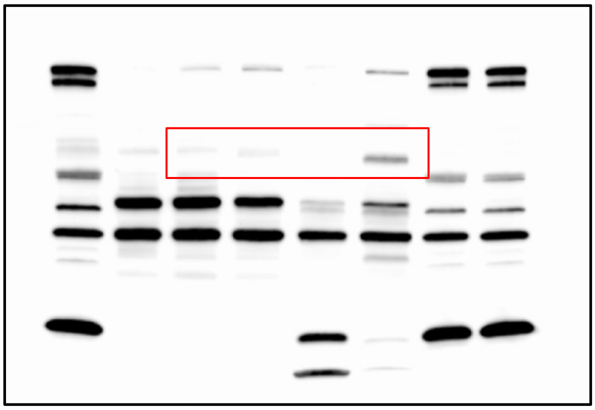

Figure 6A.
RXRɣ
Cell Signaling
Catalog #5629
1:1000

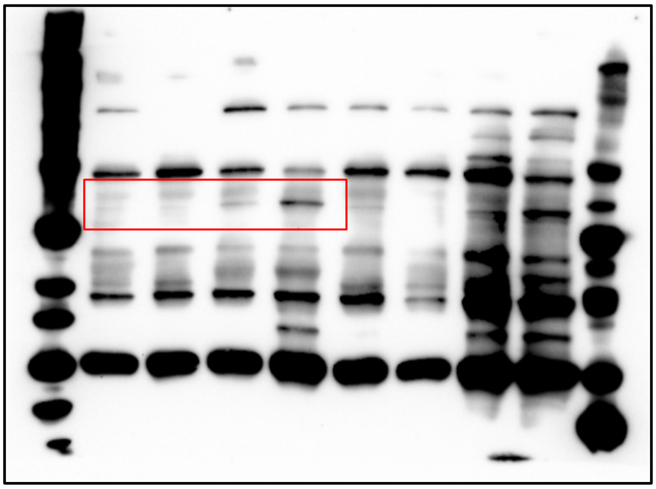

Figure 6A.
Phospho-SAPK/JNK$^{T182/Y185}$
Cell Signaling
Catalog #4668
1:1000

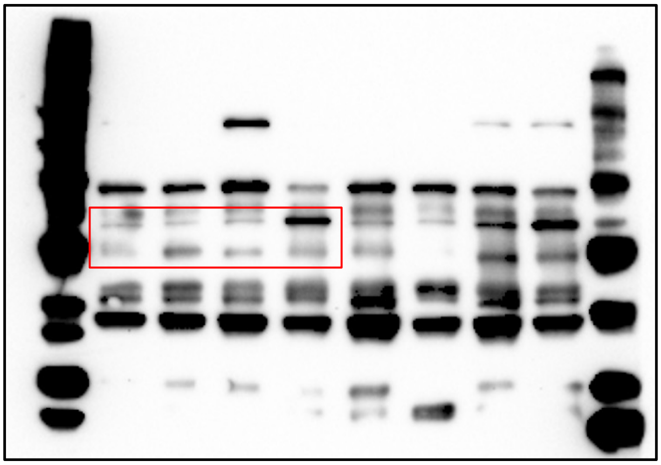

Figure 6A.
Phospho-ERK1/2$^{T202/Y185}$
Cell Signaling
Catalog #4370
1:1000

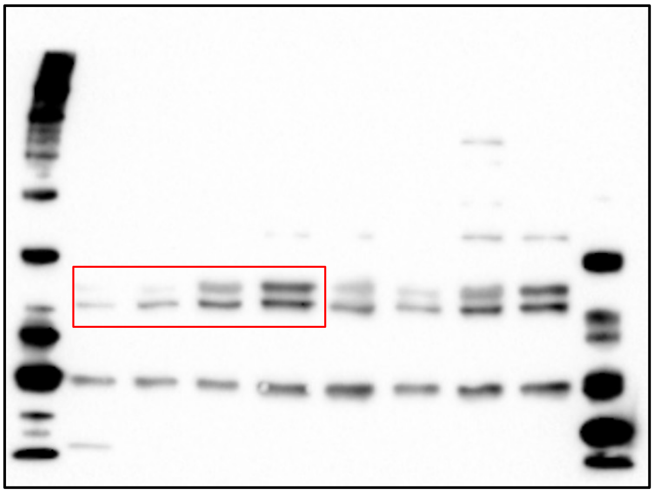

Figure 6A.
Phospho-P38$^{T180/Y192}$
Cell Signaling
Catalog #4511
1:1000

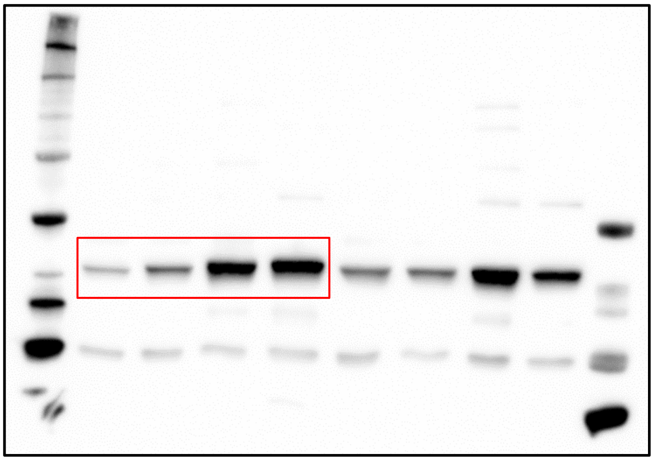

Figure 6A.
PCNA
Santa Cruz
Catalog #sc-7907
1:200

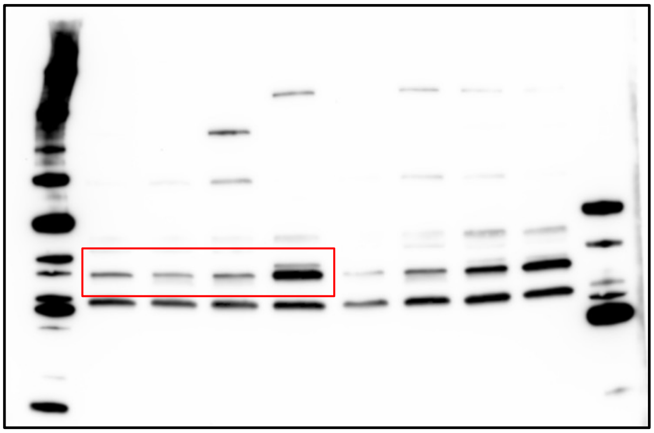

Figure 6A.
MyD88
Cell Signaling
Catalog #4283
1:1000

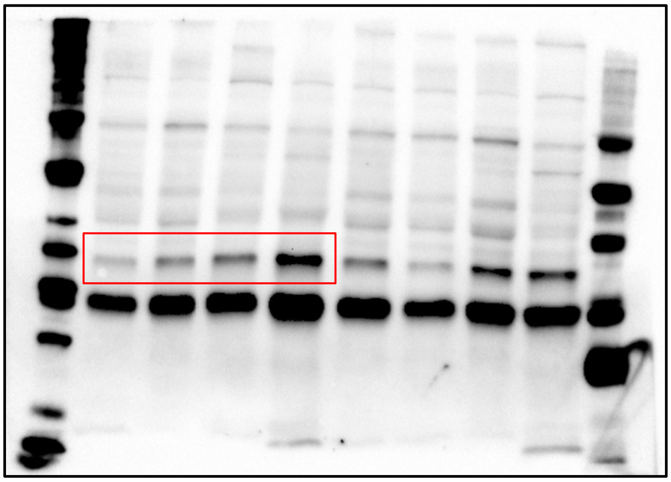

Figure 6A.
IL-1β
Abcam
Catalog #ab9722
1:500

Precursor IL-1β

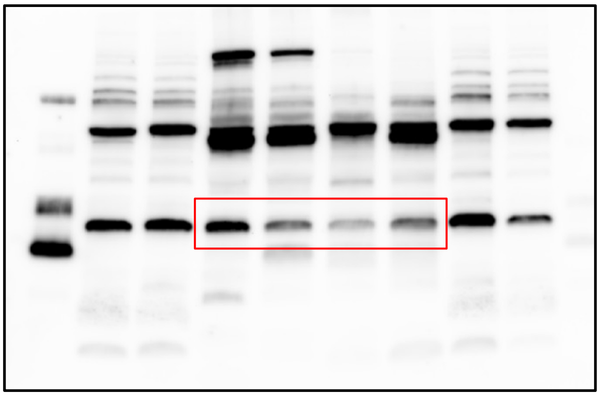

Mature IL-1β

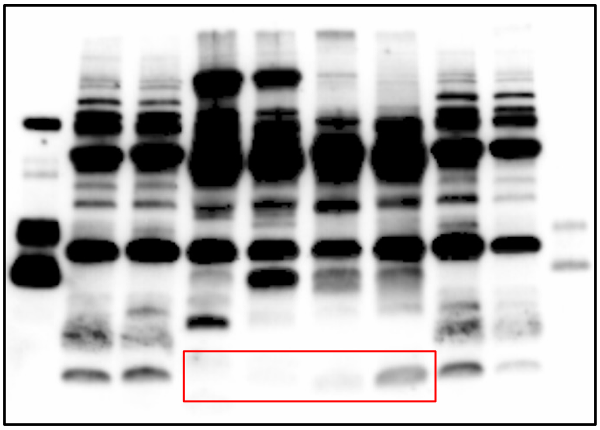

Figure 6A.
IL-8
Novus Biologicals
Catalog #MAB208-100
1:500

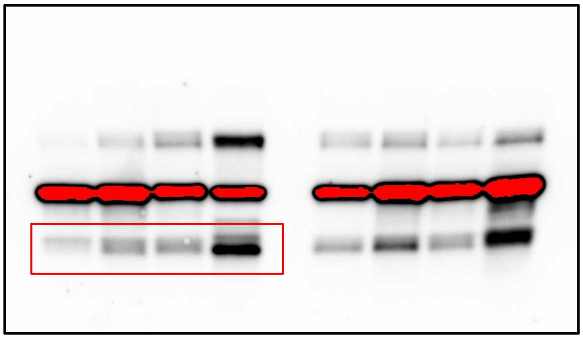

Figure 6A.
GAPDH
Cell Signaling
Catalog #2118
1:20,000

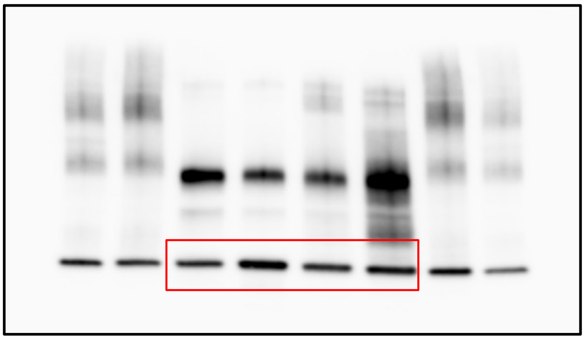

Figure 6A.
HPRT
Santa Cruz
Catalog #sc-20975
1:750

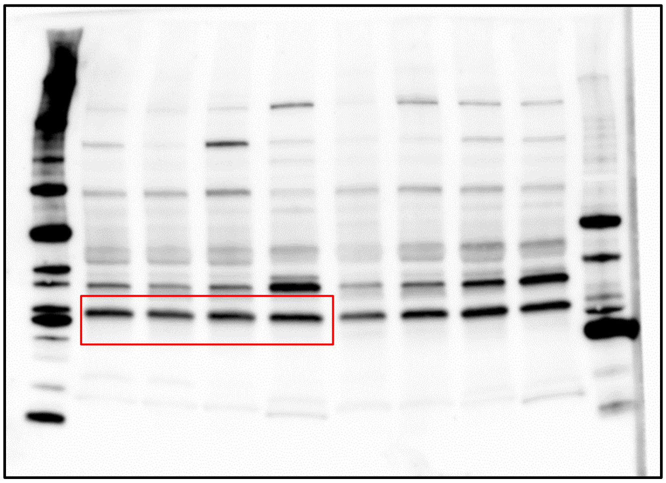

Figure 6B.
P53
Millipore
Catalog #OP43
1:1000

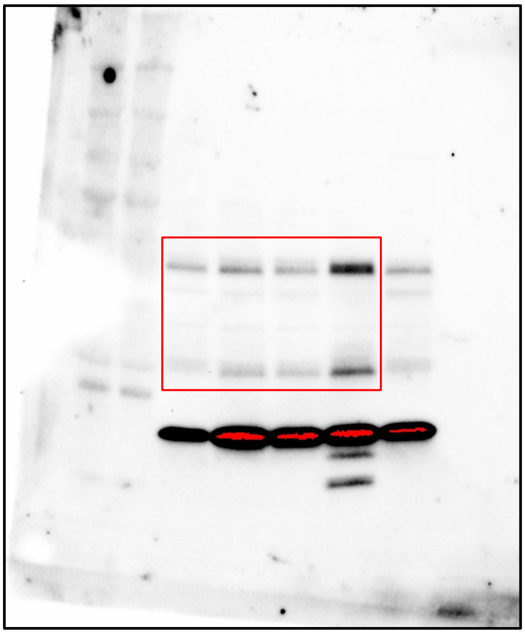

Figure 6B.
TP53I3
exalpha
Catalog #X1155P
1:500

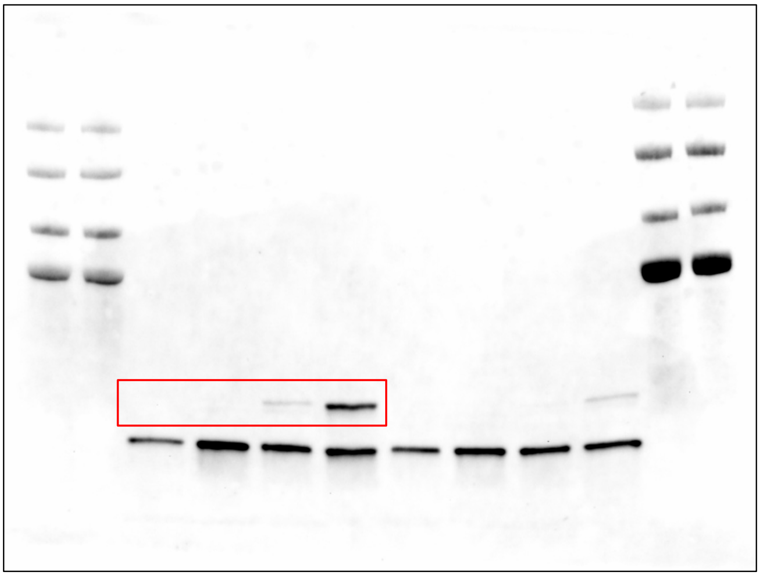

Figure 6B.
GAPDH
Cell Signaling
Catalog #2118
1:20,000

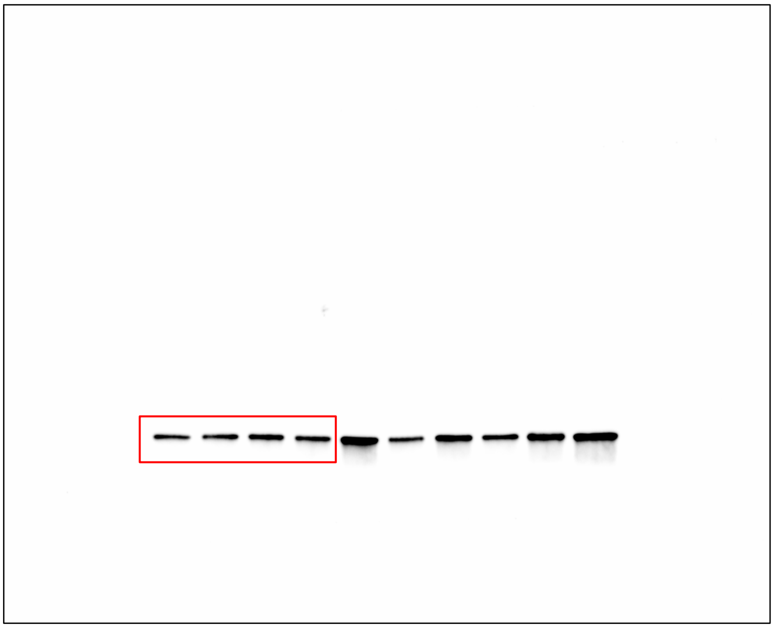

Complete Figure 7

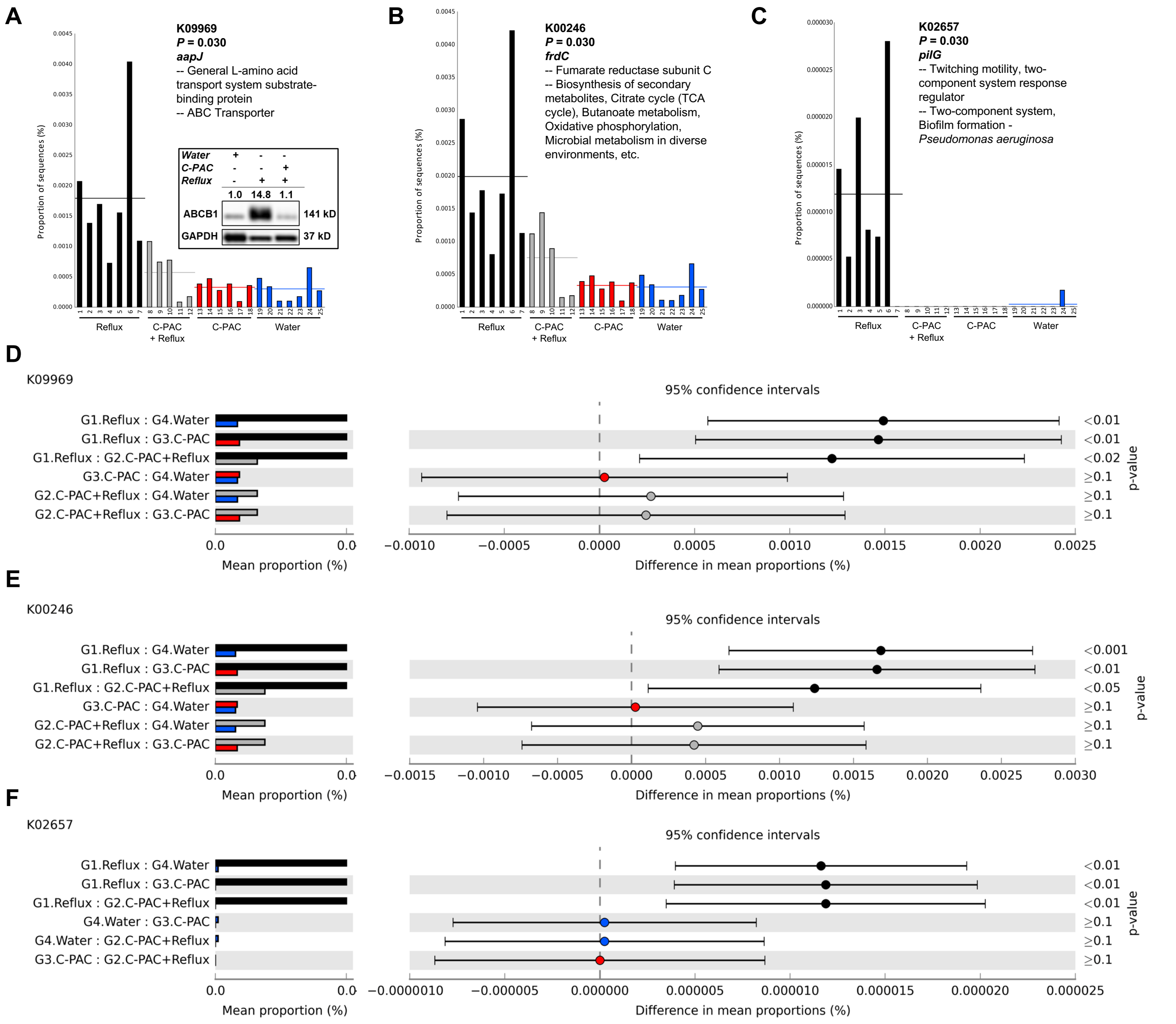

Figure 7A.
ABCB1
Abcam
Catalog #ab170904
1:500

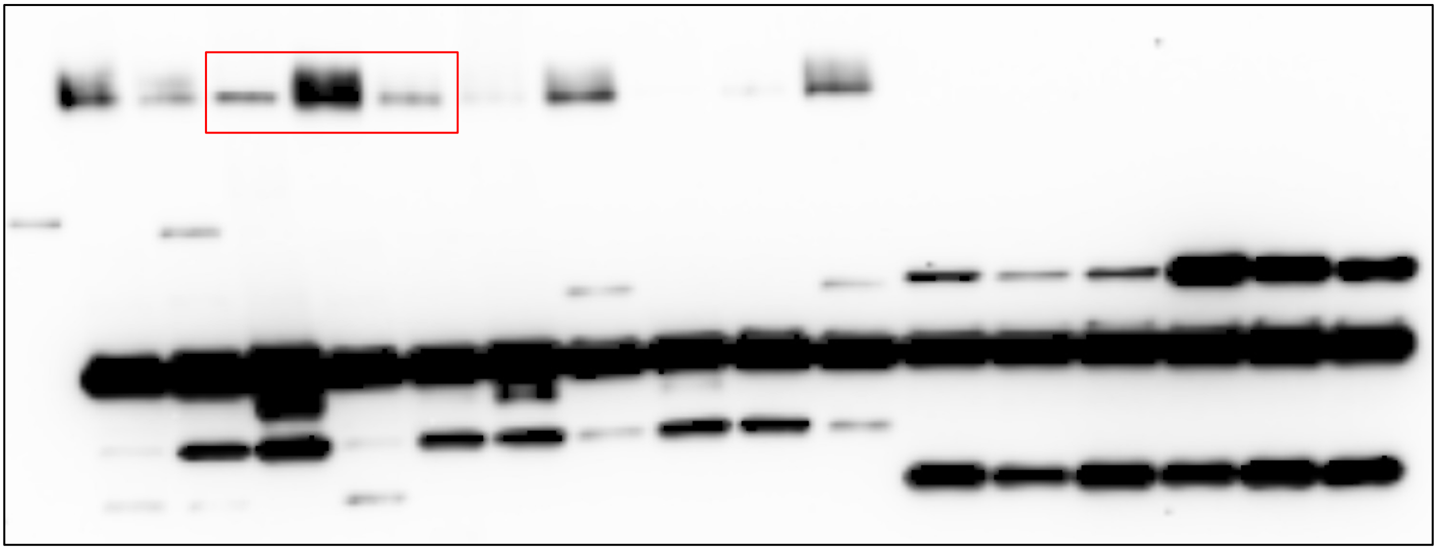

Figure 7A.
GAPDH
Cell Signaling
Catalog #2118
1:20,000

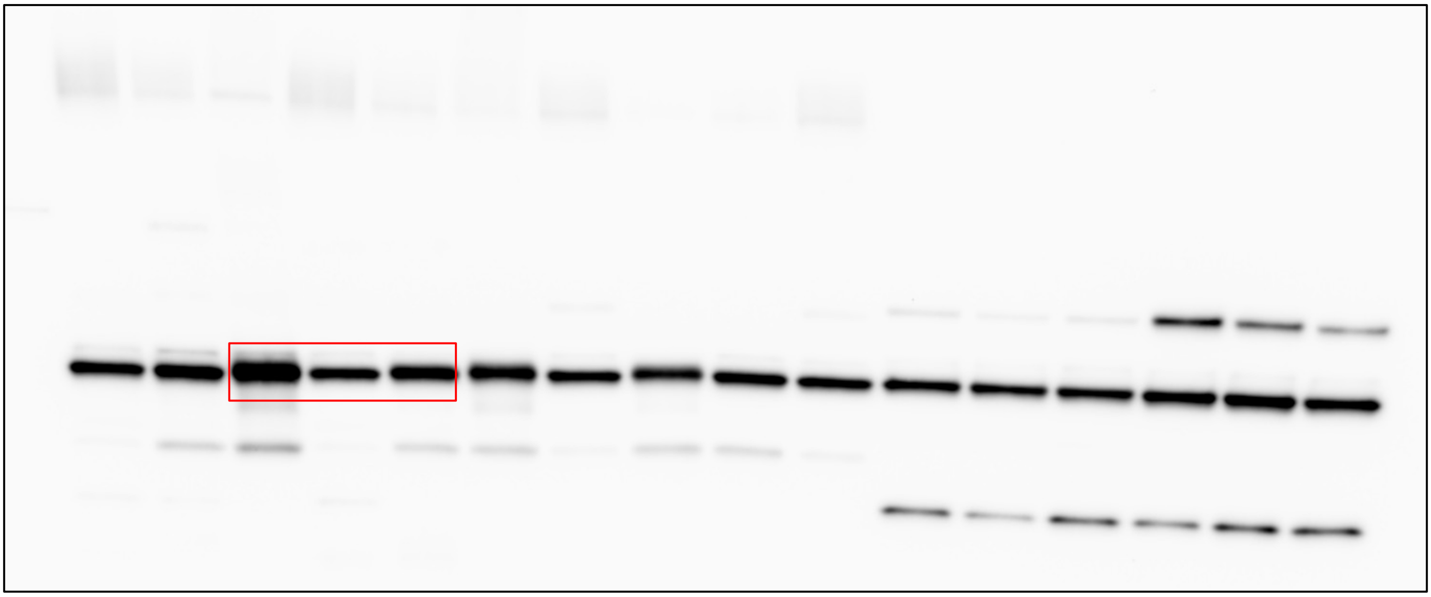

Supplement: Unedited blot and gel images [file jciinsight-9-168112-s047.pdf]
